# Benchmarking Intrinsic Promoters and Terminators for Plant Synthetic Biology Research

**DOI:** 10.34133/2022/9834989

**Published:** 2022-05-26

**Authors:** Chenfei Tian, Yixin Zhang, Jianhua Li, Yong Wang

**Affiliations:** ^1^CAS-Key Laboratory of Synthetic Biology, CAS Center for Excellence in Molecular Plant Sciences, Institute of Plant Physiology and Ecology, Chinese Academy of Sciences, Shanghai 200032, China; ^2^University of Chinese Academy of Sciences, Beijing 100039, China; ^3^College of Life Science, Jilin Agricultural University, Changchun 130118, China

## Abstract

The emerging plant synthetic metabolic engineering has been exhibiting great promise to produce either value-added metabolites or therapeutic proteins. However, promoters for plant pathway engineering are generally selected empirically. The quantitative characterization of plant-based promoters is essential for optimal control of gene expression in plant chassis. Here, we used *N. benthamiana* leaves and BY2 suspension cells to quantitatively characterize a library of plant promoters by transient expression of firefly/*Renilla* luciferase. We validated the dual-luciferase reporter system by examining the correlation between reporter protein and mRNA levels. In addition, we investigated the effects of terminator–promoter combinations on gene expression and found that the combinations of promoters and terminators resulted in a 326-fold difference between the strongest and weakest performance, as reflected in reporter gene expression. As a proof of concept, we used the quantitatively characterized promoters to engineer the betalain pathway in *N. benthamiana*. Seven selected plant promoters with different expression strengths were used orthogonally to express CYP76AD1 and DODA, resulting in a final betalain production range of 6.0–362.4 *μ*g/g fresh weight. Our systematic approach not only demonstrates the various intensities of multiple promoter sequences in *N. benthamiana* and BY2 cells but also adds to the toolbox of plant promoters for plant engineering.

## 1. Introduction

Synthetic biology has become a valuable biotechnological approach for synthesizing a wide range of pharmaceutical, nutraceutical, and industrial products in a heterologous chassis [1–7]. Although notable successes have been achieved in microbial systems [3–5, 7], synthetic biology in plants is still an emerging research field with many knowledge and technology gaps that remain to be addressed. It is widely recognized that plants have the advantages of photoautotrophic utilization machinery and environmentally sustainable bioproduction [8, 9]. However, their relatively slow growth, the lack of well-established genetic parts for genetic manipulation, and their genomic complexity have hindered the widespread adoption of plants as synthetic biology chassis.

To date, multiple plant systems, such as tomato, lettuce, rice, and tobacco, have been successfully utilized to produce either value-added metabolites or therapeutic proteins [10–13]. For example, a high level of L-DOPA was produced in tomato fruit through the introduction of BvCYP76AD6 [12]. Unlike whole plants, suspension-cultured plant cells are regarded as an attractive platform because of their amenability to industrial-scale batch culture and ease of compliance with good manufacturing practice (GMP) [14–16]. For example, *Oryza sativa* suspension cells have been used to produce human *α*1-antitrypsin [15], and cultured BY2 cells have been used to produce the ORF8 protein from SARS-CoV-2, a potential therapeutic agent against COVID-19 [16]. All these novel traits have been obtained by introducing a single gene or set of genes into engineered plants under the control of appropriate promoters.

The plant promoter is a cis-acting DNA fragment that initiates the transcription of an associated gene [17]. It is largely responsible for the specificity (species, spatial, or temporal) and intensity of gene expression [17]. However, to date, plant engineering has relied heavily on the repeated use of a few well-established constitutive promoters, such as the Ubi promoter from maize, the CaMV 35S promoter from cauliflower mosaic virus, and the NOS promoter from *Agrobacterium*, to drive gene expression. With the recent availability of plant genome sequences and the development of bioinformatics approaches, the number of promoter sequences has increased significantly over the past few decades. For example, more than 8,000 promoter sequences are available at the plant promoter database (PlantProm DB; http://linux1.softberry.com/berry.phtml). However, only a small number of promoters have been experimentally characterized in plant chassis. Previous studies on promoter characterization have focused on specific plant cells or tissues, such as maize protoplasts [18], *Arabidopsis* protoplasts [19], *N. benthamiana* leaves [20], and lima bean cotyledons [21], yet the performance and the relative usefulness of individual promoter sequences across plant species have not been widely investigated.

Although single-gene engineering has been widely used for plant genetic improvement, multigene engineering will become more and more important for plant synthetic biology research in the future. Plant synthetic metabolic engineering focuses on the introduction of a complete synthetic pathway into the plant. For example, a number of synthetic metabolic pathways for the biosynthesis of natural products have been successfully reconstructed in *N. benthamiana* through transient expression [22–24]. However, the repeated use of a promoter in a multigene construct can cause gene silencing in transgenic plants [25]. In addition, each recombinant protein is equally expressed at the same level, and this may lead to a metabolic flux imbalance, causing extremely low yields [26, 27]. Therefore, for the stable transformation of multiple genes into plants, each single gene should be under the control of a different promoter. However, promoters for plant pathway engineering are generally selected empirically, with no quantitative information on promoter strength, and this approach is insufficient for the precise control of gene expression to maximize metabolite yields. Therefore, the quantitative characterization of plant-based promoters is essential for the rational design of multigene pathways to achieve optimal control of gene expression.

By fusing a GUS (*β*-glucuronidase) or GFP (green fluorescent protein) gene to a promoter sequence, promoter activity can be monitored based on the activity of the reporter gene product [28–31]. However, the outputs of GUS or GFP reporters are unstable and not suitable for quantitative assays. Recently, a ratiometric dual reporter system based on luciferase was developed for the rapid characterization of genetic parts [32–34]. Nonetheless, several issues remain unresolved: (1) variable performance of promoters across different plant tissues [21, 28], (2) low correlations between reporter protein expression and the abundance of corresponding mRNAs [35], and (3) effects of sampling time point on promoter performance [35].

Here, we used *N. benthamiana* leaves and BY2 suspension cells to quantitatively characterize a library of plant promoters by transient expression of firefly/*Renilla* luciferase. We validated the dual-luciferase reporter system by examining the correlation between reporter protein and mRNA levels. In addition, we investigated the effects of terminator–promoter combinations on gene expression and found that the combinations of promoters and terminators resulted in a 326-fold difference between the strongest and weakest performance, as reflected in reporter gene expression. Finally, as a proof of concept, we used the quantitatively characterized promoters to engineer the betalain pathway in *N. benthamiana*.

## 2. Materials and Methods

### 2.1. Plant Materials

*N. benthamiana* plants were grown in soil in a greenhouse with a 16 h light/8 h dark photoperiod at 25°C. The BY2 cells were cultured in darkness at 25°C with a BY2 medium (BY2 medium PM1591 (Coolaber), 3% sucrose, pH 5.7). BY2 cells were maintained in 100 mL Erlenmeyer flasks aerated by shaking at 120 rpm and subcultured every 7 days by inoculating 5 mL of suspension cells into 25 mL of fresh medium.

### 2.2. Plasmid Construction

To facilitate the large amount of DNA assembly needed in this work, all basic DNA parts were cloned into plasmids with standard base ends for further subcloning into plant expression vectors using the GoldenBraid methods [34]. We cloned promoters (Table S1) and terminators (Table S2) from multiple plant species, including *Arabidopsis thaliana*, *Chrysanthemum morifolium*, *Marchantia polymorpha*, *O. sativa*, *Solanum tuberosum*, and *Zea mays*. Total DNA was extracted from leaves using a Plant Genomic DNA Kit (DP305-02, Transgen). Sequences were amplified with PrimeSTAR Max DNA polymerase (R045A, TaKaRa) using the primers listed in Table S3. Some sequences were synthesized directly with standard recognized sites (General Biol, Anhui). All promoters were cloned into pEASY (Transgen) flanked by GGAG and AATG fusion sites recognized by *Bsa*I/*Bsm*BI, all terminators were cloned into pEASY flanked by GCTT and CGCT fusion sites, and all CDS sequences were cloned into pEASY flanked by AATG and GCTT (Figure S1(a)). The basic vector pCF001 was modified from pEAQ-HT-GG, whose restriction sites were replaced with GGAG and CGCT fusion sites recognized by *Bsa*I. Plasmids with a single expression cassette were constructed by ligating the pCF001 vector, promoter, CDS, and terminator fragments after *Bsa*I/*Bsm*BI digestion with T4 DNA ligase (Thermo). Dual-luciferase reporter plasmids were constructed based on the P_CsVMV::R-luc::T_nos reporter plasmid. The vector pCF43 used to assemble the firefly luciferase expression cassette with different regulatory elements was obtained by adding a *Bsa*I recognition site with a standard fusion site by PCR amplification (Figure S1(b)).

The synthetic genes CYP76AD1 (HQ656023.1), DODA (HQ656027.1), and DOPA5GT (AB182643.1) were codon-optimized for *N. benthamiana* and then synthesized, flanked by two pairs of *Bsa*I.

### 2.3. Transient Expression in *N. benthamiana*

All binary plasmids were introduced into *Agrobacterium tumefaciens* strain GV3101. *A. tumefaciens* was grown overnight at 28°C and 220 rpm in LB with 50 mg/L kanamycin, 25 mg/L rifampicin, and 25 mg/L gentamycin. The overnight culture was centrifuged at 6000 rpm for 6 min and then suspended in MMA buffer containing 10 mM MES (2-[N-morpholino]-ethanesulfonic acid, Sangon Biotech), 10 mM MgCl_2_, and 100 *μ*M acetosyringone to a final OD_600_ of 1.0. The strain was incubated at room temperature for 3 h before infiltration into 4-month-old *N. benthamiana* leaves. For co-infiltration assays, equal volumes of the *A. tumefaciens* cultures were mixed before infiltration.

### 2.4. GFP Expression Assays

Forty-eight hours after infiltration, at least three leaves were placed on slides for direct observation using an Olympus BX63 microscope equipped with a DP73 digital camera at an excitation wavelength of 488 nm. The images were processed with ImageJ [36].

### 2.5. Dual-Luciferase Expression Assays

Forty-eight hours after infiltration, at least three leaf samples (ca. 1–2 cm in diameter) were collected for a dual-luciferase assay using the commercial Dual-Luciferase Reporter Assay System (E1910, Promega). Each sample was frozen with liquid nitrogen and ground into a fine powder with a grinder (Wonbio-48RS, WONBIO) and then homogenized in 100 *μ*L of Passive Lysis Buffer (Promega). The activities of firefly luciferase and *Renilla* luciferase were measured with a luminometer (GloMax 20/20, Promega). The relative promoter activity was defined as the ratio of firefly luminescence intensity to *Renilla* luminescence intensity.

### 2.6. Real-Time Quantitative Reverse Transcription PCR

Total RNA was isolated from leaf samples (150 mg) using the RNA Easy Fast Plant Tissue Kit (DP452, Tiangen), and 1 ng total RNA was used for cDNA synthesis with SuperScript (Invitrogen). Quantitative real-time PCR was performed using SYBR Premix Ex Taq (RR420L, TaKaRa) on the StepOne Plus system (Applied Biosystems). Primers for target gene amplification were designed using Primer Premier 5.0, and all primers are listed in Table S3.

### 2.7. Transient Expression in BY2 Cells

The constructs were transformed into BY2 cells via *Agrobacterium tumefaciens* GV3101. After washing twice in fresh liquid BY2 medium with 150 *μ*M acetosyringone, a 1 mL aliquot of 4-day-old BY2 cells was cocultured with 1 mL of strain GV3101 ($\text{O}{\text{D}}_{600}=1$) carrying the target plasmid. After four days of cocultivation on BY2 solid medium in the dark, the BY2 cells were washed with double-distilled water and collected for dual-luciferase assays.

### 2.8. Betalain Extraction and Quantification

Sixty hours after infiltration, at least three samples (0.1 g) were collected from different leaves. Betalain was extracted with extraction solution (80% ethanol and 0.1% formic acid in double-distilled water). Samples were ground into powder in liquid nitrogen and extracted in extraction solution overnight at 4°C after 5 min of sonication. The supernatant was obtained after centrifugation at 12 000 g and filtration through a 0.22 *μ*m filter. Each supernatant was adjusted to the appropriate concentration and transferred to an individual well of a 96-well microplate. Betalain content was estimated spectrophotometrically using a Varioskan Flash multimode microplate reader (Thermo Scientific, Waltham, USA) as $A_{540}-0.33A_{660}$, where $A_{540}$ and $A_{660}$ are the absorbance values for betacyanins and chlorophyll at 540 nm and 660 nm. Absorbance values were converted to betalain equivalents using the molar extinction coefficient $\varepsilon =60,000\;\text{l}\;\text{mo}{\text{l}}^{-1}\;\text{c}{\text{m}}^{-1}$ and molecular weight=550 Da [37].

## 3. Results

### 3.1. Quantitative Characterization of Diverse Promoters by a GFP Assay

Promoters have been widely characterized in various plant cells and have been used in plant biotechnology area [38–41]. However, their quantitative information about the promoter strength has not been fully investigated, which is helpful in order to introduce novel traits into a designed plant. To achieve this goal, nineteen promoter sequences (consisting of the upstream regulatory region, core promoter region, and 5${}^{\prime }$ untranslated region) (Table S1), most of which have been functionally tested [38–45], were selected for quantitative characterization on promoter strength. Each individual promoter was assembled with GFP and the Nos terminator sequence to form an expression cassette, resulting in a library of *Agrobacterium* binary vectors.

**Figure 1:**
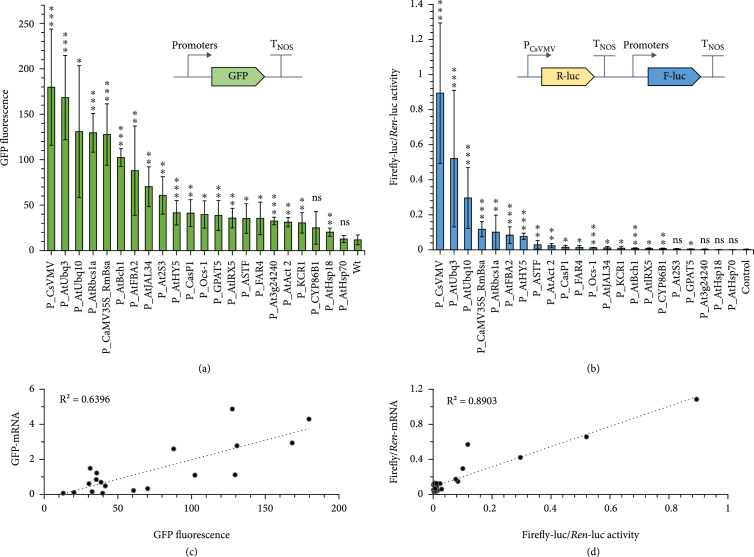
Comparison of a single GFP reporter system and a dual-luciferase reporter system in *N. benthamiana*. (a) Schematic diagram of constructs used for the single GFP reporter system: an individual promoter drives the expression of GFP with the assistance of the terminator T_nos. (b) Schematic diagram of constructs used for the dual-luciferase reporter system. *Renilla* luciferase (R-luc) is driven by the promoter P_CsVMV and the terminator T_nos, and firefly luciferase (F-luc) is driven by the individual tested promoter with the assistance of the terminator T_nos. The dual-luciferase activity is defined as the F-luc/R-luc luminescence intensity. (c) The correlation of GFP mRNA level and GFP protein level. (d) The correlation of firefly/*Renilla* mRNA level and F/R-luc activity. Data are presented as the mean±standard error of at least three independent replicates. P values were calculated using unpaired two-tailed Student’s t-test; ${}^{*}P\leq 0.05$, ${}^{**}P\leq 0.01$, and ${}^{***}P\leq 0.001$; ns = not significant.

Because of the feasibility of transient gene expression in *N. benthamiana* via *Agrobacterium tumefaciens-*mediated leaf infiltration, plasmids containing each promoter driving the expression of GFP were transiently transformed into 4-week-old tobacco leaves. The intensity of GFP fluorescence from the infiltrated leaves was captured using a previously described automated image collection system [46]. The constitutive promoter P_CsVMV displayed the highest transient expression level, followed by P_AtUbq3, P_AtUbq10, P_AtRBSa1, and P_CaMV35S (Figure 1(a)). Four promoters, P_AtBch1, P_AtFBA2, P_AtJAL34, and P_At2S3, showed relatively moderate fluorescence levels, and the remaining 13 promoters, including P_AtHY5, P_CasP1, and P_DAISY, had very low levels of detectable transient expression (Figure 1(a)).

To verify the reliability of the single GFP reporter system for plant promoter strength evaluation, mRNA levels were also measured to provide additional information on the expression of the reporter gene (Figure S2a). The mRNA levels were high under the control of strong promoters and relatively low under the control of weak promoters, with a correlation of 0.64 (P<0.001) between GFP mRNA abundance and fluorescence intensity (Figure 1(c)). The discrepancies were clearly observed when weak promoters were used.

### 3.2. Evaluation of Promoters Using a Dual-Luciferase Reporter System

The dual-luciferase reporter system has been reported to be highly sensitive and able to reduce background noise interference in plant cells [47]. To minimize the discrepancy between mRNA and protein expression, a dual-luciferase assay was developed for determining the relative strength of plant promoters. A constitutively expressed *Renilla* luciferase (R-luc) reporter gene (driven by P_CsVMV) was used as an internal normalization control for transformation efficiency, and each tested promoter was linked to firely luciferase (F-luc) to provide a quantitative readout normalized to the expression of P_CsVMV::R-luc::T_nos.

Consistent with the GFP-based assay above, our dual-luciferase reporter analysis showed that P_CsVMV, P_AtUbq3, and P_AtUbq10 produced high ratiometric R-luc/F-luc activities, indicating their strong transcriptional activities (Figure 1(b)). However, medium and weak promoters, such as P_CaMV35S_RmBsa, P_AtRbcs1a, P_AtFBA2, P_AtHY5, and P_ASTF, displayed different intensity rankings between the GFP and dual-luciferase assays. The mRNA profiles of firefly luciferase were also detected to verify the reliability of the dual-luciferase system (Figure S2(b)). The correlation between mRNA and reporter signal in the dual-luciferase-based assay ($R^{2}=0.89$, P<0.001) was much higher than that in the single GFP assay (Figure 1(d)), indicating that the dual-luciferase reporter system is highly reliable and accurate for quantitative characterization of plant promoters.

**Figure 2:**
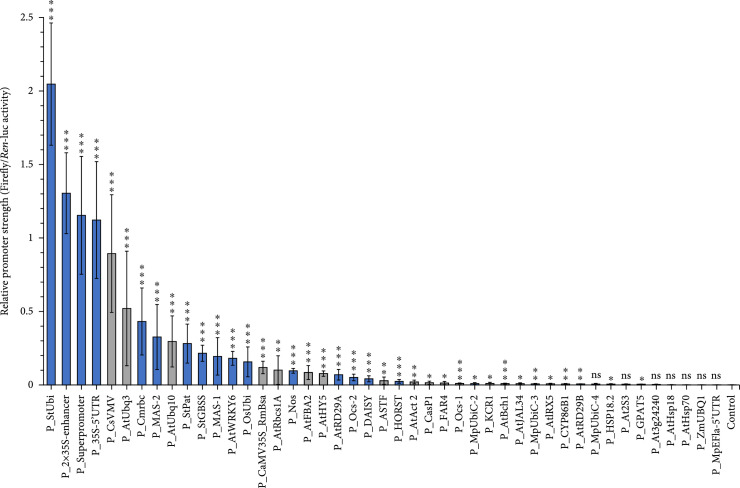
Individual promoters from the standard gene library show varying levels of dual-luciferase activity in *N. benthamiana*. Relative promoter strength was defined as the ratio of firefly luciferase to *Renilla* luciferase luminescence intensity. Data are presented as the mean±standard error of at least five independent experimental replicates. Grey columns represent the data shown in Figure 1. P values were calculated using unpaired two-tailed Student’s t-test; ${}^{*}P\leq 0.05$, ${}^{**}P\leq 0.01$, and ${}^{***}P\leq 0.001$; ns = not significant.

To systematically evaluate new promoter candidates, we constructed expression vectors for 25 additional promoters from five plant species, including *Arabidopsis*, *M. polymorpha*, *S. tuberosum*, *Z. mays*, and *O. sativa*, as well as virus sources (Figure 2; see Table S1 for details, including references and sequences information). Many of the most highly active genetic elements originated from viruses, whereas promoters from *M. polymorpha* (P_MpUbiC-4, P_MpUbiC-2, P_MpUbiC-3, and P_MpEFla-5${}^{\prime }$UTR) produced extremely low levels of reporter gene expression (Figure 2). The activity of the P_StUbi promoter from potato was 10 times higher than that of P_AtUbq10 or P_AtUbq3 from *A. thaliana* and even higher than that of the widely used constitutive promoter P_2×35S-enhancer (Figure 2).

### 3.3. Determination of the Best Time Point for Promoter Activity Measurement

**Figure 3:**
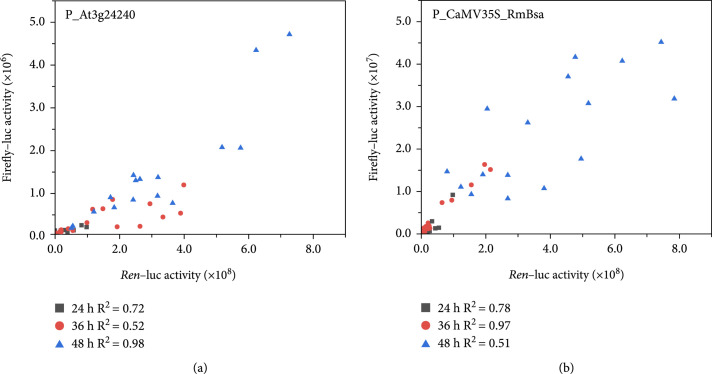
Time course measurement of firefly/*Renilla* luciferase activities. *Renilla* luciferase was driven by P_CsVMV and firefly luciferase was driven by P_At3g24240 (a) or by P_CaMV35S_RmBsa (b) at 24 h (grey squares), 36 h (red circles), and 48 h (blue triangles) after infiltration.

Promoters not only control the intensity of gene expression but also contribute to the timing and duration of protein accumulation. During promoter characterization by transient expression analysis, differences in the timing and intensity of GFP expression are commonly observed [21]. The dual-luciferase reporter system permits expression measurement at only a single time point, and different outputs are therefore obtained when different time points are selected for measurement. However, few studies have focused on time point selection in the context of protein production. The key time point has always been chosen empirically after 2–5 days of reporter expression in previous studies [24, 27, 34]. Here, to better determine a suitable sampling time for promoter intensity measurement, leaves transiently expressing luciferase were periodically sampled at 12 h intervals. In the beginning, we selected four promoters (P_AtFBA2, P_AtUbq10, P_AtUbq3, and P_CsVMV) and seven time points after agroinfiltration (24, 36, 48, 60, 72, 84, and 96 h) for F-luc and R-luc activity measurement (Figure S3). The dual-luciferase activity increased linearly in the first 60 h. After that time point, the dual-luciferase activity varied greatly, especially under the control of P_AtFBA2 and P_CsVMV. To confirm this sampling time result, activities driven by P_At3g24240 and P_CaMV35S_RmBsa were measured (Figure 3). The luciferase activities were extremely low during the first 24 h after agroinfiltration, probably because of the low protein dose. At 36 h and 48 h, there was a significant increase in activity due to continued protein expression. Based on the results from different sampling times, the activities of firefly and *Renilla* luciferase had a relatively consistent linear relationship. When linear regression was performed using data from different time points, the $R^{2}$ for firefly luciferase activity driven by P_At3g24240 versus *Renilla* luciferase activity driven by P_CsVMV was 0.72, 0.52, and 0.98 at 24, 36, and 48 h (Figure 3(a)). The $R^{2}$ for firefly luciferase activity driven by P_CaMV35S_RmBsa versus *Renilla* luciferase activity driven by P_CsVMV was 0.78, 0.97, and 0.51 (Figure 3(b)). For P_At3g24240 and P_CaMV35S_RmBsa, the $R^{2}$ was 0.76 and 0.83 (Figure S3). These results demonstrated that the F-luc/R-luc activity ratios at 36 h and 48 h represented the relative strengths of the tested promoters.

### 3.4. Evaluation of Promoters in Cultured BY2 Cells

Tobacco Bright Yellow-2 (BY2) cells are considered to be an effective chassis for the production of pharmaceutical and nutraceutical materials [14, 16]. However, there has not previously been a relevant, full characterization of heterogeneous plant-derived promoters in BY2 cells.

**Figure 4:**
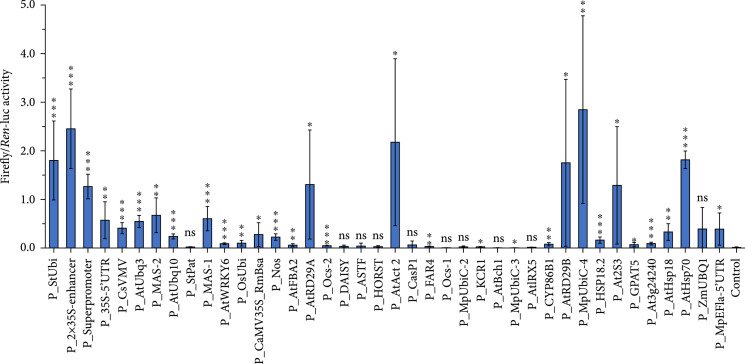
Different promoters from the standard gene library show varying levels of dual-luciferase activity in BY2. Relative promoter strength was defined as the ratio of firefly luciferase to *Renilla* luciferase luminescence intensity. Data are presented as the mean±standard error of at least five independent replicates. P values were calculated using unpaired two-tailed Student’s t-test; ${}^{*}P\leq 0.05$, ${}^{**}P\leq 0.01$, and ${}^{***}P\leq 0.001$; ns = not significant.

To expand the promoters available for BY2 cells, *Agrobacterium* harboring individual dual-luciferase constructs were cocultivated with BY2 cells. After 4 days of incubation, the transformed BY2 cells were used for the analysis of firefly/*Renilla* luciferase activities (Figure 4). Comparing with the promoter activities tested in *N. benthamiana*, most of these promoters performed relatively consistently between two types of tobacco cells. Only one promoter P_StPat gave a worse performance on gene expression in BY2 cells (Figure S4). Eight promoters, including the seed-specific promoter P_At2S3 and the root-specific promoter P_At3g24240, showed higher activities in BY2 cells than in tobacco leaves, probably because of differences in physiological conditions associated with cell differentiation. The dual-luciferase activity temperature-sensitive promoters, such as P_AtHsp18, P_AtHsp70, and P_AtRD29B, were markedly higher in BY2 cells (Figure 4).

### 3.5. Terminators Affect Gene Expression

**Figure 5:**
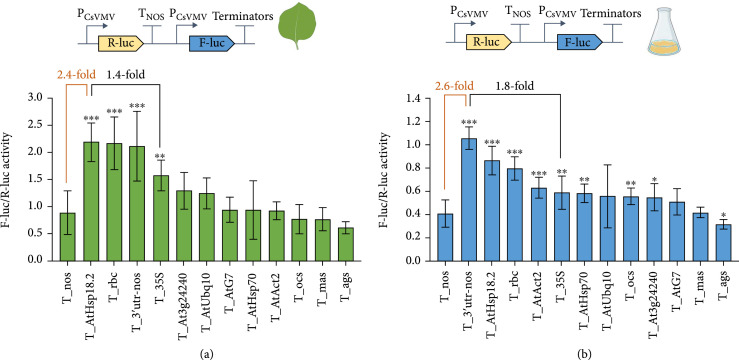
Evaluation of the effects of different terminators on fluc-luciferase activity in *N. benthamiana* leaves (a) and BY2 cells (b). Relative promoter strength was defined as the ratio of firefly luciferase (F-luc) luminescence intensity to *Renilla* luciferase (R-luc) luminescence intensity. Data are presented as the mean±standard error of five independent biological replicates. P values were calculated using unpaired two-tailed Student’s t-test; ${}^{*}P\leq 0.05$, ${}^{**}P\leq 0.01$, and ${}^{***}P\leq 0.001$; ns = not significant.

Terminators have been reported to influence the level of transgene expression in plants [48]. However, for a long time, the most widely used terminators in plant biotechnology have been limited to the nopaline synthase and octopine synthase terminators and the 35S terminator from cauliflower mosaic virus. Therefore, to expand the available terminators, 13 terminators from plant and viral sources were investigated for their effects on gene expression (Table S2). The constitutive promoter P_CsVMV and the terminator T_nos were used to express R-luc, which was set as the internal normalization control for transformation efficiency. P_CsVMV linked to F-luc and individual tested terminators was used to evaluate the influence of individual terminators on gene expression (Figure 5(a)).

These constructs were delivered to *N. benthamiana* leaves by agroinfiltration, and F-luc/R-luc activities were evaluated. T_AtHsp18.2 outperformed the frequently used T_35S and T_nos terminator, producing 1.4- and 2.4-fold increases in F-luc expression, respectively (Figure 5(a)). To determine whether the tested terminators performed similarly in BY2 cells, all constructs were infiltrated with fresh BY2 cells for 4 days. As in *N. benthamiana*, the best-performing terminator T_3${}^{\prime }$utr-nos produced 1.8- and 2.6-fold higher expression than the constitutive terminators T_35S and T_nos in BY2, respectively (Figure 5(b)). These results indicated that the terminators show generally consistent performance in transient expression in two different plant cell types.

We further evaluated the effects of a library of promoter/terminator combinations on gene expression. A total of 105 combinations were measured, and the results showed a wide range of expression levels with over a 326-fold difference between the best- (P_CsVMV/T_AtHsp18.2) and worst-performing combinations (P_AtRD29B/T_nos) (Figure S5). Thus, our research highlights the importance of selecting an appropriate promoter/terminator combination for optimal transgenic expression.

### 3.6. Modulation of the Betalain Biosynthetic Pathway in Tobacco Cells

**Figure 6:**
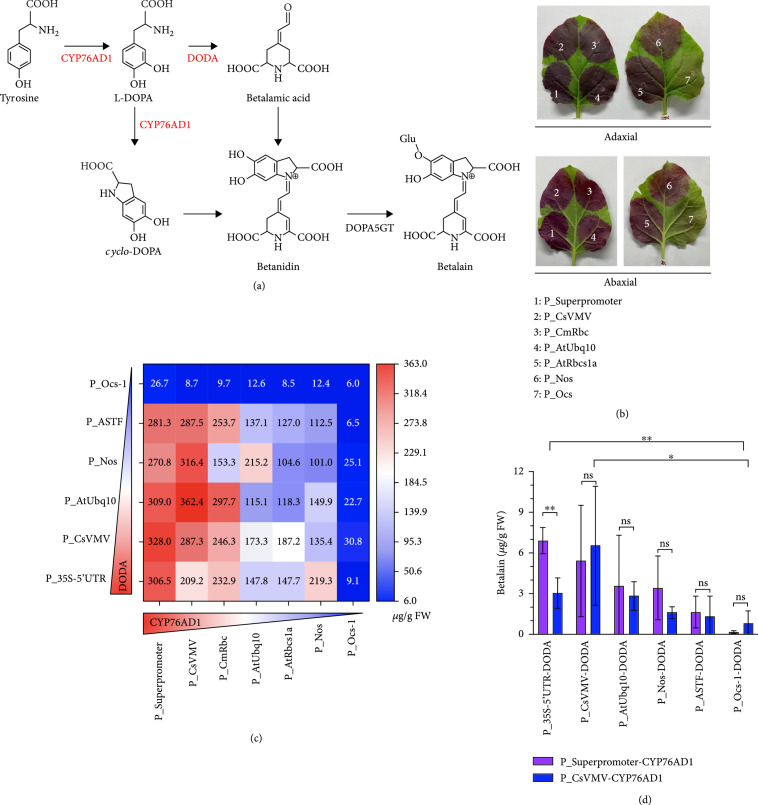
The betalain pathway was engineered into tobacco by promoter selection. (a) The betalain biosynthetic pathway. DODA: DOPA 4,5-dioxygenase; DOPA5GT: cyclo-DOPA-5-O-glucosyltransferase. (b) Leaf color phenotypes of *N. benthamiana* coinfiltrated with *Agrobacterium* harboring plasmids for the expression of CYP76AD1, DODA, and DOPA5GT. Numbers indicate CYP76AD1 was under the control of the corresponding promoters and T_nos. DODA was controlled by P_CsVMV and T_nos. DOPA5GT was controlled by P_35S-5${}^{\prime }$UTR and T_nos. The adaxial (upper) and abaxial (lower) sides of the leaves are presented. (c) The production of betalain in *N. benthamiana* leaves. (d) The production of betalain in cultured BY2 cells. Data are presented as the mean±standard error of five independent biological replicates. P values were calculated using unpaired two-tailed Student’s t-test; ${}^{*}P\leq 0.05$, ${}^{**}P\leq 0.01$, and ${}^{***}P\leq 0.001$; ns = not significant.

An appropriate selection of regulatory elements can achieve different expression goals in metabolic regulation. After characterization, regulatory elements were used for the metabolic synthesis of betalain. Betalains are a group of reddish pigments found in some fruits and have been used to visualize gene expression in plants [49]. Here, we chose betalain as the target of regulatory elements to explore the influence of promoters on metabolic regulation (Figure 6(a)).

Seven promoters with different strengths were used to modulate the key enzymes of the betalain synthetic pathway, CYP76AD1 and DODA (DOPA-4,5-dioxygenase), to various levels (Figure S6). When DOPA5GT (cyclo-DOPA-5-O-glucosyltransferase) was driven by P_35S-5${}^{\prime }$UTR, the betalain yield was consistent with the strengths of the other promoters. A high yield of betalain produced a dark red color after transient expression in leaves, whereas a faint red color was associated with relatively low yields (Figure 6(b)). Betalain yield was higher when CYP76AD1 was driven by P_Superpromoter or P_CsVMN and DODA was driven by P_35S-5${}^{\prime }$UTR or P_CsVNV compared with the weak promoters P_NOS/P_Ocs-1(Figure 6(c)). However, the best yield performance was not simply obtained when all of the synthetic genes were expressed at the highest levels. In fact, the highest yield (362.4 *μ*g/g fresh weight (FW)) was obtained with P_CsVMV::CYP76AD1 and P_AtUbq10::DODA, almost 60-fold higher than that obtained with P_Ocs-1::CYP76AD1 and P_Ocs-1::DODA (6.0 *μ*g/g FW).

We next used the diverse constructs to produce betalain in BY2 cells (Figure 6(d) and Figure S7). The results were generally consistent with those in *N. benthamiana* leaves: higher levels of protein expression resulted in higher yields. However, the betalain yield was extremely low in BY2 cells, perhaps because of low levels of precursor accumulation or low transient transformation efficiency (Figure S7).

## 4. Discussion

In higher plants, the *GUS* gene has frequently been used as a reporter gene for promoter analysis [27, 28]. However, its available substrates are membrane impermeable, and it has not generally been accepted as a quantitative reporter for gene expression *in vivo*. Nonetheless, the *GFP* gene has been used extensively for detecting gene expression, especially as a tool to visualize spatial and temporal patterns of gene expression *in vivo* [29] and to study intracellular protein localization or protein-protein interactions [50]. Many studies have shown that GFP is useful as a quantitative reporter of gene expression in *E. coli* [51], and a few studies have reported its use for quantitative promoter characterization in plants [20, 52]. We therefore used GFP for our initial tests. The performance of promoters with strong activity could be clearly distinguished from that of promoters with medium and weak activities based on the intensity of the green fluorescence signal (Figure 1(a)). However, there were no discernible differences among promoters with weak activity. The correlation of fluorescence intensity with GFP mRNA was found to be 0.64, underscoring the variability of this method. One of the main reasons for this variability is that background fluorescence and chlorophyll interference with GFP detection are unavoidable [21]. Other disadvantages include the inconsistency in GFP expression among different plant species [53] and the fact that green fluorescence intensity tended to decline after fluorescence emergence (data not shown), which led to variability in quantitative promoter outputs [21].

The variability commonly observed among samples and experiments can be reduced by using ratiometric assays with a second invariable reporter as an internal reference. Recently, a ratiometric dual-color luciferase reporter assay with green- and red-emitting luciferases was developed to quantify the transcription of genetic elements in plants [30]. Although these assays do not require protein extraction and their results can be directly detected *in vivo*, the interference of chlorophyll still remains. In addition, the partial signal overlap between green-luc and red-luc makes it difficult to precisely evaluate the genetic parts. Compared with the dual-color luciferase system, the firefly/*Renilla* luciferase assay, based on a chemiluminescence reaction, is more sensitive and is particularly useful for monitoring the expression of multiple genes by chemiluminescence detection. The chemiluminescence reaction uses unique substrates for the differentiation of F-luc and R-luc, and interference in plant cells can thus be markedly reduced. Here, the correlation of F-luc/R-luc activity with F-luc mRNA was found to be 0.89, demonstrating the reliability of this dual-luciferase system for precise promoter characterization.

Fifty-eight genetic parts (45 promoters and 13 terminators) from six plant species (*Marchantia*, the monocots maize and rice, and the dicots *Arabidopsis*, *S. tuberosum*, and *C. morifolium*), viruses, and *Agrobacterium* were assessed in the present research. Their abilities to drive transcription were measured in two tobacco systems (*N. benthamiana* leaves and BY2 cultured cells). In general, we can conclude that (1) dicot promoters tended to perform better than monocot promoters in the dicot tobacco system, (2) promoters and terminators performed relatively consistently between the two types of tobacco cells, and (3) *Marchantia* promoters showed extremely low activity in the tobacco system. These conclusions suggest that it is not a good choice to use a dicot promoter in a monocot plant system or to use a monocot promoter for engineering in a dicot chassis.

As mentioned above, quantitative characterization of our promoter library was performed in two chassis with a transient expression system. DNA containing the promoter and reporter gene sequences was introduced into the plant cells, and the observed rapid expression probably resulted from the extrachromosomal activity of the introduced genes. As an extrachromosomal entity, the expression of the introduced cassette is not influenced by hereditary effects such as chromosome structure or the insertion site of a foreign gene, enabling promoter strength to be quantitatively standardized. However, DNA transfer efficiency may show variability, depending on the method of DNA transfer and the recipient plant species, thus leading to inconsistencies between the results of transient expression and stable transformation. Some inconsistencies were still present between the two plant chassis selected for measurement. For example, the activities of P_MpUbi-4, P_AtAct2, P_AtRD29B, and P_AtHsp70 were much higher in BY2 cells than in *N. benthamiana*. Although the dual-luciferase reporter system uses the constitutive expression of firefly luciferase as an internal normalization control for transformation efficiency, variability of the outputs cannot be completely avoided.

Several limiting factors were present in this study and should be further considered in the future research. First, the transient expression system is sensitive to gene silencing of highly expressed genes [54], and the P19 protein was therefore coexpressed with the reporter gene to repress gene silencing in our constructed vectors. However, a number of publications have demonstrated that P19 can enhance the expression of several diverse proteins, including GFP [55, 56]. Therefore, the effect of P19 on reporter gene expression in the present evaluation system should be considered further. Second, most of the tested promoters, especially the tissue-specific and inducible promoters, contain diverse cis-regulatory elements, some of which are putative and have not been fully biologically characterized. The performance of individual promoters can therefore be regulated by native transcription factors, as well as abiotic and biotic stress. Hence, the performance of individual promoters may change in response to plant growth conditions and developmental stages. Third, the UTR (untranslated regions) of a promoter or terminator can enhance gene translation or mRNA stability [57, 58]. It is likely that the performance of hybrid sequences (different 5${}^{\prime }$/3${}^{\prime }$UTR with a promoter/terminator) varies with the length and source of the introduced genes.

Plant scientists are currently working to effectively deliver complex traits into plants, including plant metabolic pathways, synthetic switches, and regulatory circuits. The design and characterization of the genetic parts of promoters and terminators are key stages in the design/build/test/learn cycle. However, the complex genetic makeup and relatively long-life cycle of plants necessitate the use of iterative rounds of testing and modification, which are cumbersome. Thus, design-led rational engineering is particularly important for developing the best solution. Quantitative measurement of genetic parts provides parameters for the construction of mathematical models that can predict their behavior before implementation in plants, and this approach should become mainstream in plant synthetic biology.

In this work, we used two transient expression platforms (*N. benthamiana* leaves and BY2 suspension cells) to rapidly screen the activities of promoters and terminators. We used a dual-luminescence reporter system to quantitatively evaluate the performance of a library of 58 plant-based genetic parts. As a proof of concept, we engineered the betalain metabolic pathway into *N. benthamiana* using well-established promoters to control the expression levels of the bottleneck enzymes CYP76AD1 and DOPA, and the final yield reached 362.4 μg/g FW. Our systematic approach not only demonstrates the different intensities of multiple promoter sequences in *N. benthamiana* and BY2 cells but also adds to the toolbox of plant promoters for plant engineering. This work highlights the potential application of well-established promoters for the modulation of metabolic pathways through the precise control of gene expression.

## Data Availability

Raw data used to support the findings presented in this study are available from the corresponding author upon request.

## Supplementary Materials

Supplementary Materials Figure S1: type IIS assembly strategy for rapid plasmid construction. Figure S2: mRNA levels of reporter genes driven by different promoters. Figure S3: dual-luciferase activity measurement with four representative promoters at different sampling times. Figure S4: comparison of relative transient expression levels of individual promoters in BY2 cells and N. benthamiana leaves. Figure S5: expression cassette strength library obtained by integration of terminator elements. Figure S6: different strengths of promoters used in betalain synthesis. Figure S7: BY2 cells transformed with betalain synthesis genes. Table S1: detailed information on the promoters in this study. Table S2: detailed information on the terminators used in this study. Table S3: primers used in this study.

## References

[1] F. J. Molina-Hidalgo, M. Vazquez-Vilar, L. D'Andrea, O. C. Demurtas, P. Fraser, G. Giuliano, R. Bock, D. Orzáez, and A. Goossens, “Engineering metabolism in Nicotiana species: a promising future,” *Trends in Biotechnology*, vol. 39, no. 9, pp. 901–913, 2021 33341279 10.1016/j.tibtech.2020.11.012

[2] C. R. Boehm, and R. Bock, “Recent advances and current challenges in synthetic biology of the plastid genetic system and metabolism,” *Plant Physiology*, vol. 179, no. 3, pp. 794–802, 2019 30181342 10.1104/pp.18.00767 PMC6393795

[3] V. J. Martin, D. J. Pitera, S. T. Withers, J. D. Newman, and J. D. Keasling, “Engineering a mevalonate pathway in Escherichia coli for production of terpenoids,” *Nature Biotechnology*, vol. 21, no. 7, pp. 796–802, 2003 10.1038/nbt833 12778056

[4] S. Galanie, K. Thodey, I. J. Trenchard, M. Filsinger Interrante, and C. D. Smolke, “Complete biosynthesis of opioids in yeast,” *Science*, vol. 349, no. 6252, pp. 1095–1100, 2015 26272907 10.1126/science.aac9373 PMC4924617

[5] J. Li, C. Tian, Y. Xia, I. Mutanda, K. Wang, and Y. Wang, “Production of plant-specific flavones baicalein and scutellarein in an engineered E. coli from available phenylalanine and tyrosine,” *Metabolic Engineering*, vol. 52, pp. 124–133, 2019 30496827 10.1016/j.ymben.2018.11.008

[6] S. Wu, M. Schalk, A. Clark, R. B. Miles, R. Coates, and J. Chappell, “Redirection of cytosolic or plastidic isoprenoid precursors elevates terpene production in plants,” *Nature Biotechnology*, vol. 24, no. 11, pp. 1441–1447, 2006 10.1038/nbt1251 17057703

[7] K. Zhou, K. J. Qiao, S. Edgar, and G. Stephanopoulos, “Distributing a metabolic pathway among a microbial consortium enhances production of natural products,” *Nature Biotechnology*, vol. 33, no. 4, pp. 377–383, 2015 10.1038/nbt.3095 PMC4867547 25558867

[8] E. Fesenko, and R. Edwards, “Plant synthetic biology: a new platform for industrial biotechnology,” *Journal of Experimental Botany*, vol. 65, no. 8, pp. 1927–1937, 2014 24638901 10.1093/jxb/eru070

[9] W. Liu, and C. N. Stewart Jr., “Plant synthetic biology,” *Trends in Plant Science*, vol. 20, no. 5, pp. 309–317, 2015 25825364 10.1016/j.tplants.2015.02.004

[10] B. Diego-Martin, B. González, M. Vazquez-Vilar, S. Selma, R. Mateos-Fernández, S. Gianoglio, A. Fernández-del-Carmen, and D. Orzáez, “Pilot production of SARS-CoV-2 related proteins in plants: a proof of concept for rapid repurposing of indoor farms into biomanufacturing facilities,” *Frontiers in Plant Science*, vol. 11, article 612781, 2020 10.3389/fpls.2020.612781 PMC7785703 33424908

[11] P. Beyer, S. Al-Babili, X. Ye, P. Lucca, P. Schaub, R. Welsch, and I. Potrykus, “Golden rice: introducing the *β*-carotene biosynthesis pathway into rice endosperm by genetic engineering to defeat vitamin A deficiency,” *Journal of Nutrition*, vol. 132, no. 3, pp. 506S–510S, 2002 11880581 10.1093/jn/132.3.506S

[12] D. Breitel, P. Brett, S. Alseekh, A. R. Fernie, E. Butelli, and C. Martin, “Metabolic engineering of tomato fruit enriched in L-DOPA,” *Metabolic Engineering*, vol. 65, pp. 185–196, 2021 33242649 10.1016/j.ymben.2020.11.011 PMC8054910

[13] C. N. Stewart, N. Patron, A. D. Hanson, and J. M. Jez, “Plant metabolic engineering in the synthetic biology era: plant chassis selection,” *Plant Cell Reports*, vol. 37, no. 10, pp. 1357–1358, 2018 30196331 10.1007/s00299-018-2342-1

[14] Y. Tekoah, A. Shulman, T. Kizhner, I. Ruderfer, L. Fux, Y. Nataf, D. Bartfeld, T. Ariel, S. Gingis-Velitski, U. Hanania, and Y. Shaaltiel, “Large-scale production of pharmaceutical proteins in plant cell culture-the protalix experience,” *Plant Biotechnology Journal*, vol. 13, no. 8, pp. 1199–1208, 2015 26102075 10.1111/pbi.12428

[15] S. Hellwig, J. Drossard, R. M. Twyman, and R. Fischer, “Plant cell cultures for the production of recombinant proteins,” *Nature Biotechnology*, vol. 22, no. 11, pp. 1415–1422, 2004 10.1038/nbt1027 15529167

[16] T. Imamura, N. Isozumi, Y. Higashimura, S. Ohki, and M. Mori, “Production of ORF8 protein from SARS-CoV-2 using an inducible virus-mediated expression system in suspension-cultured tobacco BY-2 cells,” *Plant Cell Reports*, vol. 40, no. 3, pp. 433–436, 2021 33399879 10.1007/s00299-020-02654-5 PMC7783501

[17] C. M. Hernandez-Garcia, and J. J. Finer, “Identification and validation of promoters and cis-acting regulatory elements,” *Plant Science*, vol. 217-218, pp. 109–119, 2014 24467902 10.1016/j.plantsci.2013.12.007

[18] W. Hu, and C. L. Cheng, “Expression of Aequorea green fluorescent protein in plant cells,” *FEBS Letters*, vol. 369, no. 2-3, pp. 331–334, 1995 7649282 10.1016/0014-5793(95)00776-6

[19] M. Axelos, C. Curie, and L. Mazzolini, “A protocol for transient gene expression in Arabidopsis thaliana protoplasts isolated from cell suspension cultures,” *Plant Physiology and Biochemistry*, vol. 30, no. 1, pp. 123–128, 1992

[20] C. Engler, M. Youles, R. Gruetzner, T. M. Ehnert, S. Werner, J. D. G. Jones, N. J. Patron, and S. Marillonnet, “A golden gate modular cloning toolbox for plants,” *ACS Synthetic Biology*, vol. 3, no. 11, pp. 839–843, 2014 24933124 10.1021/sb4001504

[21] A. Gunadi, P. J. Rushton, L. K. McHale, A. H. Gutek, and J. J. Finer, “Characterization of 40 soybean (Glycine max) promoters, isolated from across 5 thematic gene groups,” *Plant Cell, Tissue and Organ Culture*, vol. 127, no. 1, pp. 145–160, 2016

[22] T. W. J. M. van Herpen, K. Cankar, M. Nogueira, D. Bosch, H. J. Bouwmeester, and J. Beekwilder, “Nicotiana benthamiana as a production platform for artemisinin precursors,” *PLoS One*, vol. 5, no. 12, 2010 10.1371/journal.pone.0014222 PMC2997059 21151979

[23] R. S. Nett, W. Lau, and E. S. Sattely, “Discovery and engineering of colchicine alkaloid biosynthesis,” *Nature*, vol. 584, no. 7819, pp. 148–153, 2020 32699417 10.1038/s41586-020-2546-8 PMC7958869

[24] J. Li, I. Mutanda, K. Wang, L. Yang, J. Wang, and Y. Wang, “Chloroplastic metabolic engineering coupled with isoprenoid pool enhancement for committed taxanes biosynthesis in Nicotiana benthamiana,” *Nature Communications*, vol. 10, no. 1, p. 4850, 2019 10.1038/s41467-019-12879-y PMC6813417 31649252

[25] P. K. Ajikumar, W. H. Xiao, K. E. Tyo, Y. Wang, F. Simeon, E. Leonard, O. Mucha, T. H. Phon, B. Pfeifer, and G. Stephanopoulos, “Isoprenoid pathway optimization for Taxol precursor overproduction in Escherichia coli,” *Science*, vol. 330, no. 6000, pp. 70–74, 2010 20929806 10.1126/science.1191652 PMC3034138

[26] C. De Wilde, H. Van Houdt, S. De Buck, G. Angenon, G. De Jaeger, and A. Depicker, “Plants as bioreactors for protein production: avoiding the problem of transgene silencing,” *Plant Molecular Biology*, vol. 43, no. 2/3, pp. 347–359, 2000 10999415 10.1023/a:1006464304199

[27] B. Wang, A. B. Kashkooli, A. Sallets, H. M. Ting, N. C. A. de Ruijter, L. Olofsson, P. Brodelius, M. Pottier, M. Boutry, H. Bouwmeester, and A. R. van der Krol, “Transient production of artemisinin in Nicotiana benthamiana is boosted by a specific lipid transfer protein from A. Annua,” *Metabolic Engineering*, vol. 38, pp. 159–169, 2016 27421621 10.1016/j.ymben.2016.07.004

[28] Y. M. Cai, K. Kallam, H. Tidd, G. Gendarini, A. Salzman, and N. J. Patron, “Rational design of minimal synthetic promoters for plants,” *Nucleic Acids Research*, vol. 48, no. 21, pp. 11845–11856, 2020 32856047 10.1093/nar/gkaa682 PMC7708054

[29] M. S. Belcher, K. M. Vuu, A. Zhou, N. Mansoori, A. Agosto Ramos, M. G. Thompson, H. V. Scheller, D. Loqué, and P. M. Shih, “Design of orthogonal regulatory systems for modulating gene expression in plants,” *Nature Chemical Biology*, vol. 16, no. 8, pp. 857–865, 2020 32424304 10.1038/s41589-020-0547-4

[30] Y. Yang, J. H. Lee, M. R. Poindexter, Y. Shao, W. Liu, S. C. Lenaghan, A. H. Ahkami, E. Blumwald, and C. N. Stewart Jr., “Rational design and testing of abiotic stress-inducible synthetic promoters from poplar cis-regulatory elements,” *Plant Biotechnology Journal*, vol. 19, no. 7, pp. 1354–1369, 2021 33471413 10.1111/pbi.13550 PMC8313130

[31] R. Wu, L. Duan, J. L. Pruneda-Paz, D. H. Oh, M. Pound, S. Kay, and J. R. Dinneny, “The6xABRESynthetic promoter enables the spatiotemporal analysis of ABA-mediated transcriptional regulation,” *Plant Physiology*, vol. 177, no. 4, pp. 1650–1665, 2018 29884679 10.1104/pp.18.00401 PMC6084650

[32] E. González-Grandío, G. S. Demirer, W. Ma, S. Brady, and M. P. Landry, “A ratiometric dual color luciferase reporter for fast characterization of transcriptional regulatory elements in plants,” *ACS Synthetic Biology*, vol. 10, no. 10, pp. 2763–2766, 2021 34520169 10.1021/acssynbio.1c00248 PMC10503406

[33] K. A. Schaumberg, M. S. Antunes, T. K. Kassaw, W. Xu, C. S. Zalewski, J. I. Medford, and A. Prasad, “Quantitative characterization of genetic parts and circuits for plant synthetic biology,” *Nature Methods*, vol. 13, no. 1, pp. 94–100, 2016 26569598 10.1038/nmeth.3659

[34] A. Sarrion-Perdigones, M. Vazquez-Vilar, J. Palaci, B. Castelijns, J. Forment, P. Ziarsolo, J. Blanca, A. Granell, and D. Orzaez, “GoldenBraid 2.0: a comprehensive DNA assembly framework for plant synthetic biology,” *Plant Physiology*, vol. 162, no. 3, pp. 1618–1631, 2013 23669743 10.1104/pp.113.217661 PMC3707536

[35] M. D. Halfhill, R. J. Millwood, T. W. Rufty, A. K. Weissinger, and C. N. Stewart Jr., “Spatial and temporal patterns of green fluorescent protein (GFP) fluorescence during leaf canopy development in transgenic oilseed rape, Brassica napus L,” *Plant Cell Reports*, vol. 22, no. 5, pp. 338–343, 2003 14648109 10.1007/s00299-003-0696-4

[36] J. Schindelin, I. Arganda-Carreras, E. Frise, V. Kaynig, M. Longair, T. Pietzsch, S. Preibisch, C. Rueden, S. Saalfeld, B. Schmid, J. Y. Tinevez, D. J. White, V. Hartenstein, K. Eliceiri, P. Tomancak, and A. Cardona, “Fiji: an open-source platform for biological-image analysis,” *Nature Methods*, vol. 9, no. 7, pp. 676–682, 2012 22743772 10.1038/nmeth.2019 PMC3855844

[37] Y. Cai, and H. Corke, “Amaranthus betacyanin pigments applied in model food systems,” *Journal of Food Science*, vol. 64, no. 5, pp. 869–873, 1999

[38] S. Bihmidine, J. Lin, J. M. Stone, T. Awada, J. E. Specht, and T. E. Clemente, “Activity of the Arabidopsis RD29A and RD29B promoter elements in soybean under water stress,” *Planta*, vol. 237, no. 1, pp. 55–64, 2013 22983672 10.1007/s00425-012-1740-9

[39] K. M. J. Butaye, I. J. W. M. Goderis, P. F. J. Wouters, J. M. T. G. Pues, S. L. Delauré, W. F. Broekaert, A. Depicker, B. P. A. Cammue, and M. F. C. de Bolle, “Stable high-level transgene expression in Arabidopsis thaliana using gene silencing mutants and matrix attachment regions,” *Plant Journal*, vol. 39, no. 3, pp. 440–449, 2004 10.1111/j.1365-313X.2004.02144.x 15255872

[40] S. M. Chung, E. L. Frankman, and T. Tzfira, “A versatile vector system for multiple gene expression in plants,” *Trends in Plant Science*, vol. 10, no. 8, pp. 357–361, 2005 15993643 10.1016/j.tplants.2005.06.001

[41] S. Emami, M. C. Yee, and J. R. Dinneny, “A robust family of Golden Gate Agrobacterium vectors for plant synthetic biology,” *Frontiers in Plant Science*, vol. 4, p. 339, 2013 24032037 10.3389/fpls.2013.00339 PMC3759027

[42] V. M. Gondolf, R. Stoppel, B. Ebert, C. Rautengarten, A. J. M. Liwanag, D. Loqué, and H. V. Scheller, “A gene stacking approach leads to engineered plants with highly increased galactan levels in Arabidopsis,” *BMC Plant Biology*, vol. 14, no. 1, p. 344, 2014 25492673 10.1186/s12870-014-0344-x PMC4268804

[43] D. Miroshnichenko, A. Firsov, V. Timerbaev, O. Kozlov, A. Klementyeva, L. Shaloiko, and S. Dolgov, “Evaluation of plant-derived promoters for constitutive and tissue-specific gene expression in potato,” *Plants*, vol. 9, no. 11, p. 1520, 2020 33182387 10.3390/plants9111520 PMC7696379

[44] M. Ni, D. Cui, J. Einstein, S. Narasimhulu, C. E. Vergara, and S. B. Gelvin, “Strength and tissue-specificity of chimeric promoters derived from the octopine and mannopine synthase genes,” *Plant Journal*, vol. 7, no. 4, pp. 661–676, 1995

[45] T. Takahashi, and Y. Komeda, “Characterization of two genes encoding small heat-shock proteins in Arabidopsis thaliana,” *Molecular & General Genetics*, vol. 219, no. 3, pp. 365–372, 1989 2482931 10.1007/BF00259608

[46] M. T. Buenrostro-Nava, P. P. Ling, and J. J. Finer, “Comparative analysis of 35S and lectin promoters in transgenic soybean tissue using an automated image acquisition system and image analysis,” *Plant Cell Reports*, vol. 25, no. 9, pp. 920–926, 2006 16609890 10.1007/s00299-006-0142-5

[47] N. Matsuo, M. Minami, T. Maeda, and K. Hiratsuka, “Dual luciferase assay for monitoring transient gene expression in higher plants,” *Plant Biotechnology*, vol. 18, no. 1, pp. 71–75, 2001

[48] Y. J. Chen, P. Liu, A. A. Nielsen, J. A. Brophy, K. Clancy, T. Peterson, and C. A. Voigt, “Characterization of 582 natural and synthetic terminators and quantification of their design constraints,” *Nature Methods*, vol. 10, no. 7, pp. 659–664, 2013 23727987 10.1038/nmeth.2515

[49] Y. He, T. Zhang, H. Sun, H. Zhan, and Y. Zhao, “A reporter for noninvasively monitoring gene expression and plant transformation,” *Horticulture Research*, vol. 7, no. 1, p. 152, 2020 33024566 10.1038/s41438-020-00390-1 PMC7502077

[50] R. Rizzuto, M. Brini, P. Pizzo, M. Murgia, and T. Pozzan, “Chimeric green fluorescent protein as a tool for visualizing subcellular organelles in living cells,” *Current Biology*, vol. 5, no. 6, pp. 635–642, 1995 7552174 10.1016/s0960-9822(95)00128-x

[51] J. L. Lissemore, J. T. Jankowski, C. B. Thomas, D. P. Mascotti, and P. L. deHaseth, “Green fluorescent protein as a quantitative reporter of relative promoter activity in E. coli,” *BioTechniques*, vol. 28, no. 1, pp. 82–89, 2000 10649775 10.2144/00281st02

[52] T. Jores, J. Tonnies, T. Wrightsman, E. S. Buckler, J. T. Cuperus, S. Fields, and C. Queitsch, “Synthetic promoter designs enabled by a comprehensive analysis of plant core promoters,” *Nature Plants*, vol. 7, no. 6, pp. 842–855, 2021 34083762 10.1038/s41477-021-00932-y PMC10246763

[53] B. K. Harper, and C. N. Stewart, “Patterns of green fluorescent protein expression in transgenic plants,” *Plant Molecular Biology Reporter*, vol. 18, no. 2, pp. 141–141, 2000

[54] T. Dhillon, J. M. Chiera, and J. J. Finer, “Quantitative evaluation of six different viral suppressors of silencing using image analysis of transient GFP expression,” *Plant Cell Reports*, vol. 28, no. 4, pp. 639–647, 2009 19198843 10.1007/s00299-009-0675-5

[55] J. Win, and S. Kamoun *pCB301-p19: a binary plasmid vector to enhance transient expression of transgenes by agroinfiltration*, 2004, http://www.KamounLab.net.

[56] F. Garabagi, E. Gilbert, A. Loos, M. D. Mclean, and J. C. Hall, “Utility of the P19 suppressor of gene-silencing protein for production of therapeutic antibodies in *Nicotiana* expression hosts,” *Plant Biotechnology Journal*, vol. 10, no. 9, pp. 1118–1128, 2012 22984968 10.1111/j.1467-7652.2012.00742.x

[57] K. Leppek, R. Das, and M. Barna, “Functional 5' UTR mRNA structures in eukaryotic translation regulation and how to find them,” *Nature Reviews Molecular Cell Biology*, vol. 19, no. 3, pp. 158–174, 2018 29165424 10.1038/nrm.2017.103 PMC5820134

[58] S. K. Woo, R. P. Joshua, and B. Eric, “Tuning of mRNA stability through altering 3’-UTR sequences generates distinct output expression in a synthetic circuit driven by p53 oscillations,” *Scientific Reports*, vol. 9, no. 1, p. 5976, 2019 30979970 10.1038/s41598-019-42509-y PMC6461691

