# Supplementary material for: Benchmarking Intrinsic Promoters and Terminators for Plant Synthetic Biology Research
Source: Biodes Res. 2022 May 26;2022:9834989. doi: 10.34133/2022/9834989 (PMC10521690; doi:10.34133/2022/9834989)
Supplement: Supplementary Materials — Figure S1: type IIS assembly strategy for rapid plasmid construction. Figure S2: mRNA levels of reporter genes driven by different promoters. Figure S3: dual-luciferase activity measurement with four representative promoters at different sampling times. Figure S4: comparison of relative transient expression levels of individual promoters in BY2 cells and N. benthamiana leaves. Figure S5: expression cassette strength library obtained by integration of terminator elements. Figure S6: different strengths of promoters used in betalain synthesis. Figure S7: BY2 cells transformed with betalain synthesis genes. Table S1: detailed information on the promoters in this study. Table S2: detailed information on the terminators used in this study. Table S3: primers used in this study. [file 9834989.f1.pdf]

# Supplementary materials for

## Benchmarking intrinsic promoters and terminators for plant synthetic biology research

Chenfei Tian[1, 2], Yixin Zhang[3], Jianhua Li[1]*, Yong Wang[1]*

*[1] CAS-Key Laboratory of Synthetic Biology, CAS Center for Excellence in Molecular Plant Sciences, Institute of Plant Physiology and Ecology, Chinese Academy of Sciences, Shanghai 200032, China*

*[2] University of Chinese Academy of Sciences, Beijing 100039, China*

*[3] College of Life Science, Jilin Agricultural University, Changchun 130118, PR China*

* Corresponding author

Email address: yongwang@cemps.ac.cn (Y. Wang); jhli@cemps.ac.cn (J. Li).

Tel/Fax: 86-21-54924295

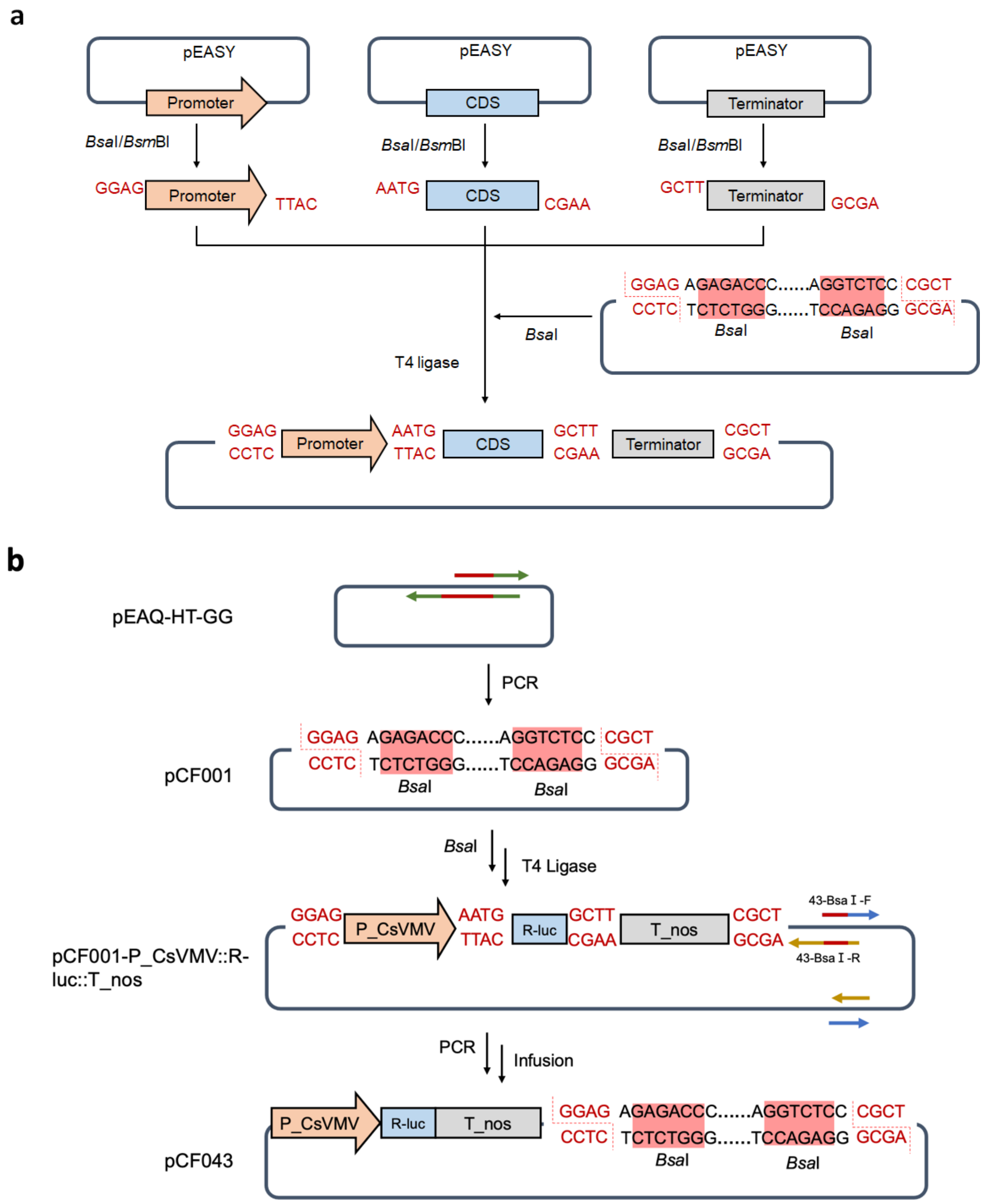


FIGURE S1. Type IIS assembly strategy for rapid plasmid construction. (a) Schematic diagram of plasmid construction. Standard genetic parts were inserted into the pEASY vector (TRANSGEN) with a standard restriction site. After digestion with *Bsa*I/*Bsm*BI, the genetic part segments with standardized 4-bp sticky ends could be sequentially linked by T4 ligase. The recognition sites of the Type II restriction enzymes *Bsa*I/*Bsm*BI were designed on the outside of both ends of the target fragment, so that the fragments recovered after digestion did not have the recognition site for digestion.

(b) GG3-*Bsa*I-F/GG3-*Bsa*I-R was used to PCR-amplify the pEAQ-HT-GG plasmid, adding the standard *Bsa*I restriction sites with GGAG and CGCT 4-bp overhangs to pEAQ-HT-GG to obtain the basic vector pCF001. For dual-luciferase assays, the contrasting reporter gene REN was expressed under the P_CsVMV promoter and the T_nos terminator, and this cassette was constructed according to the method in (a). Two pairs of primers were used to PCR-amplify the pCF001-P_CsVMV::REN-luc::T_nos plasmid to append the standard *Bsa*I restriction sites and obtain the vector pCF043 for dual-luciferase plasmid construction. The primers used for plasmid construction are listed in Supplementary Table 1.

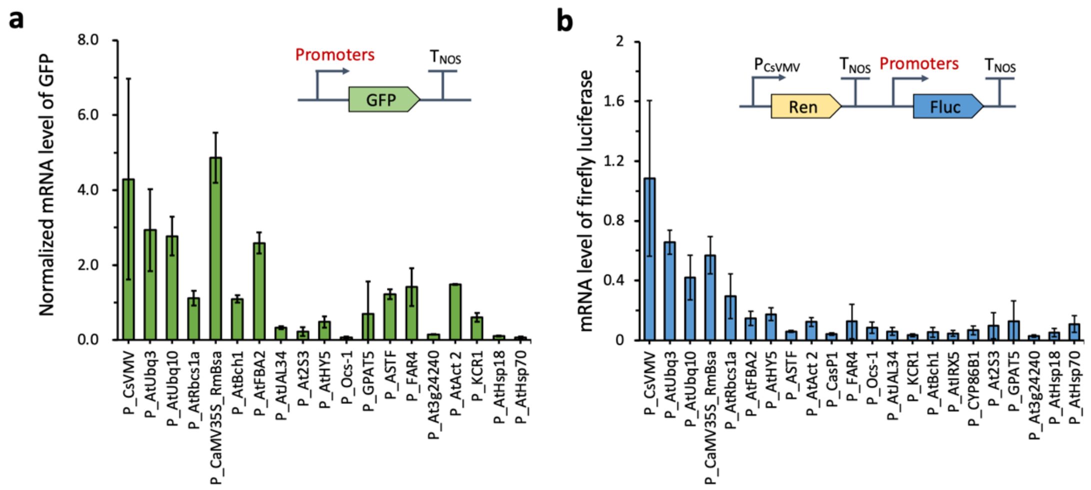


FIGURE S2. mRNA levels of reporter genes driven by different promoters. (a) normalized mRNA levels of GFP driven by different promoters. The internal reference used for qPCR was the actin gene. (b) normalized mRNA levels of firefly luciferase driven by different promoters. The internal reference used for qPCR was the *Renilla* luciferase gene in the plasmid. The mRNA expression levels were detected by real-time quantitative reverse transcription PCR. Data are presented as the mean ± standard error of three independent replicates.

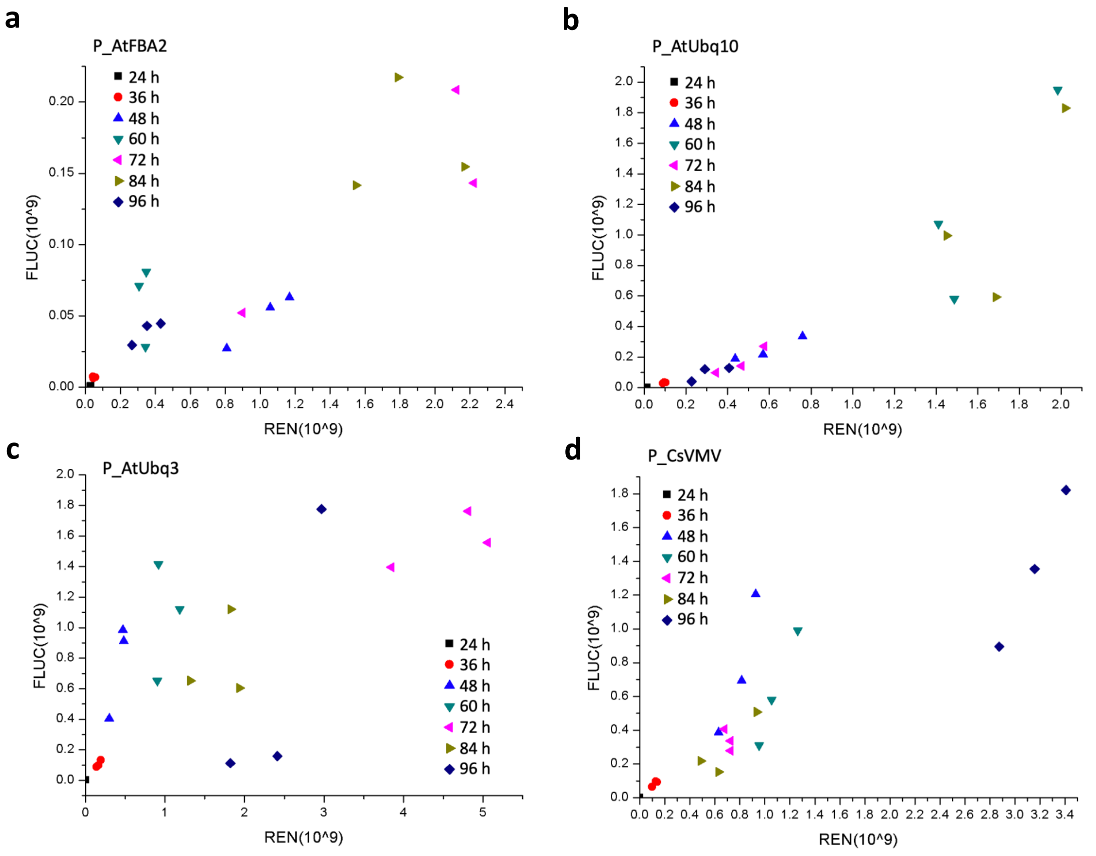


FIGURE S3. Dual-luciferase activity measurements with four representative promoters at different sampling times. Dual-luciferase activities with P_CsVMV::R-luc::T_nos and P_AtFBA2::F-luc::T_nos (a), P_AtUbq10::F-luc::T_nos (b), P_AtUbq3::F-luc::T_nos (c), and P_CsVMV::F-luc::T_nos (d).

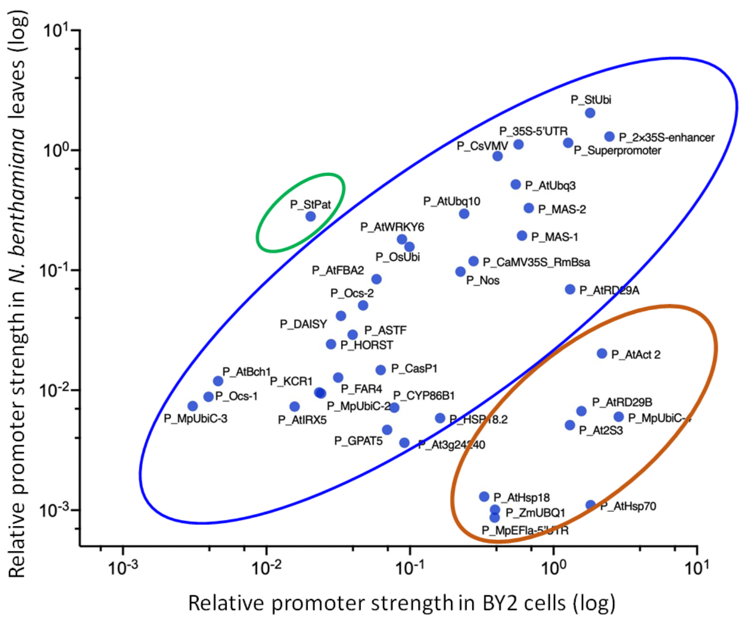


FIGURE S4. Comparison of relative transient expression levels of individual promoters in *N.benthamiana* leaves and BY2 cells. The relative promoter strengths (R-luc/F-luc activities) were monitored on a log scale. The scatter plots correspond to the relative strengths of the promoters. The plots located in the blue circle represent that the luminescence intensity is consistent within *N. benthamiana* and BY2 cells. The plots in the brown circle indicate represent that performance of promoters tested in BY2 cells are better than tested in *N.benthamiana*. The promoter P_StPat in the green circle shows a higher expression level in *N. benthamiana* than in BY2 cells.

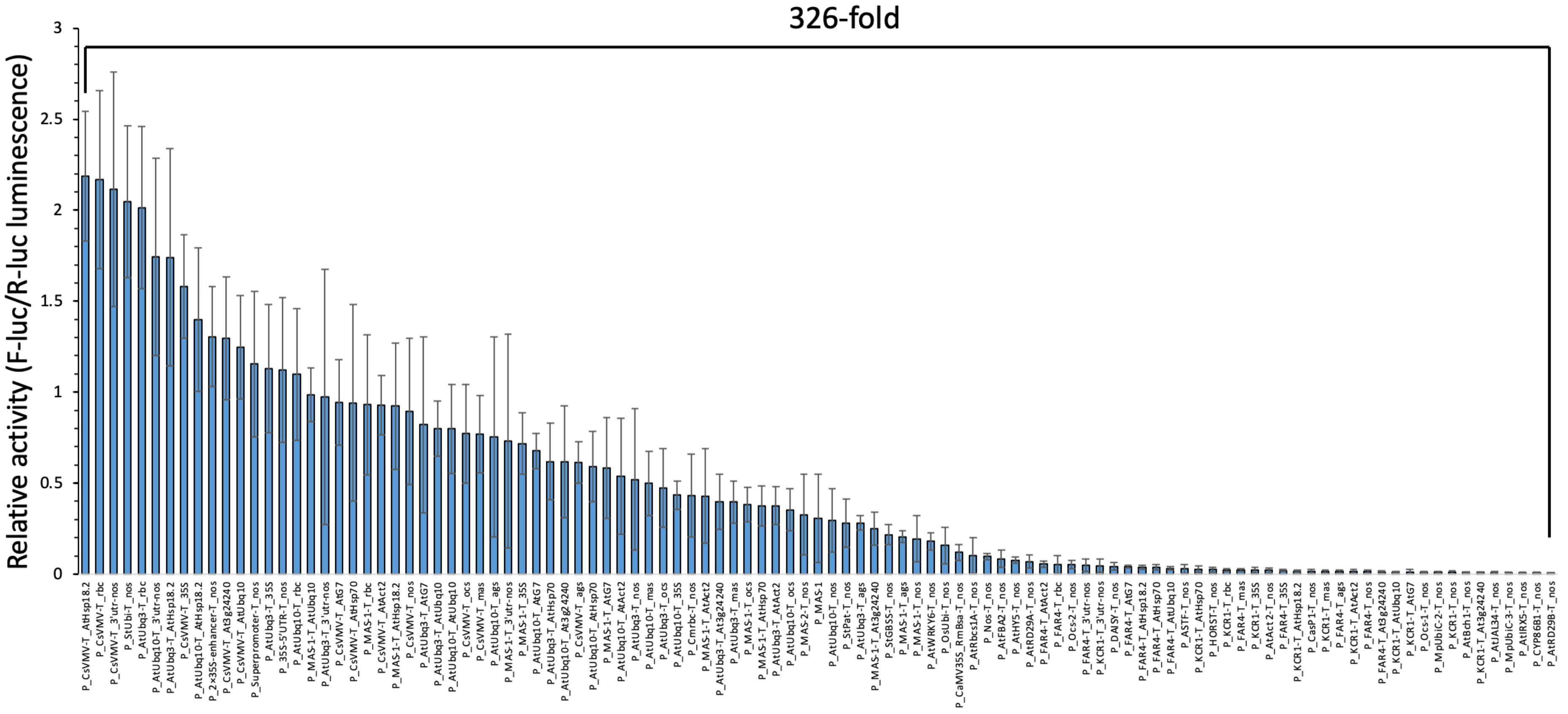


FIGURE S5. Expression cassette strength library obtained by integration of terminator elements. Expression cassette strength is presented as the ratio of firefly luciferase to *Renilla* luciferase luminescence intensity in *N. benthamiana*. These data are also shown in Figure 3 and Figure 5. Here,

we integrated different regulatory elements (promoters and terminators) to obtain more precise control of gene expression than that obtained by promoters alone.

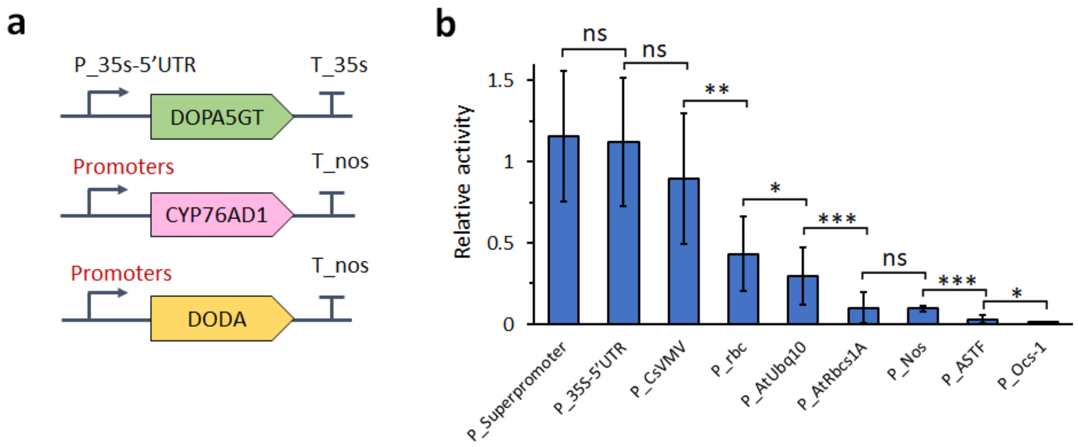


FIGURE S6. Different strengths of promoters used in betalain synthesis. (a) Schematic diagram of constructs used for betalain synthesis. DODA: DOPA 4,5-dioxygenase; DOPA5GT: cyclo-DOPA-5-O-glucosyltransferase. (b) Different strengths of promoters selected for use in betalain synthesis. The data are the same as the strengths shown in Figure 6.

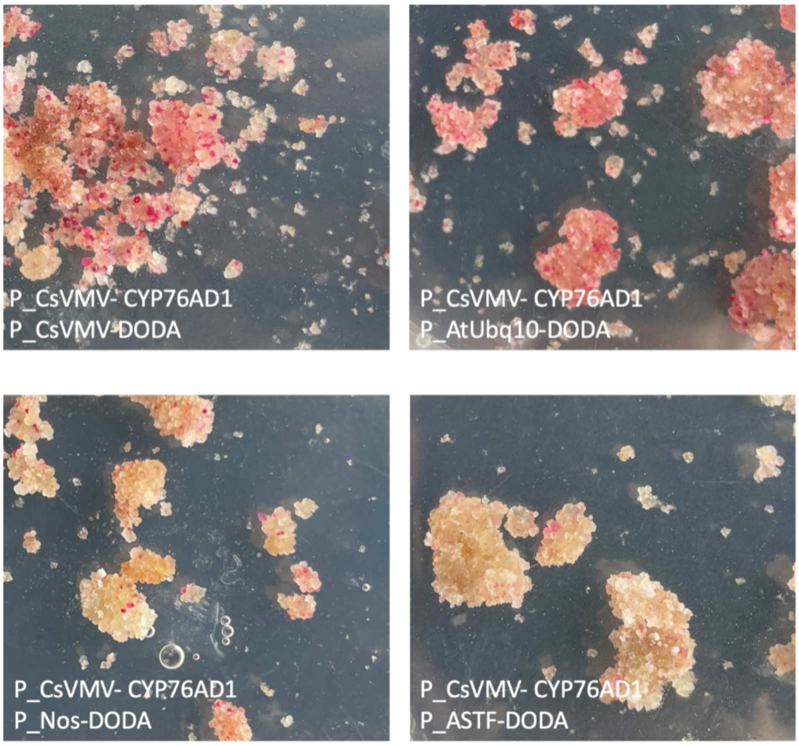


FIGURE S7. BY2-cells transformed with betalain synthesis genes. The cell-color phenotypes of BY2 cells co-infiltrated with *Agrobacterium* harboring plasmids for the expression of DOPA5GT, CYP76AD1, and DODA. DOPA5GT was controlled by the combination of P_35S-5′UTR and T_nos. CYP76AD1 and DODA were under the control of various promoters and T_nos.

## Supplementary Table 1. Detailed information on the promoters used in this study

| Element | Species | Length | Reference |
|---|---|---|---|
| P_CaMV35S_RmBsa | virus | 315 bp | Peyret, H., et al., (2013). Plant Mol Biol 83:51-58. |
| | GGAAACCTCCTCGGATTCCATTGCCCAGCTATCTGTCACTTTATTGAGAAGATAGTG<br>GAAAAGGAAGGTGGCTCCTACAAATGCCATCATTGCGATAAAGGAAAGGCCATCGT<br>TGAAGATGCCTCTGCCGACAGTGGTCCCAAAGATGGACCCCCACCCACGAGGAGCA<br>TCGTGGAAAAAGAAGACGTTCCAACCACGTCTTCAAAGCAAGTGGATTGATGTGAT<br>ATCTCCACTGACGTAAGGGATGACGCACAATCCCACTATCCTTCGCAAGACCCTTCC<br>TCTATATAAGGAAGTTCATTTCATTTGGAGAGG | | |
| P_CsVMV | virus | 520 bp | |
| | CCAGAAGGTAATTATCCAAGATGTAGCATCAAGAATCCAATGTTTACGGGAAAAAC<br>TATGGAAGTATTATGTGAGCTCAGCAAGAAGCAGATCAATATGCGGCACATATGCA<br>ACCTATGTTCAAAAATGAAGAATGTACAGATACAAGATCCTATACTGCCAGAATAC<br>GAAGAAGAATACGTAGAAATTGAAAAAGAAGAACCAGGCGAAGAAAAGAATCTTG<br>AAGACGTAAGCACTGACGACAACAATGAAAAGAAGAAGATAAGGTCGGTGATTGT<br>GAAAGAGACATAGAGGACACATGTAAGGTGGAAAATGTAAGGGCGGAAAGTAACC<br>TTATCACAAAGGAATCTTATCCCCCACTACTTATCCTTTTATATTTTTCCGTGTCATT<br>TTTGCCCTTGAGTTTTCCTATATAAGGAACCAAGTTCGGCATTTGTGAAAACAAGAA<br>AAAATTTGGTGTAAGCTATTTTCTTTGAAGTACTGAGGATACAACTTCAGAGAAATT<br>TGTAAGTTTGT | | |
| P_2×35S-enhancer | virus | 884 bp | Chung, S.M., et al., (2005). Trends Plant Sci 10:357-361. |
| | GTGGAGCACGACACACTTGTCTACTCCAAAAATATCAAAGATACAGTCTCAGAAGA<br>CCAAAGGGCAATTGAGACTTTTCAACAAAGGGTAATATCCGGAAACCTCCTCGGAT<br>TCCATTGCCCAGCTATCTGTCACTTTATTGTGAAGATAGTGGAAAAGGAAGGTGGCT<br>CCTACAAATGCCATCATTGCGATAAAGGAAAGGCCATCGTTGAAGATGCCTCTGCC<br>GACAGTGGTCCCAAAGATGGACCCCCACCCACGAGGAGCATCGTGGAAAAAGAAG<br>ACGTTCCAACCACGTCTTCAAAGCAAGTGGATTGATGTGATAACATGGTGGAGCAC<br>GACACACTTGTCTACTCCAAAAATATCAAAGATACAGTCTCAGAAGACCAAAGGGC<br>AATTGAGACTTTTCAACAAAGGGTAATATCCGGAAACCTCCTCGGATTCCATTGCCC<br>AGCTATCTGTCACTTTATTGTGAAGATAGTGGAAAAGGAAGGTGGCTCCTACAAAT<br>GCCATCATTGCGATAAAGGAAAGGCCATCGTTGAAGATGCCTCTGCCGACAGTGGT<br>CCCAAAGATGGACCCCCACCCACGAGGAGCATCGTGGAAAAAGAAGACGTTCCAA<br>CCACGTCTTCAAAGCAAGTGGATTGATGTGATATCTCCACTGACGTAAGGGATGAC<br>GCACAATCCCACTATCCTTCGCAAGACCCTTCCTCTATATAAGGAAGTTCATTTCAT<br>TTGGAGAGGACGTCGAGAGTTCTCAACACAACATATACAAAACAAACGAATCTCAA<br>GCAATCAAGCATTCTACTTCTATTGCAGCAATTTAAATCATTTCTTTTAAAGCAAAA<br>GCAATTTTCTGAAAATTTTCACCATTTACGAACGATAG | | |
| P_35S-5′UTR | virus | 830 bp | Peyret, H., et al., (2013). Plant Mol Biol 83:51-58. |
| | GGAAACCTCCTCGGATTCCATTGCCCAGCTATCTGTCACTTTATTGAGAAGATAGTG<br>GAAAAGGAAGGTGGCTCCTACAAATGCCATCATTGCGATAAAGGAAAGGCCATCGT<br>TGAAGATGCCTCTGCCGACAGTGGTCCCAAAGATGGACCCCCACCCACGAGGAGCA<br>TCGTGGAAAAAGAAGACGTTCCAACCACGTCTTCAAAGCAAGTGGATTGATGTGAT | | |

| | | | |
|---|---|---|---|
| | ATCTCCACTGACGTAAGGGATGACGCACAATCCCACTATCCTTCGCAAGACCCTTCC<br>TCTATATAAGGAAGTTCATTTCATTTGGAGAGGTATTAAAATCTTAATAGGTTTTGA<br>TAAAAGCGAACGTGGGGAAACCCGAACCAAACCTTCTTCTAAACTCTCTCTCATCTC<br>TCTTAAAGCAAACTTCTCTCTTGTCTTTCTTGCGTGAGCGATCTTCAACGTTGTCAGA<br>TCGTGCTTCGGCACCAGTACAACGTTTTCTTTCACTGAAGCGAAATCAAAGATCTCT<br>TTGTGGACACGTAGTGCGGCGCCATTAAATAACGTGTACTTGTCCTATTCTTGTCGG<br>TGTGGTCTTGGGAAAAGAAAGCTTGCTGGAGGCTGCTGTTCAGCCCCATACATTACT<br>TGTTACGATTCTGCTGACTTTCGGCGGGTGCAATATCTCTACTTCTGCTTGACGAGG<br>TATTGTTGCCTGTACTTCTTTCTTCTTCTTCTTGCTGATTGGTTCTATAAGAAATCTAG<br>TATTTTCTTTGAAACAGAGTTTTCCCGTGGTTTTCGAACTTGGAGAAAGATTGTTAA<br>GCTTCTGTATATTCTGCCCAAATTCGCG | | |
| P_MAS-1 | *Agrobacterium* | 385 bp | Langridge, W.H., et al., (1989). Proc Natl Acad Sci USA 86:3219-3223. |
| | TTTTCAAATCAGTGCGCAAGACGTGACGTAAGTATCCGAGTCAGTTTTTATTTTTCT<br>ACTAATTTGGTCGTTTATTTCGGCGTGTAGGACATGGCAACCGGGCCTGAATTTCGC<br>GGGTATTCTGTTTCTATTCCAACTTTTTCTTGATCCGCAGCCATTAACGACTTTTGAA<br>TAGATACGCTGACACGCCAAGCCTCGCTAGTCAAAAGTGTACCAAACAACGCTTTA<br>CAGCAAGAACGGAATGCGCGTGACGCTCGCGGTGACGCCATTTCGCCTTTTCAGAA<br>ATGGATAAATAGCCTTGCTTCCTATTATATCTTCCCAAATTACCAATACATTACACT<br>AGCATCTGAATTTCATAACCAATCTCGATACACCAAATCG | | |
| P_MAS-2 | *Agrobacterium* | 392 bp | Chung, S.M., et al., (2005). Trends Plant Sci 10:357-361. |
| | TGAGATTTTTCAAATCAGTGCGCAAGACGTGACGTAAGTATCTGAGCTAGTTTTTAT<br>TTTTCTACTAATTTGGTCGTTTATTTCGGCGTGTAGGACATGGCAACCGGGCCTGAA<br>TTTCGCGGGTATTCTGTTTCTATTCCAACTTTTTCTTGATCCGCAGCCATTAACGACT<br>TTTGAATAGATACGCTGACACGCCAAGCCTCGCTAGTCAAAAGTGTACCAAACAAC<br>GCTTTACAGCAAGAACGGAATGCGCGTGACGCTCGCGGTGACGCCATTTCGCCTTTT<br>CAGAAATGGATAAATAGCCTTGCTTCCTATTATATCTTCCCAAATTACCAATACATT<br>ACACTAGCATCTGAATTTCATAACCAATCTCGATACACCAAATCGA | | |
| P_Ocs-1 | *Agrobacterium* | 235 bp | |
| | ATCATTAATTTCCACCTTCACCTACGATGGGGGGCATCGCACCGGTGAGTAATATTG<br>TACGGCTAAGAGCGAATTTGGCCTGTAAGATCCTTTTTACCGACAACTCATCCACAT<br>TGATGGTAGGCAGAAAGTTAAAGGATTATCGCAAGTCAATACTTGCCCATTCATTG<br>ATCTATTTAAAGGTGTGGCCTCAAGGATAATCGCCAAACCATTATATTTGCAATCTA<br>CCAA | | |
| P_Ocs-2 | *Agrobacterium* | 365 bp | Chung, S.M., et al., (2005). Trends Plant Sci 10:357-361. |
| | CTGAAAGCGACGTTGGATGTTAACATCTACAAATTGCCTTTTCTTATCGACCATGTA<br>CGTAAGCGCTTACGTTTTTGGTGGACCCTTGAGGAAACTGGTAGCTGTTGTGGGCCT<br>GTGGTCTCAAGATGGATCATTAATTTCCACCTTCACCTACGATGGGGGGCATCGCAC<br>CGGTGAGTAATATTGTACGGCTAAGAGCGAATTTGGCCTGTAAGATCCTTTTTACCG<br>ACAACTCATCCACATTGATGGTAGGCAGAAAGTTAAAGGATTATCGCAAGTCAATA<br>CTTGCCCATTCATTGATCTATTTAAAGGTGTGGCCTCAAGGATAATCGCCAAACCAT<br>TATATTTGCAATCTACCAA | | |
| P_Nos | *Agrobacterium* | 289 bp | Chung, S.M., et al., (2005). Trends in Plant Science 10:357-361. |

| | | | |
|---|---|---|---|
| | GATCATGAGCGGAGAATTAAGGGAGTCACGTTATGACCCCCGCCGATGACGCGGGA<br>CAAGCCGTTTTACGTTTGGAACTGACAGAACCGCAACGTTGAAGGAGCCACTCAGC<br>CGCGGGTTTCTGGAGTTTAATGAGCTAAGCACATACGTCAGAAACCATTATTGCGC<br>GTTCAAAAGTCGCCTAAGGTCACTATCAGCTAGCAAATATTTCTTGTCAAAAATGCT<br>CCACTGACGTTCCATAAATTCCCCTCGGTATCCAATTAGAGTCTCATATTCACTCTC<br>AAT | | |
| P_Superpromoter | *Agrobacterium* | 1145 bp | Ni, M., et al.,(1995). Plant J 7:661-676. |
| | ACGGTATCGATAAGCTCGCGGATCCCTGAAAGCGACGTTGGATGTTAACATCTACA<br>AATTGCCTTTTCTTATCGACCATGTACGTAAGCGCTTACGTTTTTGGTGGACCCTTGA<br>GGAAACTGGTAGCTGTTGTGGGCCTGTGGTCTCAAGATGGATCATTAATTTCCACCT<br>TCACCTACGATGGGGGGCATCGCACCGGTGAGTAATATTGTACGGCTAAGAGCGAA<br>TTTGGCCTGTAGGATCCCTGAAAGCGACGTTGGATGTTAACATCTACAAATTGCCTT<br>TTCTTATCGACCATGTACGTAAGCGCTTACGTTTTTGGTGGACCCTTGAGGAAACTG<br>GTAGCTGTTGTGGGCCTGTGGTCTCAAGATGGATCATTAATTTCCACCTTCACCTAC<br>GATGGGGGGCATCGCACCGGTGAGTAATATTGTACGGCTAAGAGCGAATTTGGCCT<br>GTAGGATCCCTGAAAGCGACGTTGGATGTTAACATCTACAAATTGCCTTTTCTTATC<br>GACCATGTACGTAAGCGCTTACGTTTTTGGTGGACCCTTGAGGAAACTGGTAGCTGT<br>TGTGGGCCTGTGGTCTCAAGATGGATCATTAATTTCCACCTTCACCTACGATGGGGG<br>GCATCGCACCGGTGAGTAATATTGTACGGCTAAGAGCGAATTTGGCCTGTAGGATC<br>CGCGAGCTGGTCAATCCCATTGCTTTTGAAGCAGCTCAACATTGATCTCTTTCTCGA<br>TCGAGGGAGATTTTTCAAATCAGTGCGCAAGACGTGACGTAAGTATCCGAGTCAGT<br>TTTTATTTTTCTACTAATTTGGTCGTTTATTTCGGCGTGTAGGACATGGCAACCGGGC<br>CTGAATTTCGCGGGTATTCTGTTTCTATTCCAACTTTTTCTTGATCCGCAGCCATTAA<br>CGACTTTTGAATAGATACGCTGACACGCCAAGCCTCGCTAGTCAAAAGTGTACCAA<br>ACAACGCTTTACAGCAAGAACGGAATGCGCGTGACGCTCGCGGTGACGCCATTTCG<br>CCTTTTCAGAAATGGATAAATAGCCTTGCTTCCTATTATATCTTCCCCCAAATTACCA<br>ATACATTACACTAGCATCTGAATTTCATAACCAATCTCGATACACCAAATCGACTCT<br>AGG | | |
| P_AtAct 2 | *Arabidopsis* | 791 bp | Sarrion-Perdigones, A., et al., (2013). Plant Physiol 162:1618-1631. |
| | ATGGAGTCGACAAAATTTAGAACGAACTTAATTATGATCTCAAATACATTGATACA<br>TATCTCATCTAGATCTAGGTTATCATTATGTAAGAAAGTTTTGACGAATATGGCACG<br>ACAAAATGGCTAGACTCGATGTAATTGGTATCTCAACTCAACATTATACTTATACCA<br>AACATTAGTTAGACAAAATTTAAACAACTATTTTTTATGTATGCAAGAGTCAGCATA<br>TGTATAATTGATTCAGAATCGTTTTGACGAGTTCGGATGTAGTAGTAGCCATTATTT<br>AATGTACATACTAATCGTGAATAGTGAATATGATGAAACATTGTATCTTATTGTATA<br>AATATCCATAAACACATCATGAAAGACACTTTCTTTCACGGTCTGAATTAATTATGA<br>TACAATTCTAATAGAAAACGAATTAAATTACGTTGAATTGTATGAAATCTAATTGAA<br>CAAGCCAACCACGACGACGACTAACGTTGCCTGGATTGACTCGGTTTAAGTTAACC<br>ACTAAAAAAACGGAGCTGTCATGTAACACGCGGATCGAGCAGGTCACAGTCATGAA<br>GCCATCAAAGCAAAAGAACTAATCCAAGGGCTGAGATGATTAATTAGTTTAAAAAT<br>TAGTTAACACGAGGGAAAAGGCTGTCTGACAGCCAGGTCACGTTATCTTTACCTGT | | |

| | | | |
|---|---|---|---|
| | GGTCGAAATGATTCGTGTCTGTCGATTTTAATTATTTTTTGAAAGGCCGAAAATAA<br>AGTTGTAAGAGATAAACCCGCCTATATAAATTCATATATTTCCTCTCCGCTTTGAA | | |
| P_AtFBA2 | *Arabidopsis* | 1111 bp | Lu, W., et al., (2012). Gene 503:65-74. |
| | CCTACACATCCACTTGGCCACCTGTTGTGCAATGATGACGATGAGATTGCAGTGTCT<br>CTCGACTGATGATGTAGACTTCACAATCTCTTAAAAATCCCAGTACTTAACCTCAGC<br>CTTAAGAAAACGCAGGGAACTACGTCCTCACAAAATCTTTCTCTTTGAGTGTAACTT<br>TCAGACGCATCTTTGGCTCTAAATTCTAAAAAGGAAAATTTTAATAGGTTTTCATAA<br>TCATGGGTTCACTGGATTAGCATATAAGTCTATGGTTGAGAAACTTGAGACCCAGA<br>CTAACGAAACCTCTTTCCGGATCCAAAGGTCCTCTTGTGTAGTTGACGTGGTAAACT<br>CTCTACCGTCAAATTTAGACATTAGCTAATCTGATCAATAATCTCGGCAGCTCTTAA<br>AAATTAAAAATTAGAAATGATACGAACCTCATAATTTTTCTTTCTCTTATCAAAACA<br>CCATCTTTGTATCTTTATAAGCCTGTTGCCACTATTTTTAATTGAAAATGATGCGTTT<br>GTCTTATGTTTTCTGTCCTGGAGTTCAACATTATGACAATATGTATAGTAAATTAGT<br>GATATACAAGACGTTTGCAATTCAAGAAAAAAACTTATAAACTAATTAATATTATG<br>GTCCACGGTGCTACATATTAACTCTTGATGGTTTTATACATCTTTTCTACATGCTAAT<br>ATGCTTTTAATATTGTAGCCTAACGTTATAATTTGTTTTTCTTAAAGAAACAGTATC<br>TTGAACGAATCTTAACTATTTCTGTACTTATTCGATTTTATTCGATTTTTATCCGTGT<br>AAAGGCAAACGATTATTATGTAACGACGGGCATAAAAAGAGTATCGATTTCCTATT<br>CGGAGAAAAAAAAAAAGATAAAAATTGGAGTGTATGTATATTTCTTGAATTGAGAG<br>TAATACAAGATTACGGTCCAGGTGGCGGAATATTATTGGCAAGGTCACAAGAACCT<br>CAAATAACTGATCTGAAGAGAAATATAAATCCAAAAGAGGGAGGAGTGAGAGATA<br>AGGGTGGTGTCATAAGCGTTTACTGTGAGTCTCTCAAAGAAACCAAAGGCAGAGAA<br>AAGAGATAACACACACAAAAAAAA | | |
| P_AtRbcs1a | *Arabidopsis* | 1183 bp | Farhi, M., et al. (2011). Nat Biotechnol 29:1072-1074 |
| | AACATTCATACGTTTTCATTTGAAATATCCTCTATAATTATATTGAATTTGGCACATA<br>ATAAGAAACCTAATTGGTGATTTATTTTACTAGTAAATTTCTGGTGATGGGCTTTCT<br>ACTAGAAAGCTCTCGGAAAATCTTGGACCAAATCCATATTCCATGACTTCGATTGTT<br>AACCCTATTAGTTTTCACAAACATACTATCAATATCATTGCAACGGAAAAGGTACA<br>AGTAAAACATTCAATCCGATAGGGAAGTGATGTAGGAGGTTGGGAAGACAGGCCC<br>AGAAAGAGATTTATCTGACTTGTTTTGTGTATAGTTTTCAATGTTCATAAAGGAAGA<br>TGGAGACTTGAGAAGTTTTTTTTGGACTTTGTTTAGCTTTGTTGGGCGTTTTTTTTTTT<br>TGATCAATAACTTTGTTGGGCTTATGATTTGTAATATTTTCGTGGACTCTTTAGTTTA<br>TTTAGACGTGCTAACTTTGTTGGGCTTATGACTTGTTGTAACATATTGTAACAGATG<br>ACTTGATGTGCGACTAATCTTTACACATTAAACATAGTTCTGTTTTTTGAAAGTTCTT<br>ATTTTCATTTTTATTTGAATGTTATATATTTTTCTATATTTATAATTCTAGTAAAAGG<br>CAAATTTTGCTTTTAAATGAAAAAAATATATATTCCACAGTTTCACCTAATCTTATG<br>CATTTAGCAGTACAAATTCAAAAATTTCCCATTTTTATTCATGAATCATACCATTAT<br>ATATTAACTAAATCCAAGGTAAAAAAAAGGTATGAAAGCTCTATAGTAAGTAAAAT<br>ATAAATTCCCCATAAGGAAAGGGCCAAGTCCACCAGGCAAGTAAAATGAGCAAGC<br>ACCACTCCACCATCACACAATTTCACTCATAGATAACGATAAGATTCATGGAATTAT<br>CTTCCACGTGGCATTATTCCAGCGGTTCAAGCCGATAAGGGTCTCAACACCTCTCCT<br>TAGGCCTTTGTGGCCGTTACCAAGTAAAATTAACCTCACACATATCCACACTCAAAA<br>TCCAACGGTGTAGATCCTAGTCCACTTGAATCTCATGTATCCTAGACCCTCCGATCA | | |

| | | | |
|---|---|---|---|
| | | | CTCCAAAGCTTGTTCTCATTGTTGTTATCATTATATATAGATGACCAAAGCACTAGA<br>CCAAACCTCAGTCACACAAAGAGTAAAGAAGAACA |
| P_AtUbq10 | *Arabidopsis* | 643 bp | Sarrion-Perdigones, A., et al., (2013). Plant Physiol 162:1618-1631. |
| | | | TACCCGACGAGTCAGTAATAAACGGCGTCAAAGTGGTTGCAGCCGGCACACACGAG<br>TCGTGTTTATCAACTCAAAGCACAAATACTTTTCCTCAACCTAAAAATAAGGCAATT<br>AGCCAAAAACAACTTTGCGTGTAAACAACGCTCAATACACGTGTCATTTTATTATTA<br>GCTATTGCTTCACCGCCTTAGCTTTCTCGTGACCTAGTCGTCCTCGTCTTTTCTTCTTC<br>TTCTTCTATAAAACAATACCCAAAGAGCTCTTCTTCTTCACAATTCAGATTTCAATTT<br>CTCAAAATCTTAAAAACTTTCTCTCAATTCTCTCTACCGTGATCAAGGTAAATTTCTG<br>TGTTCCTTATTCTCTCAAAATCTTCGATTTTGTTTTCGTTCGATCCCAATTTCGTATAT<br>GTTCTTTGGTTTAGATTCTGTTAATCTTAGATCGAAGACGATTTTCTGGGTTTGATCG<br>TTAGATATCATCTTAATTCTCGATTAGGGTTTCATAGATATCATCCGATTTGTTCAAA<br>TAATTTGAGTTTTGTCGAATAATTACTCTTCGATTTGTGATTTCTATCTAGATCTGGT<br>GTTAGTTTCTAGTTTGTGCGATCGAATTTGTCGATTAATCTGAGTTTTTCTGATTAAC<br>ACT |
| P_AtHsp18 | *Arabidopsis* | 838 bp | Farhi, M., et al. (2011). Nat Biotechnol 29:1072-1074 |
| | | | AAGCTTTTCTCTTCATTTCTCTTTCTTTCTTTCTTTCATGCAAGTTCTATTGATTAGAA<br>ACCAAAACCATGACATATCACATTTTCAATTTTTAACCCCAAGTTTCCCAAAAAGGT<br>AATCACAATTACTTTCCATGGTCATTTCTTCTGGTTCAAGCATGACATGAACAGGCA<br>ATAAATAAGTTGAGATTTTGATCACAGTAACTGATACTTGAATCGAATCATTTAGAT<br>TTTTTTTTTTTTTAGTTTACTTGTTTAGTAAATATGTTGTCTATGTTTGTCACAAAAAC<br>GTGGCTCAGTTCTTGTATATATGGAGACAAAAAAATCCATTAAAAGATTGTTGACAT<br>TCTCGGAAATTTAGTGCCAACTGTTATTGCGAGAACTTACTATAGTTTTCCTTTGGC<br>GAAAAGCTAATAATCTTAAATCTTGATTTTGTCCTCTTTTCTCTGAGTTAGATTTTCT<br>TAAATTCCACTTCCGACCTATTAAGAAATGGGCTTTTGCAAAGAAGATCCGCTTCAC<br>TGAGCCCGTATCTCGAAGAGGATAATACAACAACAAAGCAAAACGGCACGTAGTTT<br>TAATTGTAACCAAGGATTGCATTTCGGTCTTGTTTCAACAAACGAAACTTCCTGAAA<br>TGCCAAGAAAAATCTGGTCATTTCACCACAGTGATCATTGTGTATGTGTTCTAAAGA<br>CTCCAAGCGAAGGTTTTAGAAAAAGGAGCATTTTCTATTCTATTCAAGAAACTCGA<br>AGAACATTCTCTCTTCATCCTCTAACTTCCCTATAAATATGTCCTTTGCTAATCAGAT<br>CAAATCAGCAGGAAAATCAAGAACCAAAAGTC |
| P_AtHsp70 | *Arabidopsis* | 1982 bp | Sung, D.Y., et al., (2001). Plant Physiol 126:789-800. |
| | | | GAACTGCGAAAAAAGGGAGCAATAAAGTTCAGAAACAAGCCCTTGACTGTATGTTG<br>AGAAGAAATAAACATTACCTGCCATGGTCTTTCCCAGTCAAGTGGCATAGGCCAAG<br>CTCCAAACCATCCTCCAATAATTGCTCCATATGCTGGAACTAAGAGCATATGTTCTA<br>TAATTCCGATTGGTCTAAAAGAAAAAAAAAAGATGGATTAACAACAACATAAAGTG<br>TGTGAATTATCATACTAGGTAGCATAAGTGACTTACTTTAGTGAAGCAAAGATACG<br>ATGCCAATCTATCCATGATGCACCTAAAACTGCAGTTGCTGGTACAACCTATGAAA<br>ATCAAAATCATCAGACACCATCAAAAGGTTTGTTTCAAACTGCAGAAATACACATA<br>TGGCATGGTATTAACATCTTTTAAGTCATGGACACTGCAGCTTACCGTGAAAACAGA<br>CATTAGAAAGGACCAGTGAATTGTTTTTGATAGAGATCTGCATAAGATGAGAAGGT<br>AATACTCATGTCAGCTTTTGAAAATCACACAATCTCCATGCAGAAGTGAGACCACA<br>ACCAGCATTAAGGAGCCTCATTTTTAATCTGGACAACTGAAAAGTCCAGATTTACA |

| | | | |
|---|---|---|---|
| | ATCAACATTTCCACAATTAGAAAGAAATAAAAAGATATAGAGAAAGAAGATCTTAG<br>TGAAACATACTGCATTCCAATAGGTGCACCCAAAGAAACAGCTCCCAAAGCATTTA<br>TAAGAGCACCTATTAAACAAATCAAAACACAGGTATAGATATAGTAGACACAATCA<br>AGGGATACTGAATCAGCAAAAGCACAAGATGAAAGCATAAGAAGCTCAGACTCAC<br>TCACATCTTCAGGTTTAGCTTAACATCTAGAATTTCCAGGTGAACTCATTGAGCAAT<br>CAACTTCTTTGAAAAATTAGTGAGCTATAATAAGCTGAAAAACAGAAATACTAACC<br>GGCAATTAGTCCGACTAAGCTTCGGCCAACAGCTCTAAAGTACTGGAAATCAATGA<br>GAAAATCATGAATACAGAGTCAAAACACACAAGACCAAAATAATACTATATGAAA<br>AGAAACGATTTTTTTGAGCATTAAGAGTGTAAAGTGAGGATACTAGCTTGTTTTGTC<br>AATTTCATAAAAGGGAAATGAGACAAGCACAAAGCAATCGAGTTAAAACGAGAAA<br>TTCAGTTTCTTTAATTCTCACAGAGAACCTCAGAGATGAACTATACTCACCGAGCAT<br>TTCTCTGGGTTTCGTCGGAACAAGCTGTAGATGATTACCACGATCGGGAACTCAATA<br>ATCTGAATATCAACATCAAAACAAAAAGGCTAAAATTAACTGAAAAATATCCACTA<br>GCAACCAGGTTATGAAAGAAAGTTTTAGTACCCATAGGAGACGCAGAGTGAGAGTT<br>GGATCAGAAATGAGATCGACAGAGTATTTGTTACGGACCACGTGAAATCCGAAGAT<br>CAGAAATAACCCAGTAATCACATAAACAGCAAAAGCCCCCCAAGTTGATATCGTGA<br>TACTAACGGAGATTTCTGGATTCTTCTTCTCCTTCGCCTCTTTCATGGCTTTTCCTTTC<br>TCGTCTTCGAAATCACAGAACAAGTGAAGAAAGAAGACGTAAACAAAATATTGAA<br>AATCCTCCAGAACTTACACTGGGCCTTTTATTCTATATACGGGCCTACAAGTTTATA<br>CCATATGGGCTTTAATAGGCCCATTTAATTATCAAGCGGTCGCCGGAGATAAAATAT<br>ATCCCGGTCGGTGAATCCAGAACTCTCTTGTACGTTTGCGCGATTTCTCCACCTTTCC<br>ACAATCCCCTGGGTTGTGCCACGACCTTTTTTCTCGAAATGTCTCGTTCCTCTCGTCG<br>GATTCGTATATATAGCTTCTTCCATCGTTTCCGATTCTTCATCAAACAGATAAACAA<br>ACAAAAGAAATCGAAAAACCTCACTTCCAATTTCATTCAATTACTGAAGCTTTTTTT<br>TAGCA | | |
| P_At2S3 | *Arabidopsis* | 350 bp | Emami, S., et al., (2013). Front Plant Sci 4:339. |
| | GAAACCAAATTAACATAGGGTTTTTATTTAAATAAAAGTTTAACCTTCTTTTTAAAA<br>AATTGTTCATAGTGTCATGTCAGAACAAGAGCTACAAATCACACATAGCATGCATA<br>AGCGGAGCTATGATGAGTGGTATTGTTTTGTTCGTCACTTGTCACTCTTTTCCAACAC<br>ATAATCCCGACAACAACGTAAGAGCATCTCTCTCTCTCCACACACACTCATGCATGC<br>ATGCATTCTTACACGTGATTGCCATGCAAATCTCCTTTCTCACCTATAAATACAAAC<br>CAACCCTTCACTACACTCTTCACTCAAACCAAAACAAGAAAACATACACAAATAGC<br>AAAAC | | |
| P_AtBch1 | *Arabidopsis* | 1985 bp | Shih, P.M., et al., (2016). Nat Commun 7:13215. |
| | TGCCTTAATCTTGTCTGCAATGTAGGGTATTGATGTCTTGAACGGCATAGTGACAAG<br>CAACGCCTATTTGGTAATAAATCTCGCTATGCATGCGTTTTCGAGTGGTGTTGACTA<br>TTTTGCTGACAAGGAGGATGTTAGCTATTGTGATTTAGTGCCACCAGCTGAACCCCT<br>TTTAAGTATAATGAAGGAAATACCACCGCTGAGATAAGGGTGTTCAATGGAGAAGA<br>TGCAAACTGCACGACCCTAGGAACGTGAGGCAAAGAGACGAAAGTTTGGCGCTCTT<br>GTTCCAGAGGACCAGTTTCTAGCTCAAAATCCGGTAATGTTTTTGTTTTCCTTCCTTG<br>TGACTAGACTCGTGTATCTAACTAGCAGTAATACATTTTCAGGGTCCCTTTAGGATC<br>ATGATCAGAGTTTCTGGTGCAGACGACTATGTGGAGTCTTTGAAATCACGGTGGACT<br>CGTTATCACACAGACAGATGTTAAGTGGAAAGCTGGGGTTTTAGAGGACAACAAGT<br>CATTGCACATTACAATGTTGGAGCAGGAGAAATTCTAACAGTGTCTTGGTGACAAG | | |

| | | | |
|---|---|---|---|
| | | | GTTTTGTGCACATTAGCAATAGTCCGGTCATTCTTTAATGCAAACCATAATCTATA<br>TTCATCCCAAAATCTCAGAATCCAAAGCGCAAGAGAATCAAACGTTTCATCATCTTA<br>TATCTACAACGGCCTTTAGCAGAAGAGTGTAATTGAAGGTCCATGACCCAAGTTGC<br>AGTATCCATATCACAATTTGAGGTCCAAAAAAAGAAAAAAAAAATAATAATTCTAT<br>AAAAGAACCAAAAAACAAAGATACTAGGAGGATTTGTCATAGTGAATAGTGATGT<br>GACACGTAGAGAAAGCCCATATGGCATCATCAGCCTCATAAATGGGTGTAACTTGT<br>AAGGAACTTGTTGCGTTCGTTTCTGGTTTGGAGCCTAACTTTTTGTTTCTTTGTCTTT<br>CACCATCGCTGTTTCTGTGATTTTACGTTTATGCCATTTTAAGTTAAGGACTTAGTCC<br>AGACATCTTAGAATTACATCATTATTCATCACGATATCGAAAATTATACAAATGACG<br>TTTAGAACAAACGGATATAAACAATACAAAATCACACGCAATACACGGAACTATGA<br>CAACCATGCCACAAGTCTCCACATGTACTCATGTAGTGATGATGTAGCGTCCTAAAC<br>ATATCCACATTCCTTAAAATGAAAAAATCTACTTTTTAGAAAACGAATCCAAAACA<br>ATAGACAGTAAATGAATCTAGTAGAATTTCTACATAAGTTTACTTTCTCTATATATC<br>TTATATTTTATAACAACAATAATGTTTATAAATTTGTTTCACCTTAATTATGGTTTAC<br>AAAATACTGTATCTTGGATTTAATTAAAAAAGATAAATCATTTATAAACTAAATTGA<br>TGTGATTCATACTAAAATACTAAAGTGTTCTTAAGATATTGTTAGCTTTGTAGACAA<br>GTGAGAAGAATAAAAAAAGGCTTTTGTATGGTTTAAGTCTTATAGTAAATGCTAATT<br>TGGTTTATAAAACATATTTATACTAAAAATCTAGAAATTAATAAATTAAACATAACT<br>GAGCTTATGTGAAAAAGGTAAGCAAATACATTTTTGGTCAAAGAAAAGATGGAAAA<br>AAATCCAAAAAAGAAAACAAATATTATCAAAAAATAAGAAAGTAACCCATTTTGA<br>GTGGGGGTAATAATGTAAATGTGATATAAGGGTGGGGTAGGATTAGTCGCTAAAAA<br>AACTTGAGGGCAATTTTAGTCACACGAAACTAAACCATCCTTCACTTGTTTAATTTA<br>ATTTTGAAAAGGCAAAAAAAAGAGCACTTACTTTTACCCAAAAACAAAAAAATCGT<br>GGCCTCACTTGCATTTAAATCCATCCACACCACATCTCTCTCTCTGTCTATCTATCTC<br>TCTCGCTCGCATAAAAGGTTAACCTCAAAACAAGTAGCTCCTCCTTCCATTAGG |
| P_AtUbq3 | *Arabidopsis* | 1970 bp | Sarrion-Perdigones, A., et al., (2013). Plant Physiol 162:1618-1631. |
| | | | CAGAGCTAAATTCCAAAAAAAATATGATCTTTGAAACTTGCATCAATAACTAACGA<br>TTCTAGGAAGAGCCCATTTCGATTTCAACAAAAAGATGAGAGCTTGATTACATATA<br>AAATCAAGAGAAATTAACGACATTGAAGAGAATTCGATCTCGAAACCGGAAACAA<br>AGACTTATAAGTCGATTAGTCTAATTCAATGCAGAAGAAAGAATACCAGGTTTGGC<br>CTTCACGATGGAAAGCTTCGGATTTGGAGCCAAGTCTCATAAACGCCATTGTGGAA<br>GAAAGTCTTGAGTTGGTGGTAATGTAACAGAGTAGTAAGAACAGAGAAGAGAGAG<br>AGTGTGAGATACATGAATTGTCGGGCAACAAAAATCCTGAACATCTTATTTTAGCA<br>AAGAGAAAGAGTTCCGAGTCTGTAGCAGAAGAGTGAGGAGAAATTTAAGCTCTTGG<br>ACTTGTGAATTGTTCCGCCTCTTGAATACTTCTTCAATCCTCATATATTCTTCTTCTAT<br>GTTACCTGAAAACCGGCATTTAATCTCGCGGGTTTATTCCGGTTCAACATTTTTTTTG<br>TTTTGAGTTATTATCTGGGCTTAATAACGCAGGCCTGAAATAAATTCAAGGCCCAAC<br>TGTTTTTTTTTTTAAGAAGTTGCTGTTAAAAAAAAAAAAAAGGGAATTAACAACAAC<br>AACAAAAAAAGATAAAGAAAATAATAACAATTACTTTAATTGTAGACTAAAAAAA<br>CATAGATTTTATCATGAAAAAAAGAGAAAAGAAATAAAAACTTGGATCAAAAAAA<br>AAAACATACAGATCTTCTAATTATTAACTTTTCTTAAAAATTAGGTCCTTTTTCCCAA<br>CAATTAGGTTTAGAGTTTTGGAATTAAACCAAAAAGATTGTTCTAAAAAATACTCA<br>AATTTGGTAGATAAGTTTCCTTATTTTAATTAGTCAATGGTAGATACTTTTTTTTCTT |

TTCTTTATTAGAGTAGATTAGAATCTTTTATGCCAAGTATTGATAAATTAAATCAAG
AAGATAAACTATCATAATCAACATGAAATTAAAAGAAAAATCTCATATATAGTATT
AGTATTCTCTATATATATTATGATTGCTTATTCTTAATGGGTTGGGTTAACCAAGAC
ATAGTCTTAATGGAAAGAATCTTTTTTGAACTTTTTCCTTATTGATTAAATTCTTCTA
TAGAAAAGAAAGAAATTATTTGAGGAAAAGTATATACAAAAAGAAAAATAGAAAA
ATGTCAGTGAAGCAGATGTAATGGATGACCTAATCCAACCACCACCATAGGATGTT
TCTACTTGAGTCGGTCTTTTAAAAACGCACGGTGGAAAATATGACACGTATCATATG
ATTCCTTCCTTTAGTTTCGTGATAATAATCCTCAACTGATATCTTCCTTTTTTTGTTTT
GGCTAAAGATATTTTATTCTCATTAATAGAAAAGACGGTTTTGGGCTTTTGGTTTGC
GATATAAAGAAGACCTTCGTGTGGAAGATAATAATTCATCCTTTCGTCTTTTTCTGA
CTCTTCAATCTCTCCCAAAGCCTAAAGCGATCTCTGCAAATCTCTCGCGACTCTCTCT
TTCAAGGTATATTTTCTGATTCTTTTTGTTTTTGATTCGTATCTGATCTCCAATTTTTG
TTATGTGGATTATTGAATCTTTTGTATAAATTGCTTTTGACAATATTGTTCGTTTCGT
CAATCCAGCTTCTAAATTTTGTCCTGATTACTAAGATATCGATTCGTAGTGTTTACAT
CTGTGTAATTTCTTGCTTGATTGTGAAATTAGGATTTTCAAGGACGATCTATTCAATT
TTTGTGTTTTCTTTGTTCGATTCTCTCTGTTTTAGGTTTCTTATGTTTAGATCCGTTTC
TCTTTGGTGTTGTTTTGATTTCTCTTACGGCTTTTGATTTGGTATATGTTCGCTGATTG
GTTTCTACTTGTTCTATTGTTTTATTTCAG

| | | | |
|---|---|---|---|
| P_At3g24240 | *Arabidopsis* | 1628 bp | Shinohara, H., et al., (2016). Proc Natl Acad Sci USA 113:3897-3902. |

GAAAAAGTGATGGAAACTCGGAAAGGAAACATAATTAACACGAGATTATGTTAAT
GATTTTATTTCTCAGACTGTATTTGTATTAGATAAAAAATCAATAAACCATTAGAAA
TGGAAAAAAAAAAAATGAAAATTCATTGGATCTAGCAAAAGGTTCAAGTCTAGTCA
TCTTATTTCAAAGGATTCTTAATGTGTGAAATAAACAGCAATACAACTATATCTAAT
TTATAGAGTAACCTTGTTTTCTTAAGATTACATTTCGGCTCAAAGGTTTAAAGTCAA
GAAGATAACAACTATAGAAAGAAACAGTAAGCTACTAGAGTATTAACTAACATATA
CAATAAAATATACTCAAGTAGAAACAAAGGGAATAAAAACGAATCACTGATTCAA
CCACTTGCTTGGGGTTTGAGCAAACGCACTCGTTTACTTACAGGGGATTAAGCTAAA
TAATTTCTTTAAAACAATCTTAATTAACTGATCCAACAGTAACATAAATAAAAATAC
TTATACCCATAAATTTACGTACCAACGCATATATATCAGACGGCTATTGAGTCCAGT
TAGCACAAAACTGTTGGAATCTCGATAATAGCATGAGCAACTAGTTATTTTTGTATA
ATAAACTACGATGTTATATAGTATATAAGTAGATAAAACATTCTAATCTCGAAGTA
ATATTTATATACACTTTTCGATTAAGCATAATTTGGCTTGTTTTAGTTTTACTTCAGC
TTAGTCCTAAGAGATGAAACTGAACTTTTGTAGTTTTTACTTATATCTTTTACAAATT
TAACCACATTATATTAGATCGACAAAAGGGGAATTAAAATAAGATATAAGTATGTA
CATTACATGATTCACGTAAACTATAAATTACACTGCACATACCACTAGTGGTTATAT
CACCAACAATAAGCATGTATCCATAGGTAATTTTGTACTAATTCTAGCTATTTAACA
ATAGAATTTTCTTTTTAATAATTTTTATCCGGAATTCTATACCGAGATAATCATATTT
TTTCTTGAAAACTATATTGATTAGATATTTACATAGTAAAAAAAATAGAATATTTCC
CCATATTGAAGAGCATTTTAATTAACCAAAATTTTTTTGTTACCTTTACCATGTCTTT
AACCAAATATGTTTAGCTATTTACTTAATATCACTTTTAAACATCTTACCTTACGACT
ATTTCGTTTAGTTAGATATGTTTCTACTCTCCACGCAATGTATATGTATAATAATACT
AAAGAAAATTAGTACTAAAATATTACTGGTAAAGAAGAGATAGGCCATAAATAGGT
GAGGTGGCAAAGAGGTGGGGTGGTGCAGTTTTGTTGATCTCAAGGCATTGTGTTTCC

CTATTTTCCTTTGTACTCTTTCTCCTCTCTTAAATCGCCACTCCATTATTTTAAATTTC
TTATCTTGATAAAACACACACACACACAAGTATTAGAAGAACTTAAACCCTCTTATG
TTCTCTTCCTAAGCTTGAGTTTAATTCAAGCAAGAAACAGATTTCGAGAGAAGAGTA
AGCACAGAGATAGAGAGAAAGAAGGCACCAAACCCTAAATGGGTCTAAACCCAAG
ATTTTAATCTTCTTTTCGTATTTTGAGGCAA

---

P_AtJAL34 *Arabidopsis* 1471 bp Yamada, K., et al., (2011). Plant Cell Physiol 52:2039-2049.

GCTAATGACATTAGATCGTAAATAAGTTCAGGATTTATTTGCTAAAATAGCTACAAA
AATGTATATATAGATTTCAAAATATACTTTTTTAATCACTATTTTTTCGTGGAGTAGC
TGTCTACGAATGTTCTATCCAATACATTCGAACACGTGATTGTTCGTTAATTTTCTTG
ATTCTGTAAGAGAAACAAAAAATATAGATGTCCAACTTTTTTTTTCGGGTGGGAATA
TAGACGTCCAGCTTAGCTACGTACTGAATAATTCAAAGTTCCAAACTAGTATATATT
AATACAATTGACGATAAGGTCATAAGGATCGATGGATTCCAACGATTCGATACAAG
TATTAATGAAATAAGATAACACGATTGTGACAGCAAACTCTATATTGATATTTCTAT
TTTTTAATTAGCCATGCGTTGCACGATCAATTTACAAAATAATAAAAGAAAATGATC
GATCAAAGAGCATTCCATTGAAATTTAATTCCATCCTGTAATCACATAATTTTGGGC
CCAATCCTATTTTTCAAATGTAACATGCTATTACATAGTCACATAGAACATCCTAAA
ATAGGGTTAAAATGTACTTTTATCTATTTGCAATTTTGATATTTTCCTTTCTGAAAAA
GATTAGTATATGGCAAATTATCTTTTAGATAAAAGATCTTTTGTTCTGACTATACATT
AATTTATTTTAAAAAAAAAACTTAACAGATATATTTGCAAATACAAAATGATGAAA
AATAAAAGGGATACCATAATCTAAAATCTGACAAAGAAAATATACAAAAAGTCAA
TTACGATACTTAGAAAAAGAACTATATATTTTTGGGTAGGGAAGTTCAAAAACAAA
TTACCGATTTGCTGACTATATGAGCAATTATTACATACTTTTATTTATTTGTACAACA
ATTATTACACATACTTGTGTGGACCAACATGATTAATTTTATATTGGCCATATGGTG
CGTAGTAAATGTTATAATAACTTGAAATTAAATAATAACTAAGCTCGACTCGATATA
TAGATCCAACCAGTAGCCTCTCTTATTCACACCTAATCTTCATCTTCATCTTCGCATT
CATAGTCTCTACGATCAGGTAATCCCCCTCTCTCTATCTATCTTTCATATATGTGTGT
ATGTGTAAACTATCTATATTCTGAAAATAGATCAATCAATTGATCTTTTCCTATCTCA
ATTGTTTTCACAACCATCAGTTTGACTTTTGATCGTTTAAGGCTCGAGAGAATTATC
ATTCACTGTAGTAAAGATAGTTTATACCAACAAACCCATTTGGTGTTGACCAGCTTT
CAACATAAGTATGAGTTAGAGCTAGAACCGGATTAGTATTAATGTTACTTGTACCTG
TTCATAGTACTAACCAAAAATGATCCAAAAAAATGAAAATAACAAATAAACCATTT
ATGGTTATCACAGATAGATAAAAGAAGTCAACAACG

---

P_AtWRKY6 *Arabidopsis* 1718 bp Robatzek, S., et al., (2002). Genes Dev 16:1139-1149.

GTCGGTGACAAGCAACGAAACATAAATAATCGACTTTGTCAAGATATGTATAAAAA
TCGACTTTGTATTTAGAGAACAAGGTCTTCATTATTTTTCTTTTTTAACATTAGAGAA
AGAGAACTAAAGGTCTACTTCAAGAAAACAAGAAACTAGGTGCTTAGCTTTAATAA
TATATCAAAGAAAAATGGGTCAAAAAATAAATTATTACATGAGTGGGGCCCGGCCG
GCCACGGAAGTAAGACCTAACGACCAGCGCAACCGGTCACCAGTCAAGACTATTAG
AAGCCGGGCCGTCAAAATCGTTGACTCGGTTTTTGTTTCCAACTGTTTTTGTATTTTG
TTAAAAATAAATTAATACTAATAAATAAGCTGAAAAAAAAGAAAAGAGAAAAGGT
ATAATCACGTGGAACGCGCCGCAGTAAACGGAGCGGTGGATCAAACTTTTCGTCCG
TTTGATCAAACAGAAGAGAACTAGTCAATGCTCTTTCTTCATATCACAATTTAATAG
TCTCAAGACGATTACGCCACATAACCATTTTCTCGTGATTTCGACATCAAAATTTAA
TAAAAGGAACTGATTGATTGGTCATCATGTTACAAGTGTCAAATGAGCTAATCCGTT

| | | | |
|---|---|---|---|
| | TTACAGTGGCATAGTTTACGATCAATTTACAAATTTTTGGTTTTATAACATACTTGTA<br>GTTAAAACTATTTATAAGCTATTTATAGTGAGTTAGCTTATAAAACCCTATTCTTTTA<br>TCTAAAATTATGTTTTGACTCGTTTCATGATAAAATTTTATCCTTTTCATCGGAATAA<br>AAAACTTTATTATTTATTTGGCAAAATAATTGGTGTAAAAATTATGTATATGTTAAT<br>AACAAAAAATATTAATCTGATTCATAATCTTAAAAAAGAAAAATTTCTTGAAATAA<br>ACTTTAGACATTGTAAATAAAAAAACATTATTTTTATATAATGGGATGTTTATATGT<br>AATTTTTTATAAAAAAATAAAAGTTGTTTACTAGTAATCCGATTGGCTTTAACTATC<br>GTCGCCAAAAGAATAATGTAGAACTGACTTTGAGGTAAAACTAAAAGAAATTTGTA<br>AGATAATAGTCACATTAAATGCTAAAATTAATACATACTGATATATCGTATAAAATT<br>TATGAAAACTACACCTTAACCTGAATCATACACTGTAATAAAAAAAACAAATTATA<br>TATAAACCCTAAAAACTAATCATAAATCCCAAACGGTGTACTCTCTATTAGCTTTGA<br>AAGGATTGCCCAATTGTTTGTTAAAAATTTCTAATAATAGTACAATGTTTTGTTTCAT<br>TTTTCCTTTTCGTCAACCTGTTACCCAATAGCAAATGAAGTTTTTATGTGTGTGTGTG<br>TGTGTGAATTTCCATGAAAATGAAACGGGCTTAGAATCCCGGTGTATTATGGGTCG<br>GGTCGTAACCGGGCAATGACGCAGGATCTGACGTAAAACTCCCAAGAATTTTTTA<br>AAAAGTCTCCGGAAAATAAAATCAAAGTTCATTAACTTAAAAAGAAAAAACAAAA<br>TCGGTCCACGTCCCAAACCCTTTTTATAGGAGAGTCTTATGTTCTGGCAGAAGACTT<br>CACAGACTCTTTCTTAATCTCTCTCTCTTTCAACCAAACCCCTAAACAAAAAAAAAA<br>TACATTTTCTGATCTCTCTAAAAATCTTTCTCCTTCGTTAATCTCGTGATCTCTTTCTT<br>TTTCTATAT | | |
| P_AtHY5 | *Arabidopsis* | 1700 bp | Chattopadhyay, S., et al., (1998). Plant Cell 10:673-683. |
| | GAGCATTGGGTGTTGGTGACGATGTTGATGACATCGACTTGTTTGGTTCAGTCACTG<br>GTGATGTTGATGTTGATGTTGATAATGATGATGATGATAATGATGATGATGATAATG<br>ATGATGATGATGATGACAGCGAAGAAGATGAAAGACCGACTAAGCTGAAGAATTG<br>GCAGCTTAAAAGACTGGCTTATGCATTGAAAGCTGGGCGTCGTAAAACTAGTGTAA<br>GTTTTAGTTCAGTGTTGAAGTGATGTTGAATACATTGTGTAAAGCATAGTGCTTGAG<br>TTAGTGTCTGCTTTGGAGATTTTGCTTACGAATTGTTTAAGAGTTGACAAAGAACAA<br>GTAGTTCTCTGGTTTTCAAATGCATAATTGATAACGGATGGTTTTGTTGTGATCTGTA<br>GATTAAAAATCTGGCAGCTGAGGTTTGTCTTGACAGGGCTTATGTTCTTGAATTGCT<br>TCGTGACCCGCCTCCAAAGCTGCTAATGCTAAGTGCTACACTACCAGATGAAAAAC<br>CGCCAGTAGCAGCACCTGAGAACTCCTCACCTGATCCAAGTCCTGTGGAATCATTAT<br>CAGCTGAAGATGTTGTGGTCGAACCTAAGGAAAAAGTAAAAGATGAAGCAGTTCAT<br>GTGATGCAACAGAGATGGTCTGCTCAGAAAAGAGTGAAGAAAGCTCACATTGAAA<br>CGCTAGAGAAAGTTTACAGAAGATCAAAACGACCCACTGTAAGGATTCTCCTTTTA<br>CATTTGAATCAATTTCTATGTTACTTGAATGCTCTATCTCACATATGATCATGTTTGA<br>TGATGCTGTGAATAGAATGCTGTGGTTAGCAGCATTGTTCAAGTGACCAATCTTCCA<br>AGGAAGCGAGTTTTGAAGTGGTTCGAAGATAAAAGAGCAGAAGACGGAGTTCCAG<br>ATAAGCGAGCTCCATATCAAGCTCCGGTTTGATCTAATGTTAACGTTGAGATGGCAA<br>TGATTTGTATACTTGATTCTCAGAAACTCATCAACATTGTCGTCAAGGACAAGTTTT<br>TTTGGTGATACGAGGAGTGTTTATAGTAGTAGATTCTGTCCAATGGTGTGGCTGGAT<br>ATGTTGGACTATGAAATTTTAGGATATCTTGTATTCAGTTTTTAGTTATTTCCTTGCT<br>GAGATTGTGTCTTGTAGAAAACCGTTTCAACTTTGTTTGGTTTATGGCGGCTATAAA<br>GTTTAATTTTAATGCATGACAAAAACAAATCACCAAAAATAAAATAAATTACTTTC<br>ACGACACTTTTGAAAGCACTGCCCTAGGCGTGGGCCATGTGACAGAATGAAAGAAC | | |

| | | | |
|---|---|---|---|
| | TCAGACCAAACTTTTCTGTCCAAGGACAGGAATGGGGCCCACCCAATTAGCTCCCCT<br>ATCCATTATTCACCGTAAGATGCTAACCAGATCTAACGGCTAAAATCCACCCACGTT<br>CCAATCTCAATTGCCTTTGGATCCTTGTATTTCCTCAAGGCTCACCTTTCTCCACGAT<br>TCACTCTCGATATCCGTTCGATTCTTCAGAGATCTGACGGCGGTAGCCAGAGTAATC<br>TATTCCTTCCCAAAATGTCTCGCAATTAGATTCTTTCCAAGTTCTTCTGTAAATCCCA<br>AGTCCCGCTCTTTTCCTCTTTATCCTTTTCACCAGCTTCGCTACTAAGACAACAAATC<br>TTTCCCTCTCTCTCTCGCCTGATCGATCTTCAAAGAGTAAGAAAA | | |
| P_CasP1 | *Arabidopsis* | 2088 bp | Naseer, S., et al., (2012). Proc Natl Acad Sci USA 109:10101-10106. |
| | GGAGAATAACGCATGCATTGTATTTGTATTCAAAAAAAAAATATTAACACAACATG<br>AGAATTGGCGATTAAAGATTTTGACCTAGATAACGTTCATTATCAAAATTCCAAACA<br>ACTAAAAGAAAAGACATTGATTATTTTCAAGGAAAAGCTCTTTCCTCAGAAACATA<br>CCCCAACAAAATAAAGTTTCGAGCTCTCTTTCCAGATATGTTCCTTAACATTCGAGC<br>TCTCTTTCCAGATACGTTCTTTAACATTCGAGCGCTCTTTCCAGATACATTCCTTAAC<br>ACCGAGCTTGCAGCAAACATAGACATCATCTGACGCCTGAACAATCTGATCATGGG<br>AAAGGCTACGAGCACCCCAATTACTCATACATATCTCCTTATCTTTCCTCACTCCCTT<br>GTACCCCAAACACTCCTCTACAATCTTCTCAAACGAACTATTGAGGAGCCTCGTAGG<br>CAGATAGTGCCTTATGTCTAGAAGTCTCCATATCTCCAACTGATGGCGGAATCTTTC<br>GAGCTTGCCCTGGTCTTGGCTGTTCCAGACGCCGACAAAAGTGATTGTCTCATCTTC<br>CAAGAAACTTCGAAGGACCTCAGGAATGCGTTTACAGTGAGACAACTGGATGATGA<br>GACAGCGGTTACCAACACATAGTTGGAGAATATCCGGTGGAGGATCGGAACCACCG<br>GGTGTCCATTGGACGTCGAGTCCAACAACGAGAGGGTGTGAGGAGCGAAGACGGC<br>TGAAGAAACGGATGCTATGAATCCAACGGCGGATGGTTGAGGTAGTATGAGTGACC<br>GTGACGATCAAACGTTCTCCAAAGAAATCGATGTAGCGGTTTTCGTGGATATGGCG<br>CTTTTGGATTCTTCTTCTGATCCTAGCCATTTAATCTGCATAAAAGTGAGTATGAGA<br>GAGAAGATTAAATAGATATCAATCCTAACTAATATTCAAGAAAACATAATATAGAT<br>CAATAAATTGATGAGAGTAAAAACACAAAGATGTTTAGAAATAATTATTGTCAAGA<br>CTCAAGTTTCTTCAAAATATCAAGAGGCGCTTGGAATAAGACCCTTATTCTACAATA<br>CATCAATCTATATAGAGATAAAGACTAAGCATAATTTTTAAAATAGAAAAAATATA<br>AACGTAAATAACACTTTTTTGAGGTAATACTAAATTTTCTAAACATGAAATGTTACA<br>AATCCACAATATTTCCATATAAATTTGTAAATAATATTTTGTTAGATAATGTTAAAT<br>TTTCTAAACTGAAATATTAACAAATCCGCAGTATTTCCATTATTAAATCTCGATTTTG<br>TTTCAATGGGAGATTTGAATTTTGAACCAAAAAAAAAAAAAAAGATTTCATCAAGA<br>TATCTAGGGGGATATTTTGCTGGAATATAGCTTTGATGAGAATATTTATATTTTGTA<br>TCTCTGAAAATCAAGTTTAAAGGGGAAATGATTATGGGTTGAAATTTTGCAATCAA<br>AAGCCCTAATTTGCAAAAACTACATAAGTTTTTTGTTTGGGCTGGCGCTATCGGATC<br>CTTTTAGGCTTACATTTAACATCTGGTCCACTTAGAAAGAGTCACGTAGTATATGGT<br>AATTGTCAACTTGATTTTTCAAGTTAAAAGAAATATGTATCAAAATGACTAAAAAGT<br>AGTGAAATATTATGTATCTAATTTGTTTATTTACCAAATTAATGCTATAAAAATGTT<br>CAACTGTACAATTGGCATGGAATAATATGAACATAAATCATACATTATTAAGCACTT<br>TTGCCTACGAAGGGATACCAACTTCATTAGTTTACATTTTCTTTTGTGTTCAATTGTT<br>AGCTCAAACCCAATTAAGTGGGGAAAGTAAGAAGCAACAACTCCTCTTCCCGGACC<br>CCTAACAAATCAACTAAACTCAATATCAAACCATTTTAAAAGAGCTCATCATTAACT<br>AGCTACTAATTATTCTTAATCAATCACTGCTTAATACAAAGCACTATATATACACTT | | |

| | | | |
|---|---|---|---|
| | GTATCTTCCATTAGTTTCCCACCACAACTACAAAACATTCCAATACACAACACACAA<br>AGCACACACTTTTTCTTTCTTTTAAACCCCAATTGCAAGAGAA | | |
| P_DAISY | *Arabidopsis* | 2047 bp | Naseer, S., et al., (2012). Proc Natl Acad Sci USA 109:10101-10106. |
| | GGAGATTAAATGCTCGCTTTGATTGATGCAAACAATTTCTTCACTATAATAAGCGAA<br>AATAATAGAAATAATAAAGATGGAAAACTAACAATTAATAGTAGAACTATATATAT<br>ATATATCCACATATAAATTCTTTTTTTTTTGAGGGGTGGTTTAAAACTAGCTATTTA<br>GCTGGATGCTCGGCTATTCGGATTTGTGTAAAATACTTCTAAATATTGAGCAATAGG<br>TAAATATAGTGAATATTTAACCATGACAAACAATAAAACACACACACATCAGAGGA<br>AACTTGGGAATGAACGTGAACAATAAAAAAAAAATTAATAATGATAATAATAGTG<br>GGCATAGAGATGGTTCTTCAACGAATTATTTCTTGCCCAAGTAGCACCGTTTGGTTA<br>GAAATTCTTCGACATTTATAGTTCAATTAAAAATTACTACTCCATAATTTTGGGTGA<br>CTTACCACACAATCTTTCCACAAATCTTCGACACGCAATTCACAACAGTATCATAGT<br>TTATTATCACACTTTAGTAATTTATGCCAAACCTCACTACGCAAGCAAACTATGATT<br>CATTAGATGTGTGGACAATTACGACGACAGCAAAAAAATAATATGTGTGGACACAC<br>AATGACAAACTCACATTGAACTATCTTGAAATAAATTTATACTAAATCGTAGCGAAT<br>ATATAATCAGTAAATAGAAGTTAAACTGAAGATCATAATCAACTAAAATGAATATC<br>GTAATCAAATTGATGATAATAATGATTTCAGACACAATAATGGTGACGGTGTTGAT<br>GATGAAAAGGATCTGTGGCATAACAACTGATTGAATTCAATGCATCTTCATGGGTTT<br>TATTCCGTTAGCTTTATCTTTCACGTCTTTATATTCTTTAAACTTTGTTATGTTATTGA<br>CATTTAAATTCATTTGTATGCATGTTATGAAGAGATCGTATAACTACAAAAATATAA<br>AGGTAAATCTTTCATTTTAAAAAGAAAATGTTTCTATACGTGTAAATAATTAGTAAA<br>TAACACATATTTCCTCCTGCGCGTAGCAGTTCAACAAACTCTCTTCTTCGCGTAGCT<br>AGAAGCTGCAATAACATAAAGAAATGATATATTTATAAATTATGTGAAAATGAAAA<br>AAAATATTAGTCCGCAATATCCACGTATCATTATGCGCCACGTAAACAAAGTGGAC<br>CCAGAATAACCCTTGTTTCCAGTAGAAGGAATCAATCAGTGGCAAGAAAGAAAATA<br>ATGAATTTCATTAAATCGCCACTCTCGAGCCATCAAAGTATCAAACAATTGATTGGA<br>GTTAGTTGTATCCTTCGCCTACAAATTTTTCCTTACTCTGTTTTATAAGTATATAATA<br>GTTCCACTAAAATCATTTTGGATACAACCTCAATAGATCTCAGCGTATTGTTTCCTG<br>TATGTTCAATGAATCCATCCTCCATGCAATCTGGAGACTGGAGTATAAAATAACAC<br>GTATGGTATGGTATTTATTTCTCATAGCTAGTTCCTTAACAGTTGGAAGAATTTATA<br>CATGACCCGGCCCTTTAAAACCTATCCCTACTACAAGACATTTGGGTTACTTTCCAT<br>CGACTTTTGAATGTGCATCAGGCATGAAAATCCTTTAATTATGCATATTTTATAATC<br>AATGCTTAAATTACTATAAACACAAAAATTGTAGTGATATATTAGAAAGATAATTT<br>AAATTGTTACATTGAAAAGATAATAAAAAATTATAAATAGACATCTGATATAAAAA<br>TGGATGAAGTATAGCATATTAAAAAAACATATGTTTTTGGTCAAAACAGAATCAAT<br>GCATAGTTAGCTCACCGCTACAACAATAACCATAGGGACTACGTACCATCCATAAC<br>TACATTTTCTTAAATTGCATCCTCTTTCTAAAATTTTGCCTATAAATTCACAATAACA<br>CTTCAACTTTTTAGGCCATAAGTTATCTCTTTCTCTACAATAAGCAATAAATCTCACC<br>TCCCTTTTTTTTTTTTTTGTCTCGCTACTTTTGATTATCATTTAAAACCAAAAAACCTA<br>CC | | |
| P_KCR1 | *Arabidopsis* | 2223 bp | Naseer, S., et al., (2012). Proc Natl Acad Sci USA 109:10101-10106. |

| | | | |
|---|---|---|---|
| | GGAGCCAAGAAAGTTGGAAGAGGAAGTTTTGGGTCTGTCTACTATGGTAGGATGAA<br>AGATGGGAAAGAAGTTGCAGTTAAGATAACTGCAGATCCTTCTAGCCACTTGAACA<br>GACAATTTGTGACTGAGGTATTGTCTAATTTGCAGCCATTAACTTGCTGCAAAGTTT<br>CTTAATTAGACGATTGAATTAAACCAAATGTGCAGGTTGCTCTATTATCGAGAATTC<br>ACCACAGGAACTTGGTTCCTTTGATTGGATATTGTGAAGAAGCAGATCGGCGTATTT<br>TGGTTTACGAATATATGCATAATGGAAGCTTGGGAGATCACTTGCATGGTATTTACC<br>AATTTCCCTTATATTTTCCTGCCTAAGTTACGGATTTCATTCTGTGAATAACTCATGA<br>AATCCATCCTGTTAGGTTCAAGCGACTACAAGCCACTAGACTGGCTAACTCGGCTAC<br>AAATTGCACAAGATGCTGCCAAAGGTTTGTAGCACAATCTCAAGATTAAATCTTTA<br>GTGTGGCTATAATATGAATCCATTGGACAGACAAAACTTCAGATTATTACAGGTCTT<br>GAATACTTGCATACTGGCTGCAATCCTAGTATTATTCATAGGGATGTGAAATCAAGT<br>AATATTCTCCTGGACATTAACATGAGAGCCAAAGTATCTGACTTTGGACTCTCGAGA<br>CAGACAGAGGAAGACTTAACTCATGTATCCAGTGTCGCAAAAGGAACTGTTGGATA<br>CCTTGATCCAGAGTAAGTCTAACAACACAACCTCACAAAAGTATCCCTTTTTATGAA<br>CTTTGACTGATAGATGCATTCTTTTGACAGGTATTATGCTAGTCAGCAGCTGACTGA<br>GAAGAGCGATGTCTACAGTTTTGGCGTTGTTCTTTTTGAATTACTCTCTGGAAAGAA<br>GCCAGTCTCAGCAGAAGATTTTGGTCCTGAACTGAATATAGTTCACTGGGTAAACA<br>CCTCTAATCTTCTGTCCATTTTCACCTTTGACCGTTTAAAGTACTACATGAACAACAT<br>CAAGATTCACAAACCACATATCCTCTCTATGCTTTTCTTGTTCACCAACAGGCAAGA<br>TCGCTGATCCGTAAAGGAGACGTGTGCGGGATCATAGATCCATGTATAGCAAGCAA<br>CGTGAAGATCGAGTCAGTCTGGAGAGTTGCAGAGGTCGCAAACCAATGTGTTGAGC<br>AGCGTGGACATAACAGGCCAAGGATGCAGGAAGTAATAGTGGCAATACAAGACGC<br>GATCAGGATCGAGAGAGGAAACGAAAATGGACTGAAGTCATCGAGTTCAAGCAGC<br>TCAAAGGCACAATCATCTCGTAAAACCTTGCTTACTAGCTTCCTCGAGCTAGAGAGC<br>CCTGACATCTCAAGAAACAGCCTAGCTCCAGCTGCTAGGTGAGTCCTCTTAAAGCTC<br>GAGCTCTCATGTTTACCCTTTGGACTTACCATCGTCACATAGATGAATACATTATAT<br>ACGTATTAAAATGTGTGTATGTGATGTAGTGTCAAATTTAATCTGTATCTCTTTACA<br>CACTTCTGACATGACATTGTGTAGATGTTTTAGTTGATGATAGCACAAAACCATAAT<br>TTAATTATTGAAGGTAACAACATAATATGCTATCTATCTCCTATGCTGATGAAGACG<br>ACAAATTTCCAAAAACAATTCTTGCATGTCTTGTGTCAGGGAGTAGCCAATTGTCAA<br>AAGACTCGAAACAAAATGATACAAATGCACCACTCATAATTTCACGATCAACCATT<br>TGTCACAAAACCTCAAGAAATCTAAAATTGTGTCTAAAACAATGTCACACTGTTTCC<br>TATGTTTTGTTTACCACTTGGTTTCCTCCTCTATTCCGCTTTTACCATATGAAACCTTG<br>TTTTGTATTGCACAATTCACCATGAAAGCCTAAATTTACCATTTTTGGCAAGTAAAA<br>TAATCTGTCAACATCAGAAGGTGCCAACTTCAATACTCAAACATCACATGAAACCA<br>AATTTTAAGGAAGAAGCCAACACAAAGACAATAAAATTAATCAAAAATAAACAAA<br>GGAGAGAGACAGAAGAAACAGAGAGAGTAGTTAATTCCGGTGGTTTTCTGCTCATC<br>TACTTCTATCCACCTTCGTCCCTTCCGCCCCAAAATTCAATTCTCTCTTCCTTTCTCTC<br>TCTTTCTCCTTAAAAACTCTAACTTTACTCATCTCATCTCCAAAGTCTCAACCTTTCT<br>TCTCT | | |
| P_HORST | *Arabidopsis* | 2055 bp | Shih, P.M., et al., (2016). Nat Commun 7:13215. |
| | GGAGTATGCATCGATGATGATTCTGCCTCACTTAGAAGAATCAAAAAGCTAAAAGG<br>AGCTGCGAATCCTGGGCAACGATGACTTAGTATGTATAGAAGAAAAAAAAAGCTTT<br>TACTTGGTATCAAACAGAGCATGTGTTGTTGTATTTGCTACCTTTTTCTGCAAAGTTA | | |

| | | | |
|---|---|---|---|
| | TTGGCAATTGTATTGTTGTTCCACTTGTATATATACAGAGACCATTTATGTTAATATA<br>CAACTTCGTATGGGGAAAGATGATTCCATCAAGTCCAAAATGCCAAAACCATGCAC<br>GATCTTGTCTGATCAAGTATGATAAGATGTGAGATTTTAAGAACGTGAAGCTTTTCA<br>TATTGTATTCTCAATGAACGAAGAATTAATAATTAGAGTTAGAAATGCAGATTACA<br>GAGATTACACAATTTGATCTCATTTCTTGGCAAAGAGGCCCAGGCCACCGAAGCCA<br>CCGTCCTTCTTTGGATTCCTGTGTGTTAATTTTCGTGTGGCTTTAGTTACAAAGGGAT<br>TATATTGTCAGCGAATCTGAGCCGTTGGATCGTTTTGACATGAGCTTTCCATAAGTG<br>TCTTATCCTTTGTTTCTCTCATTTTTCACATATGTATCAGACATTTAATCAAGCACAT<br>ATCTACGTGTTGATTATGTTGATGATGCTGAGATTATGTTGATTTAGAGAATGAGTA<br>TTGGTGATGATGGTACACGTTAGTAAACATATGAATTTGGAAGTGCCACGTGAGAG<br>GGAGCAGAGTTTTGGGCTTTCTTTTGGTTATGTATTTGCATTCTTGTTGTAACAAATT<br>CTCTTATTAAAAGCATTGTGAAAAAAACCAAATACCACATAGTCTATGTTTCAAGAT<br>TTGTTTTTTAATACTATAATTTTCTATTACTTTAGTTTGTGTAATTTTTGAAAAGAGA<br>AAATTGGTATTTTTTAGGATTGGGTGATCTGAAATTTGACTACAAAAATTTTCTTGG<br>ACACAGACACACAGTCACACAAATAAGCTAATTATTGGAATTTTATCTTAAGTTGCA<br>TTCACTTTGCAAATTAATATTGAATTATAGACTATTAGTGTATGGCGTGATGGCAAA<br>CAAAAAAATCAGTGTGTGATATTGTGGACTAGCCAAAAAAATTTTCTGATGATTTTT<br>TTGCCTAAATAATTTGAATTGTTGAGACCGAAACTCTTACTAATATACTAACAATGT<br>AACGCGACAAATAAAACATTGTTGAGAATAAAATCTGAACCATCATAACTATAGTC<br>TATAGTAACCGTCCGAATTCAACCACATATGTATGATCTTTGCCATTTTTGATCATAT<br>TTTCCGTTGCGTTTTCTTTGAATTTTCTCAATATATTTTTAGTGTTTTGATTTGTACTT<br>TTATCTAATTTATATAATTGTTTTTCAAATGTTTCGCAATTGTGCGTTTAAGTATTCG<br>ACTCGAAGTAAACCCAGTTTCTTAGAATGGGCTAACTGCCATTGACCAAATCTTTAT<br>CCGCGAAGAAAGATCTTTAGCAGCTAGGCTTATTTCATATCAACAAAAGTATATATT<br>TATCTCTAACTAAATTCCTACAATTATTTCAAAAACATAGAAATTAAAATTTGAAAT<br>GCCTATTCTTTTTTGGTTATACATTGCAAAGGTTATGTATGTAATTTCTGATCTTGAA<br>ATTTAAGTTCTTTAGCCCATATCTAGTTGATTTTCACGAAATATTGTCGTTTACTCTC<br>GTTTTTATATATAAAGCATGTACATATAACATATAATCAATGCTTCAAAAGAAAAA<br>AATAATCATAACTAAATTGAAACGGGAAAACTAATTTGCTTTCGTGTTCTATTATGC<br>CAAACTGTACTCACAATAGTCTTATCCTATCGACCCAAATTTAATCTTCCCAACTAC<br>TTACTCCTAATTGAAACAAAATATAACCTTTCTTACTATAGCCTATATTTAAAAAAT<br>ATCTATGATTTCTACATTTATATATATACGCACACGGCACATACCTCTCATTCACCAT<br>ATATGCCTCTTAACAATCTCTCCACAGAACAAAAGCAAAAAGCCTAAACCGGGAT | | |
| P_GPAT5 | *Arabidopsis* | 2153 bp | Shih, P.M., et al., (2016). Nat Commun 7:13215. |
| | TGATCGCAAACGTCAATGGTCTATATCCATCTCTTGGGGATACACCATTCAGATATA<br>CACTTACTTCTTGACTGCTACGGAGCTGACTACGCCGTTGCAGACCTTCAAAACTTG<br>GCGGTCTTCCAGCGATGGACCTTTCGTGTTTAACACTCGGCCCTTGAAACCTGACCC<br>CTGTGAGCGTCCGGTCACTTATTTCATGGATGGTGCCGAGGATGTACGGGATAGTG<br>GGACGAAAACTTGGTATAGCATAGGGGATAAGAACTATGGTCATTGCGGAAAGATT<br>GAGCATACTCGATTAACTAAAGTGAAGAGGATATTGGTGACTTCAATGAAGACGGA<br>TCCTGAGTATTGGAACAAGGTACTTAGTGTTTGTATATTTCTCATATTGAATTTAAC<br>ATTTTGGGTGAGTTTAGGTTACATCTTTTTTTTTATTTTTTGCTTTTGTTGATAGGCAC<br>CGAGGAGACAGTGTTGTGAGGTGATGGAAGGAAAAGTAGGAAAAAGAAAAGAGA<br>AAGAAATGTTGTTAAGGATTAGAAAATGCAGATCTTTGGAGAAGATATAAAAAAAG | | |

| | | | | |
|---|---|---|---|---|
| | CGTTTTAATTAGAGAGATTTTTGCACATACAGATTTAGATTCGATCATTAGTTATTTG<br>TTTCTAAATTCCTCAATTCCTAGTACCAAGAGAATGACGATTTTCTCTAGTAATACT<br>GGCACTCTTCGTTTTTCAACATGTAAACATTACTTTGTATAATTAGTCTATATCAAAT<br>AAATAGTTACAACATGAACGTAACTATTTATACACGAGGGTAAATTTTTAACAAATT<br>TAAACCGAACCAAAAAGATTTCAGTTAATCATAGTTTTGTTACAAGCGAAATCTGA<br>AATATTCACCCTCAAGTTTGTTACGTAATTGTCTAATCTGTGTTTTTTTGTGCTAGTG<br>TTATAATTGTTTCCTCGAATGTTAAGCACCACATAGAAGTATACACTTTTCTTTTATT<br>ATTTTCGTTCTTCTTCCATTTAGAAACTTTTATTAATTCTACTGTTAAATCCAACGTT<br>ATTAGTTTATTTTCTCTGATGAAGTTCTAATTTTTTTTGTTTGTTGGAAAAAGGGTTA<br>CTTTTCTGATGAAGTTGCCGATTTGTTTCCAACCAAACCTCAGCTAATATAGTTAGG<br>GAGACGGATAAGAAATTGCATAAAGGGAGAGGCTTTGTAAACACGCAAGTTTACGT<br>CAACTTGACACATATAAACTCGTATATAGGAATTGTGTAAAATGTTAATCAATATAT<br>GTTTATGAATATAGTCCTTTTATTTCCTTAATCTATAGTCATGTTATCATCATATCGG<br>CAAGAAAATTACTATTACAACATATGTTTCCGTGCTTCGATTTGTCGATCAGGTAGG<br>CAACCAAACCGGGCCAGATTAGTCACAAATGAAATTAGGCTCCACTTTGGCAAGTT<br>TTTTCACAATTAAAAATTAGAAAGTCTAACCAAACTAATAGAAACTGAACCAAACA<br>AAAATGTTCACGCAATTACGTAGCACTTCTCTATGAAATACTGTGGATAATTCGATT<br>TGAGCTAATCTATGGTAAGTCGTTTCTCTCAAACTGAATTAAGCTAAACCAAACCGA<br>AAATAAACCGTTGTAGAACACCCATAATAAACATATATATGTTCTCTACTCTTATTT<br>GGCTATTTTTCCATTTGCAGATACGTGTGACAACATTCCAAATATAGGCACATGATA<br>CAAATTACAAAGCCAGAAATTATAATTTATAAACTATACATAATAAGGTCAGTAGC<br>CTAATTACATCCATATATTTAGTTGGGCATAATAAGGATAGCCTAATTTTGGTGCAT<br>CCCGCCAAAAATTCGGACAAATGGTGAATTTCAAACCATGAATTTCTTTGTTATACA<br>TACATTACGTGCACTATTACATCAAAGAAAAATAGTGACGTGCATAAACGTATTTTC<br>TTCACATAATCTATATATAATATATACAGCCATGTACATTTTTCAACATAGAAAAAT<br>CATGTATACAAATACGGTAGGTGCAATACTGATAAACTGCCATTCCCATTGCATTAG<br>ACGCTAGTCAATCCAAACCTTATAACATAAAAAGTTATAGTCCATTTCTTTCATATC<br>CAATCGAGTCAAAATAATATTCGAGCAAAAAACAAAAGA | | | |
| P_ASTF | *Arabidopsis* | 2081 bp | Shih, P.M., et al., (2016). Nat Commun 7:13215. | |
| | GGAGCAGGGTTCTTCATCATCATTCACATGAAGATCAGCAGCAGAGTGCAGAGGAA<br>AATGGTTCATAAAGCTCAAACCTTTAAATGGCTTTTAATTAGATATATTTAGCGAGA<br>ATGTGATTGCTAGTGAAATCTCAAACTTGATAGCTTATTCATAGCTTATTTTACTTCG<br>ATCAGCAAGAGAACAATGGTTCCTAAAGCTCAAACCTTTTTTGGCGTTTGCTTTAGA<br>TTGTGCATACATAAAGTGAGAAGATGAAATATGGTTTTGCTGAAATCTCAATGTGCT<br>CATTCATAGTTTATTTTGCTCGACCTTTCTTTAATCAATAGCTTTGATGTACTGATTA<br>CTTTATAAAGTTAAGAAGTAAGTTCTGCTCTTTAAACTGTACTGAATGGAGGCTTCT<br>CTGCATAATTAAGCAAACACTCTGATGATTGAGTTTCGTTGTACAAAGGAAAGTTTT<br>TGCCTTAGTTAGAAATTCATCTTGTTTCTTTGCCCAGAAGCTGGCTTGCTGATAGAA<br>TTTGGACTACGCTACGTTTTCTTGGGTGAAAAACAATCCGCGTCCTTCCACGAGAAC<br>AGTGAAACTGTACGATGTTCTTATTGTATTGTGTCAACAGCCCATAATCAATGAAAT<br>GGGCCTCAATGACAGTATAAGCCGAAAAATATTCGAATGCGATCTAGAACTAATCT<br>GAAACCAAACTTCTCAAATCATTAACATTTGATTAGGGCCTAAAAGTCCATCCGTAT<br>TTTAAAAAAAAGTTGATAACAAATGCAAAAATACAAATATACATTACTATTAAAAC<br>TCAATCTTTCTGATTATATGTGGATGGAGAAGCTGTTTTGCAGTGTGGATTTGAATT | | | |

| | | | |
|---|---|---|---|
| | TTGAAGTTCTTGTTCAAAAAAGAAAAGAAACTTGGTCAAGCTTCAAAAGAAAGAAA<br>GAAATAAAGACAAGTATACAAATACGTTGACTTCAATTAATATGAAGATAGAAGAG<br>AAAGATATAGAAATGCGTTTTAAATTGGCGGTGGGAGATATGTGATTCCCTTGGTCC<br>CGCTTTCTCGTTTGATTCGGTAACCTAAACCTTCTCCATCCAAGAAGACACAAACAA<br>GGAAGAAATTAACCTACGTACGTACATCATTTGAACTACTTATATCTATTATAAATG<br>GAAAATCTATTTTCTTCTTCTCTCTCTTTTAACCGAAAAAGGAAAAGTCCTCGAAAT<br>AACTCTTTAATATAATTCATGTGGAAACCTTCTATATATTTTTTGCATGCTTTTTTGA<br>AATTAAGTAATGGCATACTAATTAGAAGAAGACGAAAAATAATATGTCTAAGTAAT<br>TGAGTTTCGTGGTCAAGGCAAATCAAGATTGCATTCTTTAATTAAGTGTACAAATTA<br>TGATGTAAGAACCGTTATTTGGCATAACCTAATATATATTAAAAACTGATTTTTTTTT<br>TTTTTAACTTCTGTAAGAAATGGTTTTCTTAATTACCAATCATGATCTATTATTGTAA<br>AATGTGTAATTTTGTGTTGCGATTGAAGTGTTCTTAGATAAAGATATGTCATATGCA<br>TGATATCATCTATTTGATTTTGAGAAAATAAAAACATAACATGAGAACATCGTAGA<br>GAGTAAGATACATGACAAAGAGAAAGAATAAGCTAAGAAGTCATCCAAAAATTGG<br>GAGGCCTACTCTCAACTACCAAAAATACAAGTGTTTGTTGTGTTAGGTCATTTATTC<br>AGTCACCTAACCCTAACTATATGAAATACGTTAAAATTCATATATAGAAAAATAAA<br>GAAGAAATACTATTAGTCAATATAATTTTTTTTAACAAAATAAAAATAAAGAAAGA<br>AACACTATTAGTCAATGAATTAAAAAAAAAAAAATCCATCTTTCGATCAAGACCGT<br>CCAAGTATTCTCCTTTCACATAACTCTTACGTTTTACACTAAACAAGACGAGATTTTT<br>ATGAGCTTGTGGCTCCCACAACAATCCTCATTGATTTCTCCATATATATCACATGAG<br>AATCCAACTCGACTTCATACTAACATTCATCCCAAAAGTTCCATTCTTTTTCACAAA<br>GCCATTGTGCTGTTTTCTCCATTTGGATCAA | | |
| P_FAR4 | *Arabidopsis* | 2065 bp | Shih, P.M., et al., (2016). Nat Commun 7:13215. |
| | GGAGAGTTCTAACCCCGACATAAGGGATAGGGCAGCGGTGTATGGACCTCATATTT<br>AATATTAAAAAATTATAAAAAAATTGTTAGTGGTATAAAATACTCTCGAAAATATA<br>AATACATTTTTTTTGTCACAATAATACATATTTATATTTGCAAATATAGAATTTTAT<br>TTAAATAACAAGACTAAAATCTTCTTTTTTTTCCCTAAGGCTCTTTTTACAGTTGGGC<br>CGGGGCTGATCAGTTCGCTATTGAAGTATACATGTCAAAGAAAATGAAGGAGAATA<br>AACCATCGTATTAATTAAATCGATTTGACTAATGAGACAACTTAATTCAGCTTTAAT<br>ATAGTTTAAATTTCGTACATGACCAGGTAATCAAATTTTCTAAACAAATGGAACAAT<br>ATCAGATAGGGTAAACTGTTACCATCTGTGTAACTTTGCAAATTAGTAAGTCTAAAT<br>TATAAGAAACGATGACTTGTACTTCTAAAACTCCAGCATATCATATAAAATTTTGAG<br>GTTTGAGACATTTCTATAAGTTCAGATTTAATTCTTCCTAACGTATGAGGTTAGGTA<br>TTTTCTGTCGCTTTACCAAAAAATTTGACGTTTCCCATACGTTTTAGCTTGTTATGTC<br>ATTGTTGCATAATTTATGACCAACCAAACCTAAGACCAAATCAGGTTTTTAGATAGT<br>CCTTTTTTCCTTTGAAATTATAAGATGTTTTGGTTGTATTGGCATAGATTACAAAAAT<br>TACTCCCCCCATTTTAGATTAGCTGTCGTTGAAGATTTTTACAGAATAATAAAAAAA<br>CATTCAATTTTTCTGTTTTACCCTAATTAATGTATTGTTATATTTGATATCATCAATA<br>AATACGTTTAAAAATGTTGATAAAATTGGAAACTTTGTCAAACATCTGCATTGTAAT<br>ATATAATCTAAAAAAAAATGATTTCTTATATTCTATCAAATATGGCTAACTATACACA<br>TATAATTTGGGCTGAGCTGTCATCTTTCGACATGAAATTACTCCGAATTATACATAA<br>TTTGCCAGAGAAGTTATGTTACATAACAACACTCATATTCAAAAGAAAACTAGATG<br>TATAATGTATTAGCTATTATTTAAGTAACACTAACAACTAAATAAAATAGAATGGAT<br>GAAACTATAAAAAATTAAGAATTATTATTGAATGAGTATATTTTTTTCTATTTTGGT | | |

| | | | |
|---|---|---|---|
| | CAAACGTCGAGGTGGAGTTTAAGAAAATCGTGATTCTAAAATAGTGCAATAATGAA ATATTAAAAAATCACGTTATCATCTTGTTCTTTTATTTTCCACTATTTTCTCTTGAAA TTTAAATGAAATATGTTATTTTCCAAAATGTTGTTACAACCGATTTACTATCTCATAA ATATGTTCTTTTGTGATTTTAATTTTTGTATTATTAATGTTCCCCTGCTTTTTGTTGAC TAAACAACTCGTTACTCGTTAGATATATATTCACGTTGAGAAAAAAGCGTTTAACCA AAACACTTAGGTATATTGAAGAGTTCTAAAATTAGAACGCCAACCTTCCAAATCAA CACACCTACGACAATTCTAGTCCTACAACATAGATCCTTCTAGCAAACCACAAGGA AACTTCTTGTGGAAAACACTAACACTATCCTCACTCAGTTACTCTCGTATTGCTCTA CCTAGAGTTTCTAATTCTGCTATGTTATAAAAATTCTTACATACTTATTCTTTTTTTC TTTCTAAACATTCCATTCGTTTTAAAAGCTAGGCCCACGATTAGTAGTTAAAATATT TTAATTTTTAAAAATATGGGTTACATATTTTTTATTCTTTTTAGAGCTCCGAGATTTC AGAGCATGTTCTAATTGGATATGTGATATTTAATCAGAAGTAGTTTTAATATTGTTT TTTTTTTATAAAAGAAAATGTAGGTAGGTTTACGTACAAGTAGTAGATATGATGGCT CAACAAAACGGGTCAACATTTACGGGTCTATATATATTCGTGCCTCATCGCCGAAG GTGTTATGGTTCAAAACAATCTATCGTACTTAGTTATTAATTGGATAGATATAAGTT TCTTC | | |
| P_AtIRX5 | *Arabidopsis* | 1990 bp | Gondolf, V.M., et al., (2014). BMC Plant Biol 14:344. |
| | GGAGATGAAGCCATCCTCTACCTCGGAAAAACTTGTTGCGAGAAGAAGACATGCGA TGGCATGGATGCTTGGATCTTTGACATTGATGACACTCTTCTCTCAACCATTCCTTAC CACAAGAGCAACGGTTGTTTCGGGTAAATAAACTAAACTTAACCATATACATTAGC CTTGATTCGGTTTTTGGTTTGATTTATGGATATTAAAGATCCGAATTATATTTGAACA AAAAAAAATGATTATGTCACATAAAAAAAAATTGGCTTGAATTTTGGTTTAGATGG GTTTAAATGTCTACCTCTAATCATTTCATTTGTTTCTGGTTAGCTTTAATTCGGTTTA GAATGAAACCGGGATTGACATGTTACATTGATTTGAAACAGTGGTGAGCAACTGAA CACGACCAAGTTCGAGGAATGGCAAAATTCGGGCAAGGCACCAGCGGTTCCACACA TGGTGAAGTTGTACCATGAGATCAGAGAGAGAGAGGTTTCAAGATCTTTTTGATCTCTT CTCGTAAAGAGTATCTCAGATCTGCCACCGTCGAAAATCTTATTGAAGCCGGTTACC ACAGCTGGTCTAACCTCCTTCTGAGGTTCGAATCATATTTAATAACCGCATTAAACC GAAATTTAAATTCTAATTTCACCAAATCAAAAAGTAAAACTAGAACACTTCAGATA AATTTTGTCGTTCTGTTGACTTCATTTATTCTCTAAACACAAAGAACTATAGACCAT AATCGAAATAAAAACCCTAAAAACCAAATTTATCTATTTAAAACAAACATTAGCTA TTTGAGTTTCTTTTAGGTAAGTTATTTAAGGTTTTGGAGACTTTAAGATGTTTTCAGC ATTTATGGTTGTGTCATTAATTTGTTTAGTTTAGTAAAGAAAGAAAAGATAGTAATT AAAGAGTTGGTTGTGAAATCATATTTAAAACATTAATAGGTATTTATGTCTAATTTG GGGACAAAATAGTGGAATTCTTTATCATATCTAGCTAGTTCTTATCGAGTTTGAACT CGGGTTATGATTATGTTACATGCATTGGTCCATATAAATCTATGAGCAATCAATATA ATTCGAGCATTTTGGTATAACATAATGAGCCAAGTATAACAAAAGTATCAAACCTA TGCAGGGGAGAAGATGATGAAAAGAAGAGTGTGAGCCAATACAAAGCAGATTTGA GGACATGGCTTACAAGTCTTGGGTACAGAGTTTGGGGAGTGATGGGTGCACAATGG AACAGCTTCTCTGGTTGTCCAGTTCCCAAGAGAACCTTCAAGCTCCCTAACTCCATC TACTATGTCGCCTGATTAAATCTTATTTACTAACAAAACAATAAGATCAGAGTTTCA TTCTGATTCTTGAGTCTTTTTTTTCTCTCTCCCTCTTTTCATTTCTGGTTTATATAACC AATTCAAATGCTTATGATCCATGCATGAACCATGATCATCTTTGTGTTTTTTTTTCCT TCTGTATTACCATTTTGGGCCTTTGTGAAATTGATTTTGGGCTTTTGTTATATAATCT | | |

| | | | |
|---|---|---|---|
| | CCTCTTTCTCTTTCTCTACCTGATTGGATTCAAGAACATAGCCAGATTTGGTAAAGTT<br>TATAAGATACAAAATATTAAGTAAGACTAAAGTAGAAATACATAATAACTTGAAAG<br>CTACTCTAAGTTATACAAATTCTAAAGAACTCAAAAGAATAACAAACAGTAGAAGT<br>TGGAAGCTCAAGCAATTAAATTATATAAAAACACTAACTACACTGAGCTGTCTCCTT<br>CTTCCACCAAATCTTGTTGCTGTCTCTTGAAGCTTTCTTATGACACAAACCTTAGACC<br>CAATTTCACTCACAGTTTGGTACAACCTCAGTTTTCTTCACAACAAATTCAAACATC<br>TTACCCTTATATTACCTCTTTATCTCTTCAATCATCAAAACACATAGTCACATACATT<br>TCTCTACCCCACCTTCTGCTCTGCTTCCGAGAGCTCAGTGTACCTCGCC | | |
| P_CYP86B1 | *Arabidopsis* | 2163 bp | Naseer, S., et al., (2012). Proc Natl Acad Sci USA 109:10101-10106. |
| | GGAGCTAAACACAACAAGTTTTCATGAGCACAAATAGTGGATGTATATACAAGAAT<br>TAATAACTTAGAAGGAAAAAAACTAGAGCTAAACTAAAGACTATCGTGTGTGATGG<br>TTCACAAATCCATATTAGAAATCAGACTTTGATTCTTCTGAGACAAAAAAAACCAA<br>CCTTGTATACATAAAGAGTTTGTTAACGTCTGCTTCAAACTGCAGTTGCCTTGGGTT<br>CTTAGCAAATGTGGTACTCTTGGCAAGAACTCCAACAGCCTGCAAATGCAAAGTTT<br>ATCAAATGAGCAACTTCATTAGAAATTTATCAATAAAAAACGAAACTTTACGATTC<br>CCCTTCAGCCTAATTTCAATTCTTCTAATCTTAATTCTATTGGTGCAAATCGAAAAG<br>ACTAAATCCGAACAAAAAATTCAAACTTTCTTCACACCCAGTAAGAGATCAAACAC<br>AGAAGCAAGAGATTCATTAACCTGCTGAAATTTGTTGAAAAGAGTCGCCATTTTTA<br>GTTTTCTTTCCTCTGAAACGATTCTCCACGCTCTTTGTTTATAAGGATTGCAAGAAAT<br>TACTGAAGAACATCAGGAATCGCGAGAGCTTGGGGCGAATCAAAAACCTCCATTGA<br>TGAAGAAGACTGCATCTAGTACTTTACTTACTCGGTAGCACACCTCCATGGGCAGGC<br>TCCAGCACCATGGTTGGGCCCATTTCTTATTAATGGGCCTAAAATAGAGCTTCTAGG<br>GCCTCTATATATTTTGTCTTTTCTCTTCCGTTTAGGCGTTTTGCTAACTTTCTTACGTA<br>TATGAATGTGAATTATAGTTAGTTAACTTTAGAACTGTTGAGCGGCCAATGCCACGT<br>CCTTTGTACGTAAATTATGTTATTCATGCTATTATTTATGTACCAATTTTCTATTCTA<br>ATATATATTCTAAGTTTTTTTTGTATCAATACTAAGTATTTATACAATTTTTGCAAAT<br>TTTGACAGAAAGATAGTACAAAAAATATGAAAGACCAAAAACTACATAAACCACC<br>AAAAATTACAACACAAACAGAAAACGGAAAGAAAACTCAATCGAAAACTCTACAA<br>GTAGAGACCTTAAAAGAAACTTAGATTCCAAACCACAAAGCTCAAATATTTGTGTG<br>GTTTCCTCAACTTAAAAGATCTCAAATACTTATAGTTGTTGCCACTAGCAATCCAAA<br>CCTAGGCCAGTTTTCAAAATCTCTAAAAAAGAGCTTACCATTCCAAACTTAGGACA<br>ATCCTCATAATATTTTAAATTTTATGACATTTTTTAGTTTTTTCTTACCGAACATAGA<br>GATACAACGAATAACACAAAATACCAAATTTTACTAAACAAACAATTACTGCACAA<br>ATGAAAAAGTAATCAACAACATGAGCTCATTTCACGAAAAAAGAAGCGAAAAAGC<br>CAACGGTCTTGCCACAAATTAGTATATTGCAAATTACAAATGCTTAAAAAAAATTCAC<br>AGATATATTCATCAATTATACCTTTTGATTATCTTGCAAATAAATCTTCCAATTCATA<br>TTACCTACGTCAAAATTTAGCCATCTACCCTTATTATCCCATTATGAAAATTTCAAA<br>ATCGATAAGATGACAGACACCCAACAACGAATTATCATTTTATATACTATAGGTATT<br>AGATACCTACAATTTATTTGGTTTATTGGGAATATACGATTCATAAATTCCCTCATA<br>AAAATATAAAGCAAATGTGGACGGAAGCAAAAAAACTAAATATAGAATGGAGAGA<br>AATTGTATTATTAACTTAGGTTACCCATCGCCAACAACCTCTCATTTCCTTTCCTAAA<br>CTATTTCACAATTTTGGTCAAAAACACAATCTAACCCCAAAAAAGATTCGACCTTTG<br>AAATTCCCAAAACCAGAAGCAAAACCCCCAAAAGAACCCTACTTATAGTGAAAGA | | |

| | | | |
|---|---|---|---|
| | AAAGTAACATAATAATATATATACACGAGAACCAAGAGCCACTTGAATATAAAGTT<br>CACAGTTTTTGTCTGCTCACCAAATCAAACCAATTTCTCCTGATAGCCAAAGTTTTT<br>GTCCCCAACAAGAAACTCATGCAACATTCTTCACCCACTAGCTAACTAACGACTTCC<br>ATTCTTATTAATCTCTCTCAATTCTTCATTTCTGAGATTCATCGCTCTCTCTTCTCTTT<br>GTCAC | | |
| P_CmRbc | *Chrysanthemum morifolium* | 1007 bp | Chung, S.M., et al., (2005). Trends Plant Sci10:357-361. |
| | CTTAGACAAACACCCCTTGTTATACAAAGAATTTCGCTTTACAAAATCAAATTCGAG<br>AAAATAATATATGCACTAAATAAGATCATTCGGATCTAATCTAACCAATTACGATA<br>CGCTTTGGGTACACTTGATTTTTGTTTCAGTGGTTACATATATCTTGTTTTATATGCT<br>ATCTTTAAGGATCTGCACAAAGATTATTTGTTGATGTTCTTGATGGGGCTCAGAAGA<br>TTTGATATGATACACTCTAATCTTTAGGAGATACCAGCCAGGATTATATTCAGTAAG<br>ACAATCAAATTTTACGTGTTCAAACTCGTTATCTTTTCATTCAAAGGATGAGCCAGA<br>ATCTTTATAGAATGATTGCAATCGAGAATATGTTCGGCCGATATGCCTTTGTTGGCT<br>TCAATATTCTACATATCACACAAGAATCGACCGTATTGTACCCTCTTTCCATAAAGG<br>AAAACACAATATGCAGATGCTTTTTTCCCACATGCAGTAACATATAGGTATTCAAAA<br>ATGGCTAAAAGAAGTTGGATAACAAATTGACAACTATTTCCATTTCTGTTATATAAA<br>TTTCACAACACACAAAAGCCCGTAATCAAGAGTCTGCCCATGTACGAAATAACTTC<br>TATTATTTGGTATTGGGCCTAAGCCCAGCTCAGAGTACGTGGGGGTACCACATATAG<br>GAAGGTAACAAAATACTGCAAGATAGCCCCATAACGTACCAGCCTCTCCTTACCAC<br>GAAGAGATAAGATATAAGACCCACCCTGCCACGTGTCACATCGTCATGGTGGTTAA<br>TGATAAGGGATTACATCCTTCTATGTTTGTGGACATGATGCATGTAATGTCATGAGC<br>CACAAGATCCAATGGCCACAGGAACGTAAGAATGTAGATAGATTTGATTTTGTCCG<br>TTAGATAGCAAACAACATTATAAAAGGTGTGTATCAATAGGAACTAATTCACTCAT<br>TGGATTCATAGAAGTCCATTCCTCCTAAGTATCTAGAAA | | |
| P_HSP18.2 | *Arabidopsis* | 915 bp | Takahashi, T., et al., (1989). Mol Gen Genet 219:365-372. |
| | AAGCTTGCTGCAGCTTTGACGACAAGTAGGTTTGTTCAATTAGTAGCATCTTTACAA<br>TGTAAAGCTTTTCTCTTCATTTCTCTTTCTTTCTTTCTTTCATGCAAGTTCTATTGATT<br>AGAAACCAAAACCATGACATATCACATTTTCAATTTTTAACCCCAAGTTTCCCAAAA<br>AGGTAATCACAATTACTTTCCATGGTCATTTCTTCTGGTTCAAGCATGACATGAACA<br>GGCAATAAATAAGTTGAGATTTTGATCACAGTAACTGATACTTGAATCGAATCATTT<br>AGATTTTTTTTTTTTTTAGTTTACTTGTTTAGTAAATATGTTGTCTATGTTTGTCACAA<br>AAACGTGGCTCAGTTCTTGTATATATGGAGACAAAAAAATCCATTAAAAGATTGTT<br>GACATTCTCGGAAATTTAGTGCCAACTGTTATTGCGAGAACTTACTATAGTTTTCCT<br>TTGGCGAAAAGCTAATAATCTTAAATCTTGATTTTGTCCTCTTTTCTCTGAGTTAGAT<br>TTTCTTAAATTCCACTTCCGACCTATTAAGAAATGGGCTTTTGCAAAGAAGATCCGC<br>TTCACTGAGCCCGTATCTCGAAGAGGATAATACAACAACAAAGCAAAACGGCACGT<br>AGTTTTAATTGTAACCAAGGATTGCATTTCGGTCTTGTTTCAACAAACGAAACTTCC<br>TGAAATGCCAAGAAAAATCTGGTCATTTCACCACAGTGATCATTGTGTATGTGTTCT<br>AAAGACTCCAAGCGAAGGTTTTAGAAAAAGGAGCATTTTCTATTCTATTCAAGAAA<br>CTCGAAGAACATTCTCTCTTCATCCTCTAACTTCCCTATAAATATGTCCTTTGCTAAT<br>CAGATCAAATCAGCAGGAAAATCAAGAACCAAAAGTCTCCCGAAAAGCAACGA | | |
| P_AtRD29A | *Arabidopsis* | 1525 bp | Bihmidine, S., et al., (2013). Planta 237:55-64. |

| | | | |
|---|---|---|---|
| | GGAGAGATTTGGGGTTTTGCTTTTGATGTTTGTGTTTTTGTATGATGCCTCTGTTTGTG<br>AACTTTGATGTATTTTATCTTTGTGTGAAAAAGAGATTGGGTTAATAAAATATTTGCTT<br>TTTTGGATAAGAAACTCTTTTAGCGGCCCATTAATAAAGGTTACAAATGCAAAATCAT<br>GTTAGCGTCAGATATTTAATTATTCGAAGATGATTGTGATAGATTTAAAATTATCCTA<br>GTCAAAAAGAAAGAGTAGGTTGAGCAGAAACAGTGACATCTGTTGTTTGTACCATAC<br>AAATTAGTTTAGATTATTGGTTAACATGTTAAATGGCTATGCATGTGACATTTAGACC<br>TTATCGGAATTAATTTGTAGAATTATTAATTAAGATGTTGATTAGTTCAAACAAAAAT<br>TTTATATTAAAAAATGTAAACGAATATTTTGTATGTTCAGTGAAAGTAAAACAAATTA<br>AATTAACAAGAAACTTATAGAAGAAAATTTTTACTATTTAAGAGAAAGAAAAAAATC<br>TATCATTTAATCTGAGTCCTAAAAACTGTTATACTTAACAGTTAACGCATGATTTGAT<br>GGAGGAGCCATAGATGCAATTCAATCAAACTGAAATTTCTGCAAGAATCTCAAACAC<br>GGAGATCTCAAAGTTTGAAAGAAAATTTATTTCTTCGACTCAAAACAAACTTACGAA<br>ATTTAGGTAGAACTTATATACATTATATTGTAATTTTTTGTAACAAAATGTTTTTATTA<br>TTATTATAGAATTTTACTGGTTAAATTAAAAATGAATAGAAAAGGTGAATTAAGAGG<br>AGAGAGGAGGTAAACATTTTCTTCTATTTTTTCATATTTTCAGGATAAATTATTGTAAA<br>AGTTTACAAGATTTCCATTTGACTAGTGTAAATGAGGAATATTCTCTAGTAAGATCAT<br>TATTTCATCTACTTCTTTTATCTTCTACCAGTAGAGGAATAAACAATATTTAGCTCCTT<br>TGTAAATACAAATTAATTTTCGTTCTTGACATCATTCAATTTTAATTTTACGTATAAAA<br>TAAAAGATCATACCTATTAGAACGATTAAGGAGAAATACAATTCGAATGAGAAGGAT<br>GTGCCGTTTGTTATAATAAACAGCCACACGACGTAAACGTAAAATGACCACATGATG<br>GGCCAATAGACATGGACCGACTACTAATAATAGTAAGTTACATTTTAGGATGGAATA<br>AATATCATACCGACATCAGTTTGAAAGAAAAGGGAAAAAAAGAAAAAATAAATAAA<br>AGATATACTACCGACATGAGTTCCAAAAAGCAAAAAAAAAGATCAAGCCGACACAG<br>ACACGCGTAGAGAGCAAAATGACTTTGACGTCACACCACGAAAACAGACGCTTCATA<br>CGTGTCCCTTTATCTCTCTCAGTCTCTCTATAAACTTAGTGAGACCCTCCTCTGTTTTA<br>CTCACAAATATGCAAACTAGAAAACAATCATCAGGAATAAAGGGTTTGATTACTTCT<br>ATTGGAAAGAAAAAAATCTTTGGA | | |
| P_AtRD29B | *Arabidopsis* | 649 bp | Bihmidine, S., et al., (2013). Planta 237:55-64. |
| | GCGTAATTTTCTAGATCCGTCTTGGGAGCTCAGACTGTATCAGTGATGATGATGATGA<br>TGAAGAAGAGAACGAATTTTGAAATTGGCGGTTTTGAATTTTTAAGAAATTAAAAAA<br>TATCCCCCGTCGATTTCAAGAGGGAGATGGAGATACCAAAGCAACTCTCGCCACTTGT<br>CGTCTTTTAATTTTAATTGAGTACGTTATGCCGTTTTAAATGTTCAAAACAGCACACA<br>GTTGATAGCTGAATTGATTTTTTCTTTTGCCGTTTTGTTATATTTAAACAACACACAGT<br>GCATTTGCCAAATAACTACATGATGGGCCAATAAACGTGGACCGACTAAAACTAAAT<br>AATAGAAGATACATCGATAGGCTTCTCTAAAGATCGGATAAAAGATAATGTCGCATA<br>GCCACGTAGAGAGCAACTGGCTGAGACGTGGCAGGACGAAACGGACGCATCGTACG<br>TGTCAGAATCCTACAGAAGTAAAGAGACAGAAGCCAGAGAGAGGTGGTTCGGCCAT<br>ATGTCATCGTTCTCTCTATAAACTTTATGGAACTTTGTTCTGATTTTCTCAGAGACACG<br>AAAAGAAAGAAAACAACACTAGAACAAAGAGGGTTTGATTGATTCACTTGAAAAAG<br>AGAAAACACAGCTTTGGA | | |
| P_MpUbiC-4 | *Marchantia polymorpha* | 2500 bp | |
| | GTATGAATCATTGTAGAGACTTGATTCCAGTGGACCTACTATACCTAACAAACGAAA<br>AAAACAGAACTCAGTCCAATGGAGCTCATGAAACCCTTGCAAACTCTCTTTAGTGAA | | |

| | | | |
|---|---|---|---|
| | AGTTTGAAACTTTGAAAATCACCAACAAACTATCCACAAATTTCTTGGAACTTTGGAG<br>ATCACAATTTTGATAATTGCAACAAGCTTTCCAATGATCAAAACGACAACAAGAAAG<br>AAAGACAAAATAAAAGTGAGACTTGAGCTTTAGTTTATCAATTTATTTTGAAATGCAT<br>CTTGTCACGATAATCATTAGTAGGTTATTGGGAAACACTTAAGCCTCAAAAGTACTCT<br>CATAGTAGTACTTCGTCCAAATAGGGGCAATCACTTTCTACACATTATAGCCGTCAAC<br>TATCGAGATACAAAAACTCGTTTTGGAACATGGTTGACGTAGGAGTTTTGGAGGGTC<br>TTCTCGTTTGAAAAAACTTCATGTTGGGTCCTAGCAAGTGTTTTGAATTCATTGTGTCT<br>TATCAATTTGTCTATTCGTTTGTATCTCTATTAACTATGTATGAAAATGAAACTAATCT<br>AGATTCAATTGATGCTCATTTGATTGAATGCTCATCGAAGGAAGAGAAGATATCTAT<br>ATATTATACCACCAGAAATATGATCGTGATGATGGCGAGTGAGAGTGTATTCCTCATT<br>CTGAGTTAGTGCTAATATACATTTTAGTTTTATCACGTTAAACACATATATTCTTGTTA<br>CAAGAGGCCCAAGTTATTGTTGCAAACAGGGTGATTATTGTAACTATATATAACAGA<br>TATAACGGAGATTGCTACGGTAGAAACCAATGTCTCAAAGTTTAAATATATGGTGAG<br>AGTTAATCTCCTTGGTTTTTTTCTCTAAAGATATAGAATTTTATGAATGCAAGATTTTA<br>CCACATTTTATTAATCTTTTTAAACAACATGTTTCCATTTTTTTACACATAACACTAGC<br>GAAATACCGTAGAGATCAGGTTAGATCATGAAACATCACATGATCTATTTGTTTATAG<br>ATTTTATATCTTGATCATGTGGATCTTTTAGCATGTCTTTCTCATTACTTCTACCTACA<br>GGTAAATATCATATTTGTACTACTTGATGGGATTTTTCAATATTCCCTTGCCCCATTCA<br>AAAATAACATCAATCATCATGCCTGTATACATACACATTCTTCTTGCCCCTCGCATGC<br>TTGCACGATGTTTGCCACCCACAGCATTGCGAAATATAGAATGTTGCTGTGAGATAA<br>GTATTTGCATATGCAACATTCCATTGCCAAGGAGTCACGTATTCCAGCACAGGCCTTG<br>TGGGAAAAAAGCCGGACATGGACGCTGCACTCCACATGTGATCAATATTCACTTATA<br>GAAATTATGCATTCGTTTCCTATATCCGAGAGAAGAGTGCAGAAACGTACCAGCCCG<br>TGCAATCTGCCAAGCCCGACGAGTTGCATGTAGAATGCACTGAGTGTCGTAAAACCA<br>CTGTAATGATTTGTTAGTACCATGTGTTCATTCCCCAGAGTACCAACGTGTTCAATAC<br>GAAGTTGAAATGACATTCTGCCATATAAAGTCACCATCGTAAAATATAAGGAACCAT<br>AATGAAGGTACTAAATTACACTTTTCAAGAGTACGTACGAGCGCTTTTGAAATGTTTG<br>CTGTGCGTCTGCCAATCATTCGTATGGAGAGATGAGGGTCCTCTGGGTCCTCTTACTT<br>ACCATTTGATTCGTTCTAGATTAGGCCTCATTCTCGTGAACGAAATAATAACAACCGC<br>GCGTATCGATTGGGTGGTGGGGTGTACTCGTGCTCAGCCACTGGAGGTCAGTGGGCG<br>TGCTCATCATGACCCGGAGAAAGACGAAATTCGAGCGTTCATGAAGACATTCATGCA<br>CACAAGGAAGGACCTCGCAGAAATATCTGGACGAGGAGCTGAGCCCACAGGAATTT<br>CAGGCAGATTTCCAGGAAGGTGAAGGCCGACCCGCTTTCGAAGGAAGGAAGGAAGG<br>AAGGAAGGAAGAATGACTAGATTGTTCCAAGTGAACAGAGCGCGAGATGCATCATG<br>ATTCTCAGGCTGAGGAGAATAGGACAGAGTAATAGTAAAAAAATAGGCTGCCAGTG<br>GTAAATGATCGCTCGTCTGTCTGGCCATCTCTGCCTGTCGTGCTGTACCGTCGTCGTC<br>ATTCATCGAGCAGCGAAGACTTGGAGAACAAGTTGAAGTCATGATTGACCCTACCCG<br>GGCGGTCACAAACGACGGGGTCCTTTGTTGTATAAGTACACGGGGCTGGGAATTGTA<br>GTTCAGCGGTTTCTGATTGTTGGTTATCATCGACACTGCTTCTTCCAGCAGTACTTCTA<br>CTACATAGAAATTTACGTCTAATAGTTTCGTTAATTTTGTTTCGTGGAGCAGGCAGAG<br>TACTGTCGATTCCATTTCGTTCGTGGATTGATTGATTGTCGTCCTAGTTGGAAAGGAA<br>GTGAGTGAGAAGAAGAAGGATC | | |
| P_MpUbiC-2 | *Marchantia polymorpha* | 2494 bp | |

TCGCTGTCCAGAGACACTTTTACAATTACATCCCTTAACATATTTTGGTTGAAACCTGT
GTTTTACCTCATAATAACTTGATTGCGAGACTACATGATCCATCCGTTAGGACAGCAC
AATTATCTACCATGTTGCACAAAAGAGGAAGAAACTATACTTCTAAATTTGGTACAAC
AACCAATGATGAATGGAATAAAATATACACAATTTGGTGAGAACGTTATTGATAGAA
ATAACTTATCTTCATGATTCAAAGTCTCCTGCAATCCGAGAGGAATCGACAACTTGAA
GACAACTTGAAGGGATCGGATGCTACGAGGATAGTTGGTGGCCTGTTCTACCCAGCT
GGCAGTGTTGACATGTTGAACACCAATTATATATCATATGATATTATTCTATCGAGCT
GAACGGTGAAGGAAACGTGGCCTGCTGCTAAATAAGACACCCACAATCTTTTCCCCC
AACATGACGGCAGGTCGTCGTGAGTTATCGGCAAGGACGACCGGGCAGTCCGGGCAG
CCACGATAAAGACGATCCAGGAGCCTGTACAATACGATAACTTTCGAAACAAGGTCC
AGGAACAAGGAATCTTCCTCTTGACTTCCTCAACCTGAGCCTCAAGCTGTTCCATGAC
GGTACTGTACTGTTCTGGAACGGAAGAGAGATGTTAGTCGTCAGTTGTCAGTGTTCGA
AAAATTCGGTTCTTCGGTGAAGATTCTCGAATTGGCTGCGATTTCAATTGTCAGAGTT
TCTTGTCAGGATTTATGTACGCCCAAGGTTTACCTCCTTCACTATTGACGGTGGTAAG
ATTCTGTGGAATATATTTCTAGACAAGGTTGGAAACTTTTTCTGAAGCTTTGTTTTCTT
CGACAAAAAGTTAGTTTGGTAGTCGAAGTTGATGAGATTTTGAGCGACATTTTCCAAA
TTTGTCTTATGTCATGTCACTATCTTTGGATGTCACTTGTTAAGTAAAAAGTCTGACAA
AATGTCGCAAAGTAACATGGATACATGCATTATCCTATACAGAATATATATATTTTCA
TTAAAATCACATAAATTAATGATACTACACTTTAGATGACGTCTCTTTGCCCCAACTT
CTATGTGGGTGATATAAACAAATTTTTCCTTGTCCGGTTCCAGGACCTCAAGTCTAGA
ACGCTTCACAAGAAAAATTTACCCTAAAACTCTCACCTGAATCCGATAATGTAGATTC
AAGGAAATGACGAAGGCGGGCTAGGTAAGATCAATTAGGATTAGAAGACCAAATCA
ATGAAGAAACCGAATAGAAACCAAGGGCTTCGCTCCGATTGTATATTGAAGTGGAGA
ATCGAAAGCATGATAACACAGGATTTGGAATTAAAACATCGATCAGTGTGCTAGACC
GCATTCTGGAAACCCGGATCCTTAGGTTTCCATTTAAATGGAATGTGGGGAACGCAGC
AATGTACAATTCTTCTAAATTAACACTAACTACACGTGGGATTTTCAATCTCAACTGA
TACAAATCCATGTATAATTACTACAGTCCAGATCATCCAGGTGCGGAAAAGAATTGC
AAAAGAGAACTCCCCCTCTCCCCTCCCATCCCCTTCCGACTCAAAGTCACGTCAGCCC
TCAGTTTACGCAAAATCACCAAGAGCATTTGAACCAGTAAGAACCGTTTGTAGTAGC
CTATCCGTTCCACTAAAATAACAACAAACGCAGAAATGGCTATTTTTGTAGTTCTAAT
TTTGTTTCTTAAACTATATATCTTTAATTTTGGGACTATAATAACGAATAGACATTAAC
ACTATTCCATTATTATGCGGCATGTCCACTAAAATAACAACAACGCGGAAAATGGTTA
CTTTTGTAGTTTCAAGTTTTTGTTGCTTAAAGTGAATATTTCAAATTGTATATCTTAAG
TGTTGGGACTATACTAACGATAATACATAAAAACTATTTTCGTATGGAACCATTATTA
GGCGACATTTTTACCGATACGATGGAACATACCAGAACGTACAAGAAAGAGAGAGA
AGAACGTGGCCCCCGTCCAGGCAGGGTGCGTGACCGGCAAGCAGAGGGGGAGATGA
GGAAGGTGGGGTGAGGTGAACCGGGAGTGACGGCAGAACCGGTCCCACCAGCAACG
TCACAGCGGGCAGAAGGCGAAGTTGAAGGCGAAGTTGCCGAGTACCGTCGGGCGGTC
AGTGTGTCAGTCACTCAGTGGACGAGTGTCGTCGTGATCAGTCTCGGTCCGGAGGGC
AAAGGTCGGGCAGACGGCAGAGCTCGGGGCAAGGGAAGAACGATATAAAGAGCATG
AAACGCCACCAGATTTACATTCCTCTCCTCTCTCCCTCTCTCTGTCTCCTCCTCCCTCTT
TCTCTCTCTGATCTGTTGTTCGGCGCGCGGCGCTCCACCGTCGCTCGATCCTCGCCGGG
ACACGGCCCTCGATCCATTGCCGCCCTGCTCGTGCTCGCTCGATCTGTCGTTCGGTTGT
CGTCGGGAGAAG

| | | |
|---|---|---|
| P_MpUbiC-3 | *Marchantia polymorpha* | 1500 bp |
| | ATAGAGTTGATCGAGAGATTCATGCCCGTTTTGAAACAGTGAGTAAGATATTAAGAA<br>TTATAATTTGTTATCTACTTGTTGCCACGTTGGTGACACGCTGTACCAGTACAGGTGCC<br>GGTTGAATACTATTTTAGCATGGGACGTACTTTTGACAGACCGTCGCTTGATTTTGGT<br>CCACATTCAATCAACTACATCTGAGTTTGCTTCATTAGCAGTGGCCGGTAGCGCCACC<br>GGCCACCAATTTCAATGTTTCAACTAAGTCTCGTTCGGGGTTTTCTAAATTATCTAGA<br>GTTTCGAGCTAAATCTACAGCGGGAATAAGTCACGACAGTTTAGAATAAGAGTCACT<br>ACAATTAGTAGAGCGTTTAGGGTCTAGATTACTTGTATTTGAGCTACAATATTGTTTTT<br>TTGGCGTCATTGTCTGCATCAAACGTGACACTCAGAGACGTGGATGAGGTTGTATCAT<br>TTTTCCCTTTGTTGTAAGTTTATAAGTTGTTTTTTCGCGGGCATATGTTGCATCAGCCC<br>GCTTAGAGTCAGATGATGATTAGATCCCAAATATATTATGTGAAATAAATTGATCCAA<br>AAAAGTGTTTGTAAACCTACATCTTAGAAAAAAAAATTATACATGTTTTGTCATCCAA<br>CTAATAGATCCACTTTTTCACCAATTATAAGGAAAAAGGCGCAAGTAAAATTATCCAT<br>TTCAAGATGCAAAGCAAATAAACCGTTCACTTCCGCTTCTGCACATATACCATAACAT<br>GACTTGCTAGTGGGCTTGGCGTGAAGCAAATACTTGCTACTATAGTCGGGCTTAGGAA<br>CAGCAGAGTATGAATCTGGAATAAACCCACGGGGGAGCCCTAAAACCTTTGATTTAG<br>AGAACTTAGGAAGTTCCCAGCTGTAGACTTGCGTAGTGCGACTTCTGGCCATGCCTAC<br>TGTAACCTCCTTGTACCGTTCACGTTCGACCATTTCTCATGATCAAAGAGTGGATCAC<br>ATGAAAAAAATGAGCAAATGATAATACATAATATTGTATCGCTGGTTTATTAAGGAG<br>AGTCAACTACTGTGCGCTGGAGCGGAGCATCATACGGCGGAGTCGTAGGCAGTTACT<br>GAACCGGAGCTCGTGGTGACGCTCTTCGTGGTTACTTGAGAAGTTTAACCCATCGTCA<br>TCCGGTTGCAGTAACTCACAAGTGGTCTGTCGGCAGCGGTGACTCACGGCAAGGTCC<br>AGGCGGGAGTCATGATTATCTGCATGTGGTGACGGCATGAGCTGTCAGCAGCTCGAG<br>GACCCGAAGAGCTATAAAGAACACCCGCTGGTCCTCATCTCCTCATCTCCTCATTCTG<br>GTCTGCTTCTTCTTTACCTCACAGCTTCATCTTTCTTTGCTCGTTAAAAGTCTATCTTTC<br>ACGTTCCTTTCTTCATTCGTTTCTACACTTTCTTCTTCGTCCGTTTGTTATTCTGTAGTG<br>TTCAGGAGGTGCACAACAATTCCACTTGGGGCCCGGTGCGCTCCGCCAAG | |
| P_MpEFla-5′UTR | *Marchantia polymorpha* | 1729 bp |
| | CAAATGAGTCACACACATTGTTGAGAGACATATCAAAATAACTCAATTGTAAGGTAA<br>GTTGATAGATTTCCCAGTTAAATGCATTATTTTAACAATATTTGTATCTCAAATTTTCA<br>AAATCATATCAAATTTGCAGATCTTGATGATGATTCACAACTCTGTCTTCGATTTTTGA<br>TTGTTAACGGGTATCGATATTTGGGTAACTTAAAAAGAATTTGCTAAAGATCTTAAAA<br>AACAGATTAAAAAAACCTTTCAAAATAGCTAATCCTTTAAATCCGTTTCACAATATAA<br>TGCTCATTGATGATAATCGAAAAGATTCCATTTTAAAGTGCATATAAATTGCTTAATT<br>ATCTCTTTCTGTCTCGTAATATTTTGTTCTCTCCTATTGGATCGGACCCGCATCTTCTCG<br>ATCAGTTCACTATACTAATATTTGGTCAACTTCAAGAGAGATTAGAACTTCGTTCTCC<br>AAAGTGTAAAGGCCGACAAGCAGAAAAGGAAACTTCAATCAGAAGCCTTCTAGGTTT<br>TCCCGCGTCCCAAAAAGCGAAGGGAGGACGGGGAGGACGGTGAGGCCAGAAGAAGA<br>AAGCAACAGTAAAATCTCATGTGATTGCGTGCATGTCATGGATCTATGGGATGCTAGT<br>CCACCGTTTGGAAGTATGTGCAGAAGGAGAAAGGCGCGGGAGGCCAAAAGCGACTT<br>CGGCCACCTCTCTACTTTCTAGAAAGTTTGCTCGCAGCTTGTAATAAGCGCGGAAAAG<br>GTACTCGAAAAACCGGAGCAATCGGTGTGGTATCAGCAGTCGGGTTCGCTAAACCTA | |

| | | | |
|---|---|---|---|
| | CGTCTTCTTATTTTGGAATGTTAAATTGAAAGGGCGTGCCGGCCCTAAGCACGGACGA<br>TGTGACAGACGGAGGAGTGTGAGCAATTCGGATGACGATGCTGGCCGGCAGTCACGA<br>CAAAGAGTCCGAAGGGGCATGGACAGGGCAATATATAAATGGAAAAGAGGCTTTGA<br>ATATTCCTAACCCTTCGTCGTCGGCCTCCCAACTTGCCCTCGCATGCTGAATTGGCCGC<br>CTATCTGCGAATGGCCGGCCGCCGACCCTAAGTCGCAATAACTTGAACGGAGCCGAT<br>GCAAAGTCCTTATCGAACGCTCGTTATTATAAACGGGGCAGACCTAGGTATTTAATTA<br>TATCAAAACCCTCCGCAATTTCGCTAATCATTCCCTAATTTCTTCTACTTTGGCGTCTA<br>GTGCTGTGGTCGCGCTTGCTCTCAAAGCTCGCTGGGAGGCAGTGGAGCGTCTGGCTTA<br>AAATCGATCGCCGCAACCCTAGAGTTCCTTTCACTTCTTTCTCTCTTGCGTGGAGAGC<br>ATCGACATCGAAGAGCTGCATCTTGTCTTCTGGTATGCGCCTTGTGCCCTCGTCTCTGT<br>ACGTCGTTACGATTGCTCGATATTGTGATTGTCTAGACCTGAAACCCGACTGGAGCTT<br>TTACGTGGTGTCGATTGGGGCCTCGAGCGAGTGATTTTTTAGGTTCGTTTTAGCCAAG<br>TACATCCCACTTTGGTCAGTCCTGTTATCTCGGTAAGGTGTTCAGTTCAGTCTCTGGTG<br>CGGAGGTGCCTCGTAAGTTTCCTGCATGATAGATTTGTCTATCCTTGACTTGAAGAGG<br>AGTCGAAGGTCAGCGAGGTTGGCTGTGTGCTATGGACGTCGGTGCTCCTTGGTTCCGT<br>GCTTCTATGGGTGTTGCCTCAACAGTATTGATGTGCCTGCAGAAAGGTTG | | |
| P_StGBSS | *Solanum tuberosum* | 857 bp | Ni, M., et al, (1995). Plant J 7:661-676. |
| | AAGCTTTAACGAGATAGAAAATTATATTACTCCGTTTTGTTCATTACTTAACAAATGC<br>AACAGTATCTTGTACCAAATCCTTTCTCTCTTTTCAAACTTTTCTATTTGGCTGTTGAC<br>AGAGTAATCAGGATACAAACCACAAGTATTTAATTGACTCATCCACCAGATATTATG<br>ATTTATGAATCCTCGAAAAGCCTATCCATTAAGTTCTCATCTATGGATATACTTGACA<br>GTTTCTTCCTATTTGGGTATTTTTTTTTCCTGCCAAGTGGAACGGAGACATGTTATGTT<br>GTATACGGGAAGCTCGTTAAAAAAAAAAAAATACAATAGGAAGAAATGTAACAAACA<br>TTGAATGTTGTTTTTAACCATCCTTCCTTTTAGCAGTGTATCAATTTTGTAATAGAACC<br>ATGCATCTCAATCTTAATACTAAAAAATGCAACAAAATTCTAGTGGAGGGACCAGTA<br>CCAGTACATTAGATATTATTTTTTATTACTATAATAATATTTTAATTAACACGAGACAT<br>AGGAATGTCAAGTGGTAGCGGTAGGAGGGAGTTGGTTTAGTTTTTTAGATACTAGGA<br>GACAGAACCGGAGGGGCCCATTGCAAGGCCCAAGTTGAAGTCCAGCCGTGAATCAAC<br>AAAGAGAGGGCCCATAATACTGTCGATGAGCATTTCCCTATAATACAGTGTCCACAG<br>TTGCCTTCCGCTAAGGGATAGCCACCCGCTATTCTCTTGACACGTGTCACTGAAACCT<br>GCTACAAATAAGGCAGGCACCTCCTCATTCTCACACTCACTCACTCACACAGCTCAAC<br>AAGTGGTAACTTTTACTCATCTCCTCCAATTATTTCTGATTTCATGCA | | |
| P_StUbi | *Solanum tuberosum* | 1197 bp | Ni, M., et al., (1995). Plant J 7:661-676. |
| | GGAGGGAATCTAATACTTACCTCTTAGAAATAAGAAAAAGTGTTTCTAATAGACCCTC<br>AATTTACATTAAATATTTTCAATCAAATTTAAATAACAAATATCAATATGAGGTCAAT<br>AACAATATCAAAATAATATGAAAAAAGAGCAATACATAATATAAGAAAGAAGATTT<br>AAGTGCGATTATCAAGGTAGTATTATATCCTAATTTGCTAATATTTAAACTCTTATATT<br>TAAGGTCATGTTCATGATAAACTTGAAATGCGCTATATTAGAGCATATATTAAAATAA<br>AAAAATACCTAAAATAAAATTAAGTTATTTTTAGTATATATTTTTTTACATGACCTAC<br>ATTTTTCTGGGTTTTTCTAAAGGAGCGTGTAAGTGTCGACCTCATTCTCCTAATTTTCC<br>CCACCACATAAAAATTAAAAAGGAAAGGTAGCTTTTGCGTGTTGTTTTGGTACACTAC<br>ACCTCATTATTACACGTGTCCTCATATAATTGGTTAACCCTATGAGGCGGTTTCGTCTA | | |

| | | | |
|---|---|---|---|
| | GAGTCGGCCATGCCATCTATAAAATGAAGCTTTCTGCACCTCATTTTTTCATCTTCTA<br>TCTGATTTCTATTATAATTTCTCTCAATTGCCTTCAAATTTCTCTTTAAGGTTAGAAAT<br>CTTCTCTATTTTTGGTTTTTGTCTGTTTAGATTCTCGAATTAGCTAATCAGGTGCTGTTA<br>TAGCCCTTAATTTTGAGTTTTTTTCGGTTGTTTTGATGGAAAAGGCCTAAAATTTGAG<br>TTTTTTTACGTTGGTTTGATGGAAAAGGCCTACAATTGGAGTTTTCCCCGTTGTTTTGA<br>TGAAAAAGCCCCTAGTTTGAGATTTTTTTCTGTCGATTCGATTCTAAAGGTTTAAAAT<br>TAGAGTTTTTACATTTGTTTGATGAAAAAGGCCTTAAATTTGAGTTTTCCGGTTGATT<br>TGATGAAAAAGCCCTAGAATTTGTGTTTTTCGTCGGTTTGATTCTGAAGGCCTAAAA<br>TTTGAGTTTCTCCGGCTGTTTTGATGAAAAAGCCCTAAATTTGAGTTTCTCCGGCTGTT<br>TTGATGAAAAAGCCCTAAATTTGAGTTTTTCCCCGTGTTTTAGATTGTTTGGTTTTAA<br>TTCTCGAATCAGCTAATCAGGGAGTGTGAAAAGCCCTAAAATTTGAGTTTTTTTCGTT<br>GTTCTGATTGTTGTTTTTATGAATTTGCAG | | |
| P_StPat | *Solanum tuberosum* | 1737 bp | Ni, M., et al., (1995). Plant J 7:661-676. |
| | AAGCTTATGTTGCCATATAGAGTAGTTTGTGATGGTATACTTCATAAACTTTAACTTA<br>TGTTAAATTTGTAATGATAAAATTTTTATTGTAAATTAAAAATTACTTATAAAATTGG<br>GCATTATAACATATGAAAGACAAATTGTGTTACATATTTTACTTTTGACTTTAATATG<br>AATATTTCAATTTAAATCATTGTTTTATTTTCTCTTTCTTTTTACAGGTATAAAAGGTG<br>AAAATTGAAGCAAGATTGATTGCAAGCTATGTGTCACCACGTTATTGATACTTTGGAA<br>GAAATTTTTACTTATATGTCTTTGTTTAGGAGTAATATTTGATATGTTTTAGTTAGATT<br>TTCTTGTCATTTATGCTTTAGTATAATTTTAGTTATTTTTATTATATGATCATGGGTGAA<br>TTTTGATACAAATATTTTTGTCATTAAATAAATTAATTTATCACAACTTGATTACTTTC<br>AGTGACAAAAAATGTATTGTCGTAGTACCCTTTTTTGTTGAATATGAATAATTTTTTT<br>ATTTTGTGACAATTGTAATTGTCACTACTTATGATAATATTTAGTGACATATATGTCGT<br>CGGTAAAAGCAAACACTTTCAGTGACAAAATAATAGATTTAATCACAAAATTATTAA<br>CCTTTTTTATAATAATAAATTTATCCCTAATTTATACATTTAAGGACAAAGTATTTTTT<br>TTATATATAAAAAATAGTCTTTAGTGACGATCGTAGTGTTGAGTCTAGAAATCATAAT<br>GTTGAATCTAGAAAAATCTCATGCAGTGTAAAATAAACCTCAAAAAGGACGTTCAGT<br>CCATAGAGGGGGTGTATGTGACACCCCAACCTCAGCAAAAGAAAACCTCCCTTCAAC<br>AAGGACATTTGCGGTGCTAAACAATTTCAAGTCTCATCACACATATATTTATTATATA<br>ATACTAATAAAGAATAGAAAAGGAAAGGTAAACATCATTAAATCGTCTTTGTATATT<br>TTTAGTGACAACTGATTGACGAAATCTTTTTCGTCACACAAAATTTTTAGTGACGAAA<br>CATGATTTATAGATGATGAAATTATTTGTCCCTCATAATCTAATTTGTTGTAGTGATCA<br>TTACTCCTTTGTTTGTTTTATTTGTCATGTTAGTCCATTAAAAAAAAATATCTCTCTTCT<br>TATGTACGTGAATGGTTGGAACGGATCTATTATATAATACTAATAAAGAATAGAAAA<br>AGGAAAGTGAGTGAGGTTCGAGGGAGAGAATCTGTTTAATATCAGAGTCGATCATGT<br>GTCAATTTTATCGATATGACCCTAACTTCAACTGAGTTTAACCAATTCCGATAAGGCG<br>AGAAATATCATAGTATTGAGTCTAGAAAAATCTCATGTAGTGTGGGGTAAACCTCAG<br>CAAGGACGTTGAGTCCATAGAGGGGGGTGTATGTGACACCCCAACCTCAGCAAAAGA<br>AAACCTCCCCTCAAGAAGGACATTTGCGGTGCTAAACAATTTCAAGTCTCATCACACA<br>TATATATATATTATATAATACTAATAAATAATAGAAAAAGGAAAGGTAAACATCACT<br>AACGACAGTTGCGGTGCAAACTGAGTGAGGTAATAAACATCACTAACTTTTATTGGTT<br>ATGTCAAACTCAAAGTAAAATTTCTCAACTTGTTTACGTGCCTATATATACCATGCTT<br>GTTATATGCTCAAAGCACCAACAAAATTTAAAAACACTTTGAACATTTGCAAA | | |

P_ZmUBQ1 *Zea mays* 1814 bp

GGAGGTAATGAGCATTGCATGTCTAAGTTATAAAAAATTACCACATATTTTTTTGTC
ACACTTGTTTGAAGTGCAGTTTATCTATCTTTATACATATATTTAAACTTTACTCTACG
AATAATATAATCTATAGTACTACAATAATATCAGTGTTTTAGAGAATCATATAAATGA
ACAGTTAGACATGGTCTAAAGGACAATTGAGTATTTTGACAACAGGACTCTACAGTTT
TATCTTTTTAGTGTGCATGTGTTCTCCTTTTTTTTTGCAAATAGCTTCACCTATATAATA
CTTCATCCATTTTATTAGTACATCCATTTAGGGTTTAGGGTTAATGGTTTTTATAGACT
AATTTTTTTAGTACATCTATTTTATTCTATTTTAGCCTCTAAATTAAGAAAACTAAAAC
TCTATTTTAGTTTTTTTATTTAATAATTTAGATATAAAATAGAATAAAATAAAGTGACT
AAAAATTAAACAAATACCCTTTAAGAAATTAAAAAAACTAAGGAAACATTTTTCTTG
TTTCGAGTAGATAATGCCAGCCTGTTAAACGCCGTCGACGAGTCTAACGGACACCAA
CCAGCGAACCAGCAGCGTCGCGTCGGGCCAAGCGAAGCAGACGGCACGGCATCTCTG
TCGCTGCCTCTGGACCCCTCTCGAGAGTTCCGCTCCACCGTTGGACTTGCTCCGCTGTC
GGCATCCAGAAATTGCGTGGCGGAGCGGCAGACGTGAGCCGGCACGGCAGGCGGCC
TCCTCCTCCTCTCACGGCACCGGCAGCTACGGGGGATTCCTTTCCCACCGCTCCTTCGC
TTTCCCTTCCTCGCCCGCCGTAATAAATAGACACCCCCTCCACACCCTCTTTCCCCAAC
CTCGTGTTGTTCGGAGCGCACACACACACAACCAGATCTCCCCCAAATCCACCCGTCG
GCACCTCCGCTTCAAGGTACGCCGCTCGTCCTCCCCCCCCCCCCCTCTCTACCTTCTCT
AGATCGGCGTTCCGGTCCATGGTTAGGGCCCGGTAGTTCTACTTCTGTTCATGTTTGTG
TTAGATCCGTGTTTGTGTTAGATCCGTGCTGCTAGCGTTCGTACACGGATGCGACCTG
TACGTCAGACACGTTCTGATTGCTAACTTGCCAGTGTTTCTCTTTGGGGAATCCTGGG
ATGGCTCTAGCCGTTCCGCAGACGGGATCGATTTCATGATTTTTTTTGTTTCGTTGCAT
AGGGTTTGGTTTGCCCTTTTCCTTTATTTCAATATATGCCGTGCACTTGTTTGTCGGGT
CATCTTTTCATGCTTTTTTTTGTCTTGGTTGTGATGATGTGGTCTGGTTGGGCGGTCGTT
CTAGATCGGAGTAGAATTCTGTTTCAAACTACCTGGTGGATTTATTAATTTTGGATCT
GTATGTGTGTGCCATACATATTCATAGTTACGAATTGAAGATGATGGATGGAAATATC
GATCTAGGATAGGTATACATGTTGATGCGGGTTTTACTGATGCATATACAGAGATGCT
TTTTGTTCGCTTGGTTGTGATGATGTGGTGTGGTTGGGCGGTCGTTCATTCGTTCTAGA
TCGGAGTAGAATACTGTTTCAAACTACCTGGTGTATTTATTAATTTTGGAACTGTATGT
GTGTGTCATACATCTTCATAGTTACGAGTTTAAGATGGATGGAAATATCGATCTAGGA
TAGGTATACATGTTGATGTGGGTTTTACTGATGCATATACATGATGGCATATGCAGCA
TCTATTCATATGCTCTAACCTTGAGTACCTATCTATTATAATAAACAAGTATGTTTTAT
AATTATTT

---

P_OsUbi *rice* 1484 bp Ma, X., et al. (2015). Mol Plant 8:1274-1284.

AGCCCCATTCACCACATTTGCTAGATAGTCGAAAAGCACCATCAATATTGAGCTTCAG
GTATTTTTGGTTGTGTTGTGGTTGGATTGATTCTAATATATACCAAATCAATATAATTC
ACTACCAAAATATACCATAGCCATCACAACTTTATTAATTTTGGTAGCTTAAGATGGT
ATATATAATAACCAATTAACAACTGATTCTAATTTTACTACGGCCCAGTATCTACCAA
TACAAAACAACGAGTATGTTTTCTTCCGTCGTAATCGTACACAGTACAAAAAAACCTG
GCCAGCCTTTCTTGGGCTGGGGCTCTCTTTCGAAAGGTCACAAAACGTACACGGCAGT
AACGCCGCTTCGCTGCGTGTTAACGGCCACCAACCCCGCCGTGAGCAAACGGCATCA
GCTTTCCACCTCCTCGATATCTCCGCGGCGCCGTCTGGACCCGCCCCCTTTCCGTTCCT
TTCTTTCCTTCTCGCGTTTGCGTGGTGGGGACGGACTCCCCAAACCGCCTCTCCCTCTC
TTTATTTGTCTATATTCTCACTGGGCCCCACCCACCGCACCCCTGGGCCCACTCACGA

GTCCCCCCCTCCCCACCTATAAATACCCCACCCCCTCCTCGCCTCTTCCTCCATCAATC
GAATCCCCAAAATCGCAGAGAAAAAAAAATCTCCCCTCGAAGCGAAGCGTCGAATCG
CCTTCTCAAGGTATGCGATTTTCTGATCCTCTCCGTTCCTCGCGTTTGATTTGATTTCCC
GGCCTGTTCGTGATTGTGAGATGTTGTGGTTAGTCTCCGTTTTGCGATCTGTGGTAGAT
TTGAACAGGGTTAGATGGGGTTCGCGTGGTATGCTGGATCTGTGATTATGAGCGATGC
TGTTCGTGGTCCAAGTATTGATTGGTTCGGATCTAGAAGTAGAACTGTGCTAGGGTTG
TGATTTGTTCCGATCTGTTCAATCAGTAGGATTTAGTCTCTGTTTTTCTCGTTGATCCA
AGTAGCAGCTTCAGGTATATTTTGCTTAGGTTGTTTTTGATTCAGTCCCTCTAGTTGCA
TAGATTCTACTCTGTTCATGTTTAATCTAAGGGCTGCGTCTTGTTGATTAGTGATTACA
TAGCATAGCTTTCAGGATATTTTACTTGCTTATGCCTATCTTATCAACTGTTGCACCTG
TAAATTCTAGCCTATGTTAATTAACCTGCCTTATGTGCTCTCGGGATAGTGCTAGTAGT
TATTGAATCAGTTTGCCGATGGAATTCTAGTAGTTCATAGACCTGCAGATTATTTTTGT
GAACTCGAGCACGGTGCGTCTCTCTATTTTGTTAGGTCACTGTTGGTGTTGATAGGTA
CACTGATGTTATTGTGGTTTAGATCGTGTATCTAACATATTGGAATAATTTGATTGACT
GATTTCTGCTGTACTTGCTTGGTATTGTTATAATTTCATGTTCATAGTTGCTGACCATG
CTTCGGTAATTGTGTGTGCAG

## Supplementary Table 2. Detailed information on the terminators used in this study

| Terminator | Length | Reference |
|---|---|---|
| T_AtUbq10 | 636 bp | |
| | ATCTCGTCTCTGTTATGCTTAAGAAGTTCAATGTTTCGTTTCATGTAAAACTTTGGT<br>GGTTTGTGTTTTGGGGCCTTGTATAATCCCTGATGAATAAGTGTTCTACTATGTTTC<br>CGTTCCTGTTATCTCTTTCTTTCTAATGACAAGTCGAACTTCTTCTTTATCATCGCT<br>TCGTTTTTATTATCTGTGCTTCTTTTGTTTAATACGCCTGCAAAGTGACTCGACTCT<br>GTTTAGTGCAGTTCTGCGAAACTTGTAAATAGTCCAATTGTTGGCCTCTAGTAATA<br>GATGTAGCGAAAGTGTTGAGCTGTTGGGTTCTAAGGATGGCTTGAACATGTTAAT<br>CTTTTAGGTTCTGAGTATGATGAACATTCGTTGTTGCTAAGAAATGCCTGTAATGT<br>CCCACAAATGTAGAAAATGGTTCGTACCTTTGTCCAAGCATTGATATGTCTGATGA<br>GAGGAAACTGCAAGATACTGAGCTTGGTTTAACGAAGGAGAGGCAGTTTCTTCCT<br>TCCAAAGCATTTCATTTGACAATGCCTTGATCATCTTAAGTAGAGTTTCTGTTGTG<br>GAAAGTTTGAAACTTTGAAGAAACGACTCTCAAGTAAATTGATGATCACAAGTGA<br>AAGTGTATGTTACATAAGT | |
| T_AtHsp70 | 607 bp | Sung, D.Y., et al.,. (2001). Plant Physiol 126:789-800. |
| | GCCTTTTGGCTTTTGTTTACTCTGTTTGCTTGAGATTCTAGTTGGTTTCTTGTTCTTA<br>GTTTTATCTTTCTATGTCACTCTGAAACTGGTGTGTGATCATTTTGATGCTTTAAGA<br>ATTTAGCTTTACCGTTTTTAAAACTCGCTCTGACCTATGAAAGACGACTGGGCATA<br>ATACTCTACACGAAATATAGTAGACAAAGTGAAAGAAGTTAAGTTCATGGTTTAA<br>GGAATATGATCTTAGCACTTTGATTTTATAATTAACCATCCAATATGAATCCAAGA<br>TCTCTTGTTATACATATTAAAGAAAATTTTCTTCTTATTTCAATATAGTTTATCTGA<br>AAAACCGTACAAATTGTAGTGGGATTCCATATTCTATATATGAGATGTAAGATTTC<br>ATTTGTTGTTGTCCAAAAAAAAAAAATGATTTCATTTGTTAAAGAGTCTTCCATTGA<br>ATGTTGTCACATATTGCGTAACACAAATTCAAGCATATGAACAAATAAGGAATTA<br>AGAGGTTAAAAGAACACAATGAAATGGAGTAAATATGATTAAATATGAATAATC<br>CAATGATACATGAACAATAAAATTCTTTTAAGAAGTTATGCGC | |
| T_AtHsp18.2 | 238 bp | Farhi, M., et al. (2011). Nat Biotechnol 29:1072-1074 |
| | ATATGAAGATGAAGATGAAATATTTGGTGTGTCAAATAAAAAGCTTGTGTGCTTA<br>AGTTTGTGTTTTTTTCTTGGCTTGTTGTGTTATGAATTTGTGGCTTTTTCTAATATTA<br>AATGAATGTAAGATCTCATTATAATGAATAAACAAATGTTTCTATAATCCATTGTG<br>AATGTTTTGTTGGATCTCTTCTGCAGCATATAACTACTGTATGTGCTATGGTATGG<br>ACTATGGAA | |
| T_AtAct2 | 662 bp | Sarrion-Perdigones, A., et al., (2013). Plant Physiol 162:1618-1631. |
| | GCTCTCAAGATCAAAGGCTTAAAAAGCTGGGGTTTTATGAATGGGATCAAAGTTT<br>CTTTTTTTCTTTTATATTTGCTTCTCCATTTGTTTGTTTCATTTCCCTTTTTGTTTTCG<br>TTTCTATGATGCACTTGTGTGTGACAAACTCTCTGGGTTTTTACTTACGTCTGCGTT<br>TCAAAAAAAAAAAACCGCTTTCGTTTTGCGTTTTAGTCCCATTGTTTTGTAGCTCTG<br>AGTGATCGAATTGATGCCTCTTTATTCCTTTTGTTCCCTATAATTTCTTTCAAAACT<br>CAGAAGAAAAACCTTGAAACTCTTTGCAATGTTAATATAAGTATTGTATAAGATTT<br>TTATTGATTTGGTTATTAGTCTTACTTTTGCTACCTCCATCTTCACTTGGAACTGAT<br>ATTCTGAATAGTTAAAGCGTTACATGTCTTCCATTCACAAATGAACTTAAACTAGC<br>ACAAAGTCAGATATTTTAAGATCGCACCATTTTATATAACCCCAATCGTCAATTCT<br>ACTGTTTCAAGTTTTACACCAAAACAATTACGAGGTGTATCTATTCGTTACTCTTTT | |

| | | |
|---|---|---|
| | | CGCTTATTAATCCTTTTACATGATGCAAAATATAACAAATTAATTTCAAATTGTCC<br>TGCACATTTGGTTTATACATCTTGATTCCAAAAAGT |
| T_AtG7 | 298 bp | Butaye, K.M., et al., (2004). Plant J 39:440-449.<br>GCTTACTGACTAACTAGGATGAGCTAAGCTAGCTATATCATCAATTTATGTATTAC<br>ACATAATATCGCACTCAGTCTTTCATCTACGGCAATGTACCAGCTGATATAATCAG<br>TTATTGAAATATTTCTGAATTTAAACTTGCATCAATAAATTTATGTTTTTGCTTGGA<br>CTATAATACCTGACTTGTTATTTTATCAATAAATATTTAAACTATATTTCTTTCAAG<br>ATGGGAATTAACATCTACAAATTGCCTTTTCTTATCGACCATGTACCCCGGGTACC<br>AAGCTTCTCGAG |
| T_At3g24240 | 722 bp | Shinohara, H., et al., (2016). Proc Natl Acad Sci USA 113:3897-3902.<br>TTTTAAGGTTTGAAGGTTACGTGAGAAGAAAGCTTTTGTGTGTTGTTTGAGAAAAT<br>GGCCTTTTTTTTTCATTTATCATTTTAAGTCTTTATATATGTTATGTATAAAAGAAG<br>AAGAATAGAATGAGAGAAGTGAGTCAGTAATGTTATGGAATTGTGATTAGTAAAG<br>GGTGACATGATATTGTACCTTTTTGGGCGGTTTGAAATCAATAATTTCTCTAGTTTT<br>CTCTCCAAGTGTGACTTTAGATTGAATGTAGTCCTTTATAGTTTTCACTCATAAAC<br>AGTACTATATATATCACCTTCCAGAAACGTATTAATAATAATATTGAATTATTGAT<br>GCATAAAACAATTCTTCAAAAATGATATTTCATGGGGCTCTTGAATCAGTAAGCC<br>ACATAATATTGGACCATGTTTTGGTTTACATGCAACATTATATTGCATACCGTCCG<br>GCCAATTGAGTTGGTCTCTATTTTCTTTCTTCGTGTGGCCAATTTTGGCACAATGAA<br>AAAGCATGCATTTGACCTTTTTCATTTTTTAAACCTTGAGCCTTTCATATATTCTTA<br>ATCTTACTATAATTGTTGATCACCAAATACATTTTTGAGAGCTAGAGCTGGTAAAT<br>ACGTTTAGCTCTATTTCCATTCATTATAATTTGGAAAAATTGGATATTAAGGATCA<br>TTTTTTCAAAAATTTGTCCATCTATACCTTAGTTTGTAAATTGT |
| T_nos | 257 bp | Farhi, M., et al. (2011). Nat Biotechnol 29:1072-1074<br>CGTTCAAACATTTGGCAATAAAGTTTCTTAAGATTGAATCCTGTTGCCGGTCTTGC<br>GATGATTATCATATAATTTCTGTTGAATTACGTTAAGCATGTAATAATTAACATGT<br>AATGCATGACGTTATTTATGAGATGGGTTTTTATGATTAGAGTCCCGCAATTATAC<br>ATTTAATACGCGATAGAAAACAAAATATAGCGCGCAAACTAGGATAAATTATCGC<br>GCGCGGTGTCATCTATGTTACTAGATCGGG |
| T_ocs | 730 bp | Farhi, M., et al. (2011). Nat Biotechnol 29:1072-1074<br>ACTAGTCCCTAGAGTCCTGCTTTAATGAGATATGCGAGACGCCTATGATCGCATG<br>ATATTTGCTTTCAATTCTGTTGTGCACGTTGTAAAAAACCTGAGCATGTGTAGCTC<br>AGATCCTTACCGCCGGTTTCGGTTCATTCTAATGAATATATCACCCGTTACTATCG<br>TATTTTTATGAATAATATTCTCCGTTCAATTTACTGATTGTACCCTACTACTTATAT<br>GTACAATATTAAAATGAAAACAATATATTGTGCTGAATAGGTTTATAGCGACATC<br>TATGATAGAGCGCCACAATAACAAACAATTGCGTTTTATTATTACAAATCCAATTT<br>TAAAAAAAGCGGCAGAACCGGTCAAACCTAAAAGACTGATTACATAAATCTTATT<br>CAAATTTCAAAAGTGCCCCAGGGGCTAGTATCTACGACACACCGAGCGGCGAACT<br>AATAACGCTCACTGAAGGGAACTCCGGTTCCCCGCCGGCGCGCATGGGTGAGATT<br>CCTTGAAGTTGAGTATTGGCCGTCCGCTCTACCGAAAGTTACGGGCACCATTCAAC<br>CCGGTCCAGCACGGCGGCCGGGTAACCGACTTGCTGCCCCGAGAATTATGCAGCA<br>TTTTTTTGGTGTATGTGGGCCCCAAATGAAGTGCAGGTCAAACCTTGACAGTGACG<br>ACAAATCGTTGGGCGGGTCCAGGGCGAATTTTGCGACAACATGTCGAGGCTCAGC<br>AGGA |

| | | |
|---|---|---|
| T_mas | 265 bp | Chung, S.M., et al., (2005). Trends Plant Sci 10:357-361.<br>AATCTTGGACTCCCATGTTGGCAAAGGCAACCAAACAAACAATGAATGATCCGCT<br>CCTGCATATGGGGCGGTTTGAGTATTTCAACTGCCATTTGGGCTGAATTGAAGACA<br>TGCTCCTGTCAGAAATTCCGTGATCTTACTCAATATTCAGTAATCTCGGCCAATAT<br>CCTAAATGTGCGTGGCTTTATCTGTCTTTGTATTGTTTCATCAATTCATGTAACGTT<br>TGCTTTTCTTATGAATTTTCAAATAAATTATCAGATC |
| T_35S | 215 bp | Chung, S.M., et al., (2005). Trends Plant Sci 10:357-361.<br>GTCCGCAAAAATCACCAGTCTCTCTCTACAAATCTATCTCTCTCTATTTTTCTCCAG<br>AATAATGTGTGAGTAGTTCCCAGATAAGGGAATTAGGGTTCTTATAGGGTTTCGCT<br>CATGTGTTGAGCATATAAGAAACCCTTAGTATGTATTTGTATTTGTAAAATACTTC<br>TATCAATAAAATTTCTAATTCCTAAAACCAAAATCCAGTGAC |
| T_rbc | 922 bp | Chung, S.M., et al., (2005). Trends Plant Sci 10:357-361.<br>TCATAAGCCCGATGGCTACTAAGTTTTACTATTTACCAAGACTTTTGAATATTAAC<br>CTTCTTGTAACGAGTCGGTTAAATTTGATTGTTTAGGGTTTTGTATTATTTTTTTTT<br>GGTCTTTTAATTCATCACTTTAATTCCCTAATTGTCTGTTCATTTCGTTGTTTGTTTC<br>CGGATCGATAATGAAATGTAAGAGATATCATATATAAATAATAAATTGTCGTTTC<br>ATATTTGCAATCTTTTTTTTACAAACCTTTAATCGTTGTATGTATGACATTTTCTTC<br>TTGTTATATTAGGGGGAAATAATGTTAAATAAAAGTACAAAATAAACTACAGTAC<br>ATCGTACTGAATAAATTACCTAGCCAAAAAGTACACCTTTCCATATACTTCCTACA<br>TGAAGGCATTTTCAACATTTTCAAATAAGGAATGCTACAACCGCATAATAACATC<br>CACAAATTTTTTTATAAAATAACATGTCAGACAGTGATTGAAAGATTTTATTATAG<br>TTTCGTTATCTTCTTTTCTCATTAAGCGAATCACTACCTAACACGTCATTTTGTGAA<br>ATATTTTTTGAATGTTTTTATATAGTTGTAGCATTCCTCTTTTCAAATTAGGGTTTG<br>TTTGAGATAGCATTTCAGCCGGTTCATACAACTTAAAAGCATACTCTAATGCTGGA<br>AAAAAGACTAAAAAATCTTGTAAGTTAGCGCAGAATATTGACCCAAATTATATAC<br>ACACATGACCCCATATAGAGACTAATTACACTTTTAACCACTAATAATTATTACTG<br>TATTATAACATCTACTAATTAAACTTGTGAGTTTTTGCTAGAATTATTATCATATAT<br>ACTAAAAGGCAGGAACGCAAACATTGCCCCGGTACTGTAGCAACTACGGTAGAC<br>GCATTAATTGTCTATAGTGGA |
| T_ags | 406 bp | Farhi, M., et al., (2011). Nat Biotechnol 29:1072-1074<br>GAATTAACAGAGGTGGATGGACAGACCCGTTCTTACACCGGACTGGGCGCGGGAT<br>AGGATATTCAGATTGGGATGGGATTGAGCTTAAAGCCGGCGCTGAGACCATGCTC<br>AAGGTAGGCAATGTCCTCAGCGTCGAGCCCGGCATCTATGTCGAGGGCATTGGTG<br>GAGCGCGCTTCGGGGATACCGTGCTTGTAACTGAGACCGGATATGAGGCCCTCAC<br>TCCGCTTGATCTTGGCAAAGATATTTGACGCATTTATTAGTATGTGTTAATTTTCAT<br>TTGCAGTGCAGTATTTTCTATTCGATCTTTATGTAATTCGTTACAATTAATAAATAT<br>TCAAATCAGATTATTGACTGTCATTTGTATCAAATCGTGTTTAATGGATATTTTTAT<br>TATAATATTGATGAT |
| T_3'utr-nos | 495 bp | Peyret, H., et al., (2013). Plant Mol Biol 83:51-58.<br>TTAACTCTGGTTTCATTAAATTTTCTTTAGTTTGAATTTACTGTTATTCGGTGTGCA<br>TTTCTATGTTTGGTGAGCGGTTTTCTGTGCTCAGAGTGTGTTTATTTTATGTAATTT<br>AATTTCTTTGTGAGCTCCTGTTTAGCAGGTCGTCCCTTCAGCAAGGACACAAAAAG<br>ATTTTAATTTTATTAAAAAAAAAAAAAAAAAAAAAGACCGGGAATTCGATATCAAG<br>CTTATCGACCTGCAGATCGTTCAAACATTTGGCAATAAAGTTTCTTAAGATTGAAT |

CCTGTTGCCGGTCTTGCGATGATTATCATATAATTTCTGTTGAATTACGTTAAGCA
TGTAATAATTAACATGTAATGCATGACGTTATTTATGAGATGGGTTTTTATGATTA
GAGTCCCGCAATTATACATTTAATACGCGATAGAAAACAAAATATAGCGCGCAAA
CTAGGATAAATTATCGCGCGCGGTGTCATCTATGTTACTAGATCTCT

**Supplementary Table 3. Primers used in this study.**

| Primer | Sequence (5′→3′) |
|---|---|
| GG3-BsaI-F | AGTGAATTGTTAATGGAGAGAGACCCCGATGGAAACGTTTAAGCAGG TCTCCCGCTCGTAATCATGGTCATA |
| GG3-BsaI-R | TATGACCATGATTACGAGCGGGAGACCTGCTTAAACGTTTCCATCGGG GTCTCTCTCCATTAACAATTCACT |
| 43-BsaI-F | CTTTTAAGAAGTTATGCGCCGCTGGAGAGAGACCCCGATGGAAAC |
| 43-inter-R | CAAGCTGGAAACCTTCCGC |
| 43-inter-F | GCGGAAGGTTTCCAGCTTG |
| 43-BsaI-R | CAGCTATGACCATGATTACGAGCGGGAGACCTGCTTAAACGTTTCCAT CGGGGTCTCTCTCCAGCGGCGCATAACTTCTT |
| GFP208-F | TGCAGTGCTTCAGCCGCTA |
| GFP494-R | AGTTCACCTTGATGCCGTTC |
| qP19-F | GACTGAGTGGCGGCTACAT |
| qP19-R | TCCTCCAGAAACGGTGATAC |
| Fluc-F-635 | ATAGAACTGCCTGCGTGAG |
| Fluc-R-886 | CGAAGAAGGAGAATAGGGTT |
| REN-F-194 | CACATATTGAGCCAGTAGCG |
| REN-R-476 | ATATCAGGCCATTCATCCC |
| P_CaMV35S_RmBsa-F | GGAATTCGGTCTCAGGAGGGAAACCTCCTCGGATTCC |
| P_CaMV35S_RmBsa-R | CGGGATCCGGTCTCACATTCCTCTCCAAATGAAATGAAC |
| P_CsVMV-F | GGAATTCGGTCTCAGGAGCCAGAAGGTAATTATCCAAG |
| P_CsVMV-R | CGGGATCCGGTCTCACATTACAAACTTACAAATTTCTCTG |
| P_35S-5’UTR-F | CGGTCTCAGGAGGGAAACCTCCTCGGATTCCAT |
| P_35S-5’UTR-R | CGGTCTCACATTCGCGAATTTGGGCAGAATATACAG |
| P_MAS-1-F | GGAATTCGGTCTCAGGAGTTTTCAAATCAGTGCGCAAG |
| P_MAS-1-R | CGGGATCCGGTCTCACATTCGATTTGGTGTATCGAGATTGG |
| P_Ocs-1-F | GGAATTCGGTCTCAGGAGATCATTAATTTCCACCTTCAC |
| P_Ocs-1-R | CGGGATCCGGTCTCACATTTTGGTAGATTGCAAATATAATG |
| P_AtAct 2-F | GGAATTCGGTCTCAGGAGTCGACAAAATTTAGAACGAAC |
| P_AtAct 2-R | CGGGATCCGGTCTCACATTTTCAAAGCGGAGAGGAAAATATATG |
| P_AtFBA2-F | GGAATTCGGTCTCAGGAGCCTACACATCCACTTGGCC |
| P_AtFBA2-R | CGGGATCCGGTCTCACATTTTTTTTTTGTGTGTGTTATC |
| P_AtRbcs1a-F | GGAATTCGGTCTCAGGAGAACATTCATACGTTTTCATTTG |
| P_AtRbcs1a-R | CGGGATCCGGTCTCACATTTGTTCTTCTTTACTCTTTGTG |
| P_AtUbq10-F | GGAATTCGGTCTCAGGAGTACCCGACGAGTCAGTAATAAAC |
| P_AtUbq10-R | CGGGATCCGGTCTCACATTAGTGTTAATCAGAAAAACTC |
| P_AtHsp18-F | GGAATTCGGTCTCAGGAGAAGCTTTTCTCTTCATTTCTC |
| P_AtHsp18-R | CGGGATCCGGTCTCACATTGACTTTTGGTTCTTGATTTTCC |
| P_AtHsp70-F | GGAATTCGGTCTCAGGAGGAACTGCGAAAAAAGGGAGC |
| P_AtHsp70-R | CGGGATCCGGTCTCACATTTGCTAAAAAAAAGCTTCAGTAATTG |
| P_At2S3-F | GGAATTCGGTCTCAGGAGGAAACCAAATTAACATAGG |
| P_At2S3-R | CGGGATCCGGTCTCACATTGTTTTGCTATTTGTGTATGTTTTC |
| P_AtBch1-F | GGAATTCGGTCTCAGGAGTGCCTTAATCTTGTCTGCAATG |

| | |
|---|---|
| P_AtBch1-R | CGGGATCCGGTCTCACATTCCTAATGGAAGGAGGAGCTAC |
| P_AtUbq3-F | ATGGTCTCAGGAGCAGAGCTAAATTCCAAAAAAAATATG |
| P_AtUbq3-R | ATGGTCTCACATTCTGAAATAAAACAATAGAACAAG |
| P_At3g24240-F | ATGGTCTCAGGAGGAAAAAGTGATGGAAACTCGG |
| P_At3g24240-R | ATGGTCTCACATTTTGCCTCAAAATACGAAAAGAAG |
| P_AtJAL34-F | GGAATTCGGTCTCAGGAGGCTAATGACATTAGATCG |
| P_AtJAL34-R | CGGGATCCGGTCTCACATTCGTTGTTGACTTCTTTTATC |
| P_AtWRKY6-F | GGAATTCGGTCTCAGGAGGTCGGTGACAAGCAACGAAAC |
| P_AtWRKY6-R | CGGGATCCGGTCTCACATTATATAGAAAAAGAAAGAGATCACG |
| P_AtHY5-F | GGAATTCGGTCTCAGGAGGAGCATTGGGTGTTGGTG |
| P_AtHY5-R | CGGGATTCGGTCTCACATTTTTTCTTACTCTTTGAAGATCG |
| P_CasP1-F | GGAATTCGGTCTCAGGAGAATAACGCATGCATTGTATTTGTATTC |
| P_CasP1-R | CGGGATTCGGTCTCACATTTTCTCTTGCAATTGGGGTTTAAAAG |
| P_DAISY-F | GGAATTCGGTCTCAGGAGATTAAATGCTCGCTTTGATTG |
| P_DAISY-R | CGGGATCCGGTCTCACATTGGTAGGTTTTTTGGTTTTAAATG |
| P_KCR1-F | GGAATTCGGTCTCAGGAGCCAAGAAAGTTGGAAGAGGAAG |
| P_KCR1-R | CGGGATCCGGTCTCACATTAGAGAAGAAAGGTTGAGACTTTGG |
| P_HORST-F | GGAATTCGGTCTCAGGAGTATGCATCGATGATGATTCTGCC |
| P_HORST-R | CGGGATCCGGTCTCACATTATCCCGGTTTAGGCTTTTTGC |
| P_GPAT5-F | GGAATTCGGTCTCAGGAGTGATCGCAAACGTCAATG |
| P_GPAT5-R | CGGGATCCGGTCTCACATTTCTTTTGTTTTTTGCTCG |
| P_ASTF-F | GGAATTCGGTCTCAGGAGCAGGGTTCTTCATCATCATTCAC |
| P_ASTF-R | CGGGATCCGGTCTCACATTTTGATCCAAATGGAGAAAACAGC |
| P_FAR4-F | GGAATTCGGTCTCAGGAGAGTTCTAACCCCGACATAAGG |
| P_FAR4-R | CGGGATCCGGTCTCACATTGAAGAAACTTATATCTATCC |
| P_AtIRX5-F | GGAATTCGGTCTCAGGAGATGAAGCCATCCTCTACCTCGG |
| P_AtIRX5-R | CGGGATCCGGTCTCACATTGGCGAGGTACACTGAGCTC |
| P_CYP86B1-F | GGAATTCGGTCTCAGGAGCTAAACACAACAAGTTTTCATGAG |
| P_CYP86B1-R | CGGGATCCGGTCTCACATTGTGACAAAGAGAAGAGAGAGCG |
| P_AtRD29A-F | ATTGGTCTCGGGAGGAGATTTGGGGTTTTGCTTTTG |
| P_AtRD29A-R | ATTGGTCTCACATTTCCAAAGATTTTTTTCTTTCC |
| P_AtRD29B-F | ATTGGTCTCGGGAGGCGTAATTTTCTAGATCCGTCTTGG |
| P_AtRD29B-R | ATTGGTCTCACATTTCCAAAGCTGTGTTTTCTC |
| P_MpUbiC-4-F | ATTGGTCTCGGGAGGGTATGAATCATTGTAGAGAC |
| P_MpUbiC-4-R | ATTGGTCTCACATTGATCCTTCTTCTTCTCACTCAC |
| P_MpUbiC-2-F | ATTGGTCTCGGGAGGTCGCTGTCCAGAGACACTTTTAC |
| P_MpUbiC-2-R | ATTGGTCTCACATTCTTCTCCCGACGACAACCG |
| P_MpUbiC-3-F | ATTGGTCTCGGGAGGATAGAGTTGATCGAGAGATTC |
| P_MpUbiC-3-R | ATTGGTCTCACATTCTTGGCGGAGCGCACCGG |
| P_MpEFla-5′UTR-F | ATTGGTCTCGGGAGGCAAATGAGTCACACACATTG |
| P_MpEFla-5′UTR-R | ATTGGTCTCACATTCAACCTTTCTGCAGGCAC |
| P_StGBSS-F | ATTGGTCTCGGGAGAAGCTTTAACGAGATAGAAAATTATATTAC |
| P_StGBSS-R | ATTGGTCTCACATTTGCATGAAATCAGAAATAATTGGAG |
| P_StUbi-F | ATTGGTCTCGGGAGGGAATCTAATACTTACCTCTTAG |

| | |
|---|---|
| P_StUbi-R | ATTGGTCTCACATTCTGCAAATTCATAAAAACAAC |
| P_StPat-F | ATTGGTCTCGGGAGAAGCTTATGTTGCCATATAGAG |
| P_StPat-R | ATTGGTCTCACATTTTTGCAAATGTTCAAAGTGTTTTTAAATTTTG |
| P_ZmUBQ1-F | ATTGGTCTCGGGAGGTAATGAGCATTGCATGTCTAAGTTATAAAAAATTACC |
| P_ZmUBQ1-R | ATTGGTCTCACATTAAATAATTATAAAACATACTTGTTTATTATAATAGATAGGTACTC |
| P_OsUbi-F | ATTGGTCTCGGGAGAGCCCCATTCACCACATTTG |
| P_OsUbi-R | ATTGGTCTCACATTCTGCACACACAATTACC |
| T_AtUbq10-F | GGAATTCGGTCTCAGCTTATCTCGTCTCTGTTATGCT |
| T_AtUbq10-R | CGGGATCCGGTCTCAAGCGCACTTATGTAACATACACTTTC |
| T_AtHsp70-F | GGAATTCGGTCTCAGCTTGCCTTTTGGCTTTTGTTTACTC |
| T_AtHsp70-R | CGGGATCCGGTCTCAAGCGGCGCATAACTTCTTAAAAG |
| T_AtHsp18.2-F | GGAATTCGGTCTCAGCTTATATGAAGATGAAGATGAAATATTTG |
| T_AtHsp18.2-R | CGGGATCCGGTCTCAAGCGTTCCATAGTCCATACCATAGCAC |
| T_AtAct2-F | GGAATTCGGTCTCAGCTTGCTCTCAAGATCAAAGGCTTAAAAAG |
| T_AtAct2-R | CGGGATCCGGTCTCAAGCGACTTTTTGGAATCAAGATGTATAAAC |
| T_AtG7-F | GGAATTCGGTCTCAGCTTACTGACTAACTAGGATGAGCTAAG |
| T_AtG7-R | CGGGATCCGGTCTCAAGCGCTCGAGAAGCTTGGTACCCG |
| T_At3g24240-F | GGAATTCGGTCTCAGCTTTTTTAAGGTTTGAAGGTTACGTG |
| T_At3g24240-R | CGGGATCCGGTCTCAAGCGACAATTTACAAACTAAGGTATAG |
| T_nos-F | GGAATTCGGTCTCAGCTTCGTTCAAACATTTGGCAATAAAG |
| T_nos-R | CGGGATCCGGTCTCAAGCGCCCGATCTAGTAACATAGATGAC |
| T_3'utr-nos-F | TTCGGTCTCAGCTTTTAACTCTGGTTTCATTAAATTTTC |
| T_3'utr-nos-R | TCCGGTCTCAAGCGAGAGATCTAGTAACATAGATG |
